# Tuber*omics*: a molecular profiling for the adaption of edible fungi (*Tuber magnatum* Pico) to different natural environments

**DOI:** 10.1186/s12864-020-6522-3

**Published:** 2020-01-29

**Authors:** Federico Vita, Beatrice Giuntoli, Edoardo Bertolini, Cosimo Taiti, Elettra Marone, Chiara D’Ambrosio, Emanuela Trovato, Danilo Sciarrone, Mariosimone Zoccali, Raffaella Balestrini, Andrea Scaloni, Luigi Mondello, Stefano Mancuso, Massimo Alessio, Amedeo Alpi

**Affiliations:** 1 Dipartimento di Scienze e Tecnologie Agrarie, Alimentari, Ambientali e Forestali (DAGRI), University of Florence, Viale delle idee 30, 50019 Florence, Italy; 2 A.R.E.A. Foundation, via Tavoleria 28, 56125 Pisa, Italy; 3 Department of Biology, Università di Pisa, via L. Ghini 13, 56126 Pisa, Italy; 4 Institute of Life Sciences, Scuola Superiore Sant’Anna, Piazza Martiri della Libertà 33, 56127 Pisa, Italy; 5 Present address: Donald Danforth Plant Science Center, 975 North Warson Road, Saint Louis, MO 63132 USA; 6 Faculty of Biosciences and Technologies for Agriculture Food and Environment, University of Teramo, Via R. Balzarini 1, 64100 Teramo, Italy; 7 Proteomics and Mass Spectrometry Laboratory, I.S.P.A.A.M., National Research Council, 80147 Napoli, Italy; 8 Chromaleont Srl, c/o Department of Chemical, Biological, Pharmaceutical and Environmental Sciences Polo Annunziata, University of Messina, viale Annunziata, 98168 Messina, Italy; 9 Department of Chemical, Biological, Pharmaceutical and Environmental Sciences, Polo Annunziata, University of Messina, viale Annunziata, 98168 Messina, Italy; 10 National Research Council of Italy, Institute for Sustainable Plant Protection (CNR-IPSP), Viale P.A. Mattioli 25, 10125 Torino, Italy; 11 Division of Genetics and Cell Biology, IRCCS-Ospedale San Raffaele, Milan, Italy

**Keywords:** *Tuber magnatum* Pico, Sulfur compounds, Environment, Volatile organic compounds, Integrated approach

## Abstract

**Background:**

Truffles are symbiotic fungi that develop underground in association with plant roots, forming ectomycorrhizae. They are primarily known for the organoleptic qualities of their hypogeous fruiting bodies. Primarily, *Tuber magnatum* Pico is a greatly appreciated truffle species mainly distributed in Italy and Balkans. Its price and features are mostly depending on its geographical origin. However, the genetic variation within *T. magnatum* has been only partially investigated as well as its adaptation to several environments.

**Results:**

Here, we applied an integrated omic strategy to *T. magnatum* fruiting bodies collected during several seasons from three different areas located in the North, Center and South of Italy, with the aim to distinguish them according to molecular and biochemical traits and to verify the impact of several environments on these properties. With the proteomic approach based on two-dimensional electrophoresis (2-DE) followed by mass spectrometry, we were able to identify proteins specifically linked to the sample origin. We further associated the proteomic results to an RNA-seq profiling, which confirmed the possibility to differentiate samples according to their source and provided a basis for the detailed analysis of genes involved in sulfur metabolism. Finally, geographical specificities were associated with the set of volatile compounds produced by the fruiting bodies, as quantitatively and qualitatively determined through proton transfer reaction-mass spectrometry (PTR-MS) and gas-chromatography-mass spectrometry (GC-MS). In particular, a partial least squares-discriminant analysis (PLS-DA) model built from the latter data was able to return high confidence predictions of sample source.

**Conclusions:**

Results provide a characterization of white fruiting bodies by a wide range of different molecules, suggesting the role for specific compounds in the responses and adaptation to distinct environments.

## Background

The ectomycorrhizal fungus *Tuber magnatum* Pico is one of the best-known species belonging to the genus *Tuber*, which includes between 180 and 220 species [1]. *T. magnatum* is characterized as “whitish truffles”, fruiting bodies with white-colored gleba that are also produced by other *Tuber* species within the Puberulum group sensu *lato* [2]. Despite some valuable truffle species being amenable to cultivation, such as *Tuber melanosporum, Tuber borchii, Tuber aestivum* [3, 4] and *Tuber formosanum* [5], many attempts performed since 1984 to cultivate *T. magnatum* [6] have been unsuccessful. Due to the scarcity of samples harvested in the natural environment, the annual production does not cover the high demand for *T. magnatum* truffles, whose prices are ranging from 300 to 400 € hg^− 1^ [6, 7]. Different retail prices are applied to fruiting bodies depending on their harvesting place, considering that the geographical distribution of the species extends from Italy to East Europe (Croatia, Slovenia, and Hungary), including Greece [8], South of France [9] and Switzerland [10]. One distinct feature of the white truffle, as well as of fruiting bodies of different *Tuber* species, is in fact the highly different degree of appreciation by the consumers, which is related to the different aroma and flavor specificities due to truffle growth environment. Development of truffle fruiting bodies is, indeed, known to be influenced by a range of environmental variables, spanning from the host plant and the complexity of forest vegetation [11] to soil characteristics [12], climatic conditions [13] and the composition of soil bacterial communities [14]. This observation urged scientists to find reliable methods to discriminate among truffle accessions belonging to the same species.

The genetic variation within *T. magnatum* truffles was already investigated, evaluating intraspecific polymorphisms by simple sequence repeat (SSR) markers [15, 16]; eight loci showing polymorphic amplification were considered useful to assess population dynamics. Notably, an SSR-based analysis of *T. magnatum* truffles has revealed, for the first time, the presence of genetic and phylogeographic structures in natural populations of this *Tuber* species [16]. In fact, genetic studies have shown that both the Italian North-westernmost and the Southernmost populations are genetically different from all the other communities collected all over the species distributional range. Moreover, Mello and coworkers were able to demonstrate the existence of genetic diversity within Italian populations of *T. magnatum* using SCAR markers as a tool to identify single-nucleotide polymorphisms (SNPs) [17], thereby defining three different truffle haplotypes. These results were enlarged to *T. melanosporum* [18], where polymorphic SSRs suggested that this truffle is a species with relevant intraspecific diversity. An extensive SNPs analysis was also conducted on seven populations of *T. melanosporum* from Italy, France, and Spain, which led to the identification of more than 400.000 SNPs able to differentiate the analyzed samples [19].

On the other hand, Vita et al. [20] reported the existence of reproducible quantitative differences in the protein patterns of white truffle (*T. magnatum*) fruiting bodies coming from different Italian areas, suggesting that proteomic characterization might be a promising diagnostic method for origin attribution. In 2004 early work on *T. borchii* fruiting bodies [21] faced the issue of the limited sequence information available for truffles. However, protein identification in *Tuber* species has been greatly facilitated by the release of the complete sequence of the haploid genome of the Périgord black truffle *T. melanosporum* [22]. Moreover, sequencing of the *T. magnatum, T. aestivum* [23] and *T. borchii* [24] genomes have been very recently completed by international consortiums.

Recently, the profile of volatile compounds emitted by the fruiting bodies has been taken into consideration as an additional biomarker for truffle intraspecific classification. Biotic and abiotic factors have been shown to influence truffle aroma, including the nutritional content and the identity of the host tree [25]. In addition, many authors [26–29] have suggested that the aroma might vary according to the geographical origin of truffles of the same species. For instance, key volatiles were analyzed in the widely distributed species *T. aestivum* var. uncinatum, finding that the production of eight-carbon-containing compounds, which account for most of the aroma variability in this species, is likely to be under genetic control [30]. Experimental evidence on aroma-related proteins in truffle is currently limited to nine polypeptides from a *T. melanosporum* proteome (reconstructed through a combined polyacrylamide gel electrophoresis (1D PAGE) and high-accuracy liquid chromatography tandem-mass spectrometry approach coupled with bioinformatics analysis), which were found enrolled in the synthesis of volatiles in prior biochemical studies [31].

Neither individual method deployed so far could be considered as a definitive diagnostic method, for the sake of the attribution of truffle origin. A proteomic comparison of *T. magnatum* samples was equally insufficient to identify their collection locations [20]. It is worth note that truffle fruiting bodies harbor a diverse but poorly understood microbial community of bacteria, yeasts, and filamentous fungi [32], which might have an influence on the metabolism of the ascomata, rendering more difficult the comprehension of the factors influencing the fruiting bodies formation and functioning.

Here we provide an in-depth characterization of the *T. magnatum* fruiting bodies, collected in regions subjected to different environmental conditions, through parallel high-throughput approaches. An expanded proteomic assessment, as compared to previous experiments [20], was coupled to the examination of both transcriptome and volatome profiles (data from RNA-seq and VOC emission measurements, respectively) of samples harvested in three different Italian areas (Piedmont, Tuscany, Molise). The sampling strategy was planned to collect them in different years homogenously (e.g. distance from the plants), to avoid as much as possible the variability inside a collection site. On the basis of these global analyses, specific biochemical pathways involved in the biosynthesis of *T. magnatum* volatiles (e.g. sulfur compounds) were examined more closely through qPCR and compared to the data from Murat et al. [23]. The obtained combined datasets provide novel information to better understand the metabolic variations of *T. magnatum* to changing environmental conditions, which is at present underexplored, and suggest new putative tools that may be used in the next future for the geographical identification of white truffles.

## Results

### Identification of reproducible protein markers of white truffle origin via proteome profiling

**Fig. 1:**
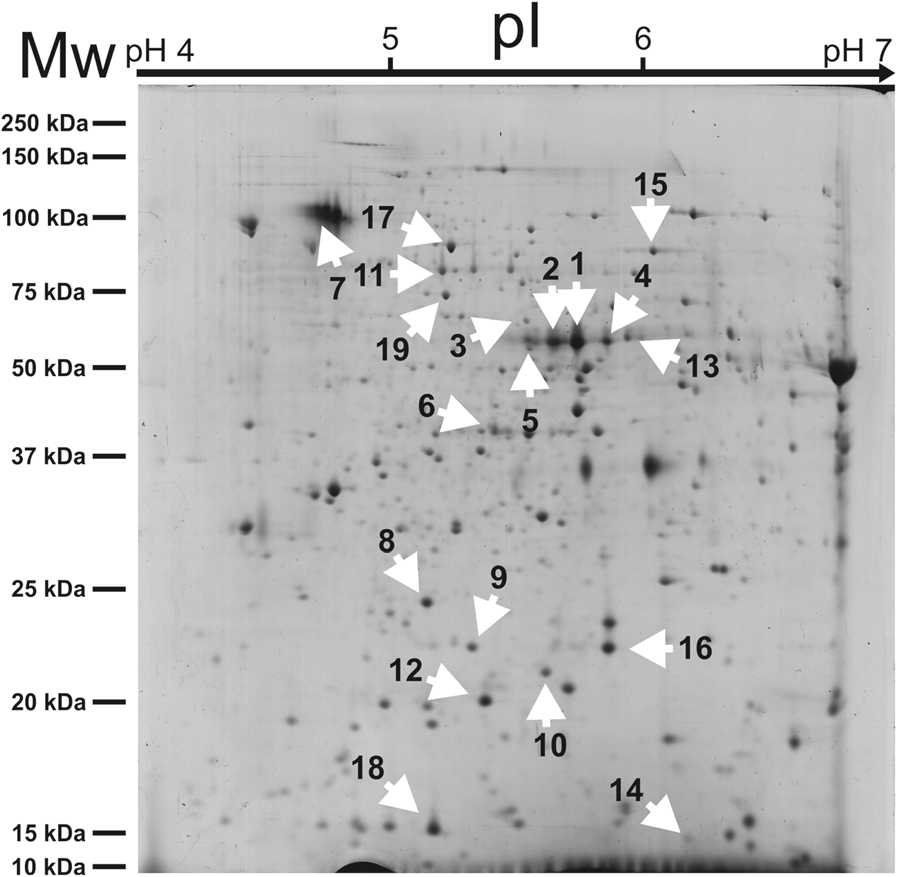
Representative 2-DE gel obtained from *T. magnatum* Pico mature fruiting body. Separation of total proteins from Isernia (IS) sample (1 mg of protein extract) stained with Coomassie G-250. The ranges of the first (above) and second dimension electrophoresis (left) are shown. White arrows indicate 19 spots that were selected after bioinformatic analysis of the global set of 2-DE gels produced

**Fig. 2:**
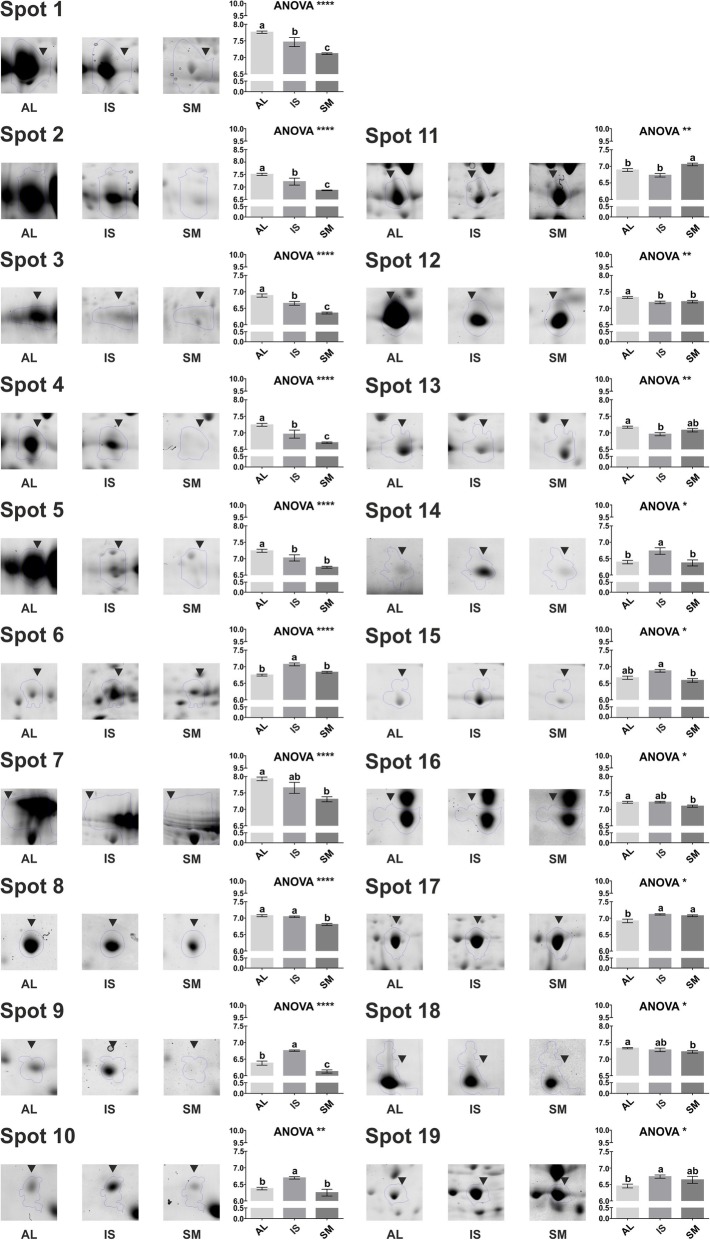
Normalized intensity levels of the spots selected for MS analysis. The relative amount of signal for each spot is expressed as a log10 normalized volume (spot optical density). Values are means ± SEM (*n* = 16, AL, SM; *n* = 12, IS). Statistical significance was evaluated by one-way ANOVA analyses, followed by Tukey HSD test (see Additional file 1: Table S1 for a summary of the test). Letters mark statistically significant treatments. Data are reported as *p*-values (*, 0.01 < *P* ≤ 0.05; **, 0.001 < *P* ≤ 0.01; ***, 0.0001 < *P* ≤ 0.001; ****, *P* ≤ 0.0001). AL, Alba; IS, Isernia; SM, San Miniato

*T. magnatum* fruiting bodies were harvested from different Italian areas (North: Alba, AL; Center: San Miniato, SM; and South: Isernia, IS) during 4 years of study (three for IS, Table 1). As first approach to assess their diversity, a comparison of their individual proteomes was undertaken. Proteins were extracted, resolved by 2-DE, and subsequently analyzed according to protein spot number, density, and size. More than 600 reproducible spots were detected in each gel (Fig. 1). Bioinformatic comparison of the protein patterns associated to the various samples led to the selection of 19 differential spots (Fig. 2), automatically ranked according to their *p*-value (< 0.05) and fold change (< 0.7, downregulated; > 1.3, upregulated), which displayed significant quantitative differences among samples. Analysis of variance, i.e. one-way ANOVA, and Tukey HSD post-test were used to evaluate the statistical significance of sample comparisons (see Additional file 1: Table S1). Most of the spots (1, 2, 3, 4, 5, 6, 7, 8, 12, 13, 16, 18) displayed higher medium average intensity in Alba samples, whilst spot 6, 9, 10, 14, 15, 17 and 19 were more abundant in Isernia samples; finally, only spot 11 and 17 were found as over-represented in San Miniato samples (Fig. 2). Given their elevated statistical significance, spots 1–9 appeared as the best indicators of different sample origin. In particular, seven of them (1, 2, 3, 4, 5, 7, 8) were associated with the highest significance in the comparison between Alba and San Miniato, while spot 6 and 9 showed top significance in the comparison Alba vs Isernia (AL vs IS) and Isernia vs San Miniato (IS vs SM), respectively (see Additional file 1: Table S1).

**Table 1:** Sampling site, mycorrhiza, and analyses performed. All fruiting bodies reached stage 5 of maturation [20], as described in Methods section

	Site (Province)	Region	Mycorrhiza	Years of analysis	Period	Sample names^a^	Total number of samples^b^
Proteomics	Alba (CN)	Piedmont	Poplar (*Populus alba*)	4	2012–2015	AL 2012 AL 2013 AL 2014 AL 2015	24
	Isernia (IS)	Molise	Poplar (*Populus alba*)	3	2013–2015	IS 2013 IS 2014 IS 2015	18
	San Miniato (PI)	Tuscany	n. s. (Wood)	4	2012–2015	SM 2012 SM 2013 SM 2014 SM 2015	24
RNAseq / qPCR	Alba (CN)	Piedmont	Poplar (*Populus alba*)	2	2014–2015	AL 2014 AL 2015	12
	Isernia (IS)	Molise	Poplar (*Populus alba*)	2	2014–2015	IS 2014 IS 2015	12
	San Miniato (PI)	Tuscany	n. s. (Wood)	2	2014–2015	SM 2014 SM 2015	12
PTR-ToF-MS analysis	Alba (CN)	Piedmont	Poplar (*Populus alba*)	4	2014–2017	AL 2014 AL 2015 AL 2016 AL 2017	27
	Isernia (IS)	Molise	Poplar (*Populus alba*)	4	2014–2017	IS 2014 IS 2015 IS 2016 IS 2017	24
	San Miniato (PI)	Tuscany	n. s. (Wood)	4	2014–2017	SM 2014 SM 2015 SM 2016 SM 2017	41
GC-MS analysis	Alba (CN)	Piedmont	Poplar (*Populus alba*)	4	2014–2017	AL 2014 AL 2015 AL 2016 AL 2017	23
	Isernia (IS)	Molise	Poplar (*Populus alba*)	4	2014–2017	IS 2014 IS 2015 IS 2016 IS 2017	18
	San Miniato (PI)	Tuscany	n. s. (Wood)	4	2014–2017	SM 2014 SM 2015 SM 2016 SM 2017	23

^a^ As reported in the figure and in the main text

^b^ The total number of analyzed samples during different years. Six independent biological replicates were analyzed for each sample over years for proteomic and molecular analysis. Five replicates were analyzed for VOCs analysis (GC-MS and PTR-ToF) for each accession during the first 2 years (2014–2015), whereas a variable number of samples (from four to fifteen) were analyzed during the remaining 2 years (2016–2017)

**Fig. 3:**
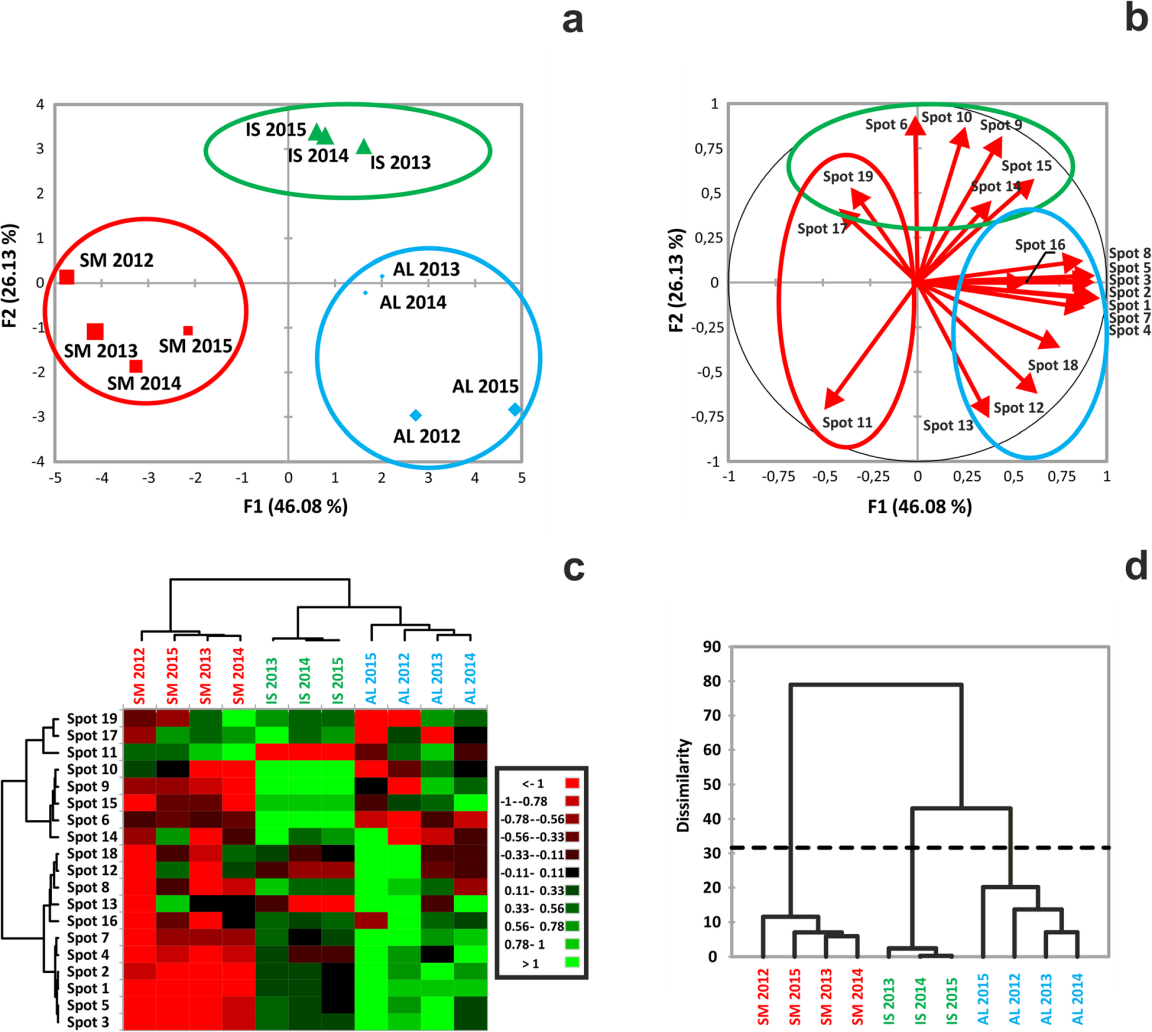
Result of variance analysis performed on proteomic data. **a** Individual sample map related to principal component analysis (PCA) of spot normalized intensities related to 19 spots. Sample names indicate location (IS = Isernia; AL = Alba; SM = San Miniato) and year of sample collection. Data reported represents an average value for each year of analysis. F1 = first dimension, F2 = second dimension. Total inertia (i.e., total variance) included by the first two dimensions of PCA accounted for 72.21% of the variance. **b** Correlation circle (variables factor map) related to the contribution of each variable (spot) in the distribution of the observations (samples). The length and the direction of the vectors are directly correlated to their significance. The angle between two vectors (α) defines the correlation of the associated variables: Positive correlation is present if 0 < α < 90°, while the correlation is negative if 90 < α < − 180°. No linear dependence exists if α = 90°. **c** Heat map based on quantitative data related to normalized spot intensities, whose discrete color scale is shown in the box. Green indicates over-representation, red down- representation. **d** Results of aggregative hierarchical clustering (AHC) analysis performed on spot data. C1-C3, sample distribution classes, based on their dissimilarity coefficient. The dotted line represents the degree of truncation of the dendrogram, used for creating classes and automatically chosen by the entropy level. Sample names correspond to those reported in Table 1

The relatedness of the samples was evaluated by a principal component analysis (PCA) applied to the intensity values of the selected 2-DE spots. Two principal components were found to account for 46.08% (F1) and 26.13% (F2) of the total variance (Fig. 3a). Remarkably, within the multidimensional space of the PCA, the samples grouped by geographic origin, forming three distinct clusters corresponding to the three collection areas. As a way to visualize the impact of each spot to sample differentiation, a variables factor map was generated that highlighted a high degree of significance (vector length above 50% of the radius) for all selected spots (Fig. 3b); the smallest contribution was calculated for spot 14, 16, 17 and 19. The same factor map can also display the relationships among variables (spots); here, we could observe that each spot was associated with both positive and negative correlations, with the exception of spot 11, which did not develop positive correlations (Fig. 3b).

Spot intensities were used to cluster either the spot themselves (Fig. 3c, left-side dendrogram) or the different samples (Fig. 3c, top-side dendrogram). The analysis returned four spot clusters with distinct quantitative profiles. Spots belonging to cluster 1 (no. 11, 17, 19) were over-represented in SM samples, spots of cluster 2 (no. 6, 9, 10, 14, 15) in IS and those of cluster 4 (no. 1, 2, 3, 4, 5, 7) in AL samples, while down-representation was prevalent in the other combinations. Moreover, sample clustering, in agreement with the PCA, confirmed that the selected spots were able to specify the source of the specimens. Similarly, hierarchical clustering of the samples identified the presence of three groups, broken down by their geographical origin (Fig. 3d). Among them, the one clustering SM samples displayed the highest degree of differentiation, according to the dissimilarity coefficient.

All 19 candidate spots retrieved by the bioinformatic analysis of the 2-DE gels were subjected to mass spectrometry, leading to the identification of 52 proteins in total (see Additional file 2: Table S2). Multiple identifications were obtained for most of the selected spots, with the exception of no. 9, 12, 15, 16, 18; subsequently, proteins were sorted in each spot by their respective emPAI values. We recovered a large number of uncharacterized proteins, whose biological function could only be inferred by sequence similarity (see Additional file 3: Table S3). On the other hand, we were able to identify 32 proteins through BLAST analysis (see Additional file 3:Table S3). At a first survey, they appeared to be primarily associated with calcium metabolism (e.g., a putative calcium homeostasis protein regucalcin), glycolysis (e.g., fructose-bisphosphate aldolase), or amino acid metabolic processes (e.g., cystathionine gamma-lyase and the pyridoxine biosynthesis protein pdx1).

**Fig. 4:**
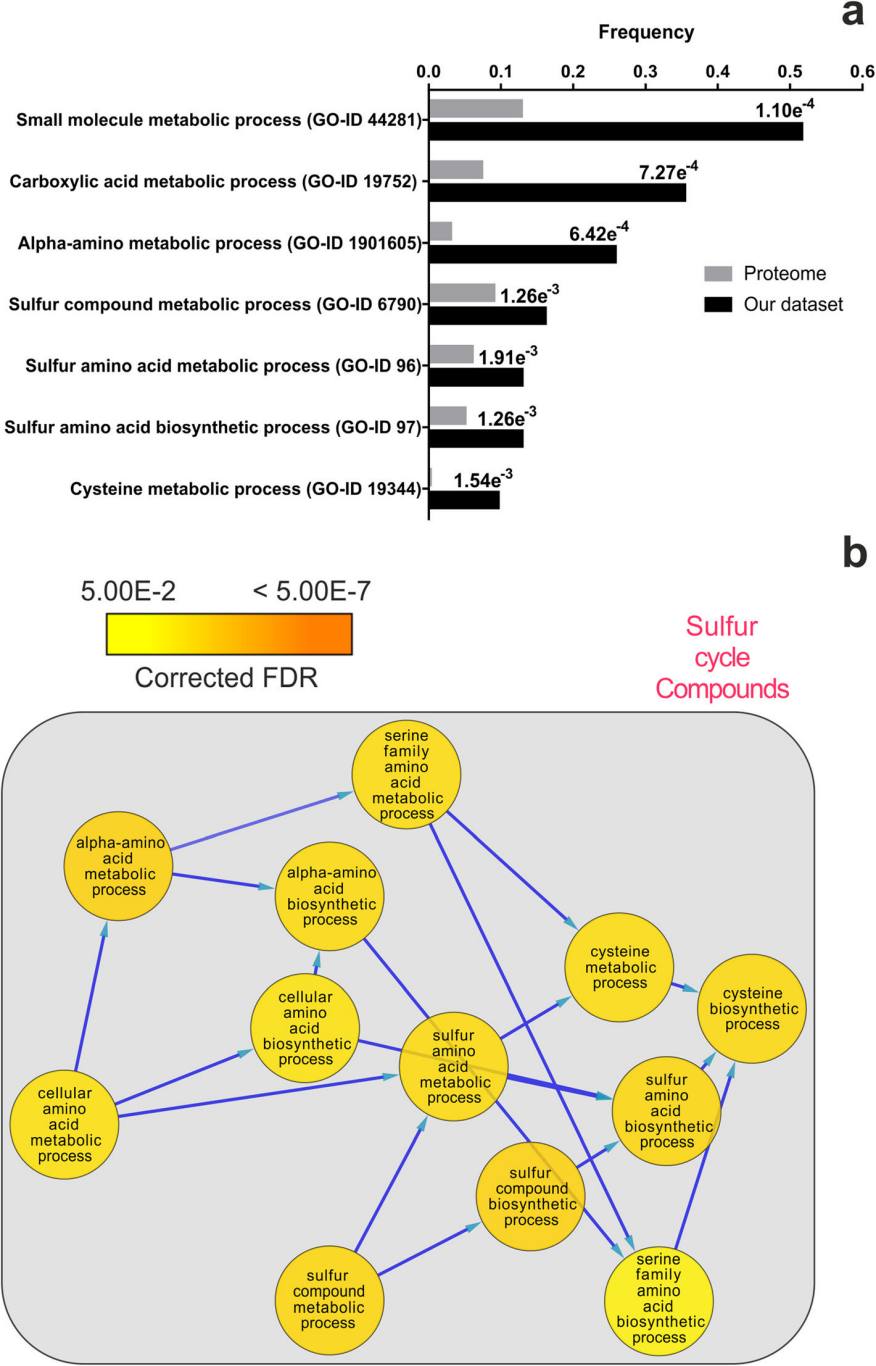
Functional categorization of the 52 proteins identified upon MS analysis of the discriminative protein spots from 2-DE. **a** Overview of significantly enriched biological process-associated Gene Ontology categories, based on *T. melanosporum* annotation of the MS dataset proteins. Frequency data refers to cluster frequency ratio (black bars) and total frequency ratio (grey bars). Specifically, black bars represent the number of annotated proteins from the MS dataset associated with each GO term divided by the total number of identified and annotated proteins of the MS dataset, while gray bars represent the number of proteins in the *T. melanosporum* proteome reference set associated with each GO term divided by the total number of annotated proteins in the proteome reference set. The corrected FDR after statistical analysis is reported for each GO term. **b** Graphical description of the sulfur compound GO terms contained in the categories listed in panel A. Nodes, represented by circles, are shaded according to a p-value color coding obtained by statistical analysis. The range of the color scale varies from yellow (down-represented) to orange (over-represented)

To gain insights into the functional categorization of the proteins identified by MS, namely to group them based on their biological properties, we subjected the dataset of differentially expressed proteins to a Gene Ontology (GO) enrichment analysis. Among the over- or down-represented biological process-related categories we found, terms associated to small molecule, carboxylic acid and alpha-amino metabolism over-represented with the highest statistical significance (Fig. 4a). More intriguingly, various categories related to sulfur cycle compounds were significantly over-represented (Fig. 4b). Indeed, five sulfur-related proteins were retrieved by our differential analysis (see Additional file 4: Table S4). Cystathionine gamma-lyase (CTH) and S-adenosylmethionine synthase (SAM) came from spots over-represented in AL, cobalamin-independent Met synthase (MetE) from a spot over-represented in IS, adenosylhomocysteinase (AHCY) and peptide methionine sulfoxide reductase (MsrA) from spots down-represented in SM (Fig. 2). These observations hint at a differential regulation of sulfur metabolism as a determinant of proteome diversification in the fruiting bodies of white truffles from different areas.

### Transcriptome changes are associated with white truffle source

**Fig. 5:**
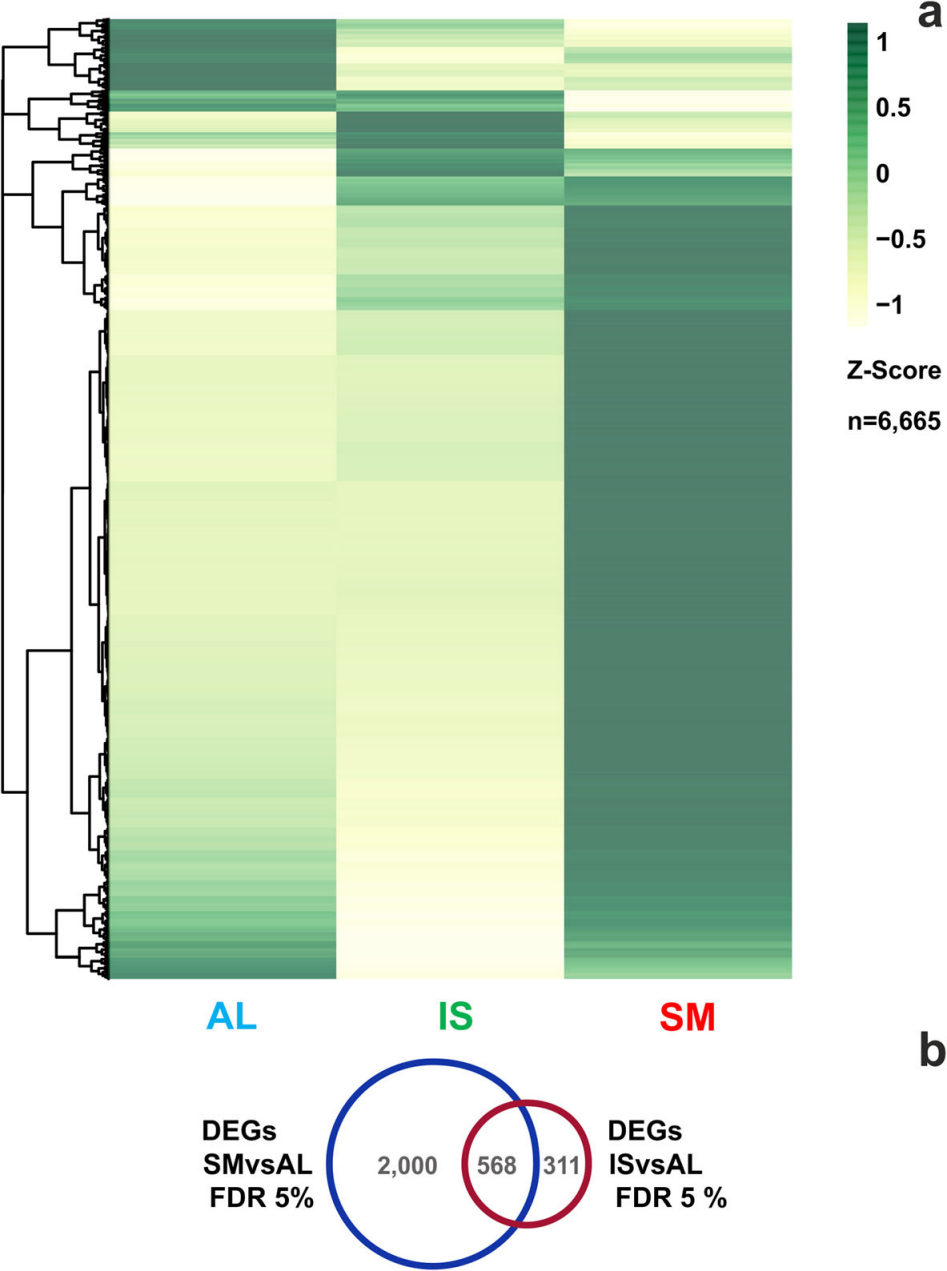
RNA-seq analysis of *T. magnatum* fruiting bodies of different geographical origin. **a** Heat map representing the differential expression profiles of *T. magnatum* genes among the three sampling locations (AL, Alba, IS, Isernia, SM, San Miniato). Rows (genes) and columns (locations) were hierarchically clustered with the Euclidean method. Gene expression is displayed as Z-scores, row-normalized expression values calculated as (observed TPM – row mean TPM) / row TPM standard deviation. TPM, transcripts per million. Yellow indicates expression values lower than row means, dark green represents values higher than row means. **b** Venn diagram of differentially expressed transcripts (FDR < 5%). Alba sample was set as the internal standard for sample comparisons

To better understand the observed proteome dynamics and assess how the protein profiles compared with changes at the level of gene expression, we carried out a whole transcriptome sequencing. *T. magnatum* samples were collected in the three different locations and the time points associated with the sampling campaign were considered as biological replicates. When this experimental work was started, the genome of the *T. magnatum* was not known, therefore we decided to analyze the whole transcriptome dataset using the de novo reference assembly of *T. magnatum* provided by Vita et al. [33], containing 12,367 transcripts reunited in 6723 high-confidence protein-coding genes. This strategy was adopted considering that *T. magnatum* RNA-seq reads cannot be mapped against the closest truffle species *T. melanosporum* reference genome (1% of mapped reads), as reported by Vita et al. [33]. Processed clean reads were mapped in a quasi mapping mode using the Salmon pipeline (see Methods section) against the 12,367 transcripts, with an average mapping rate of 56.64% (see Additional file 5:Table S5). We also mapped the reads against the entire transcriptome assembly generated by Vita et al. [33], containing ~ 23 K transcripts, to cross-validate the overall mapping rate our RNA-seq experiment, and found an alignment rate of 70.48% (data not shown). As reported in Supporting Information (see Additional file 6: Figure S1), Euclidean metric showed that the three samples clustered apart according to their geographical origin (SM, IL, AL), while showing strong correlation among the biological replicates. We identified differentially expressed genes (DEGs) according to sampling location and subjected them to two pairwise comparisons: SM vs AL and IS vs AL, where the samples from Alba were used as control. Major differences (see Additional file 7: Figure S2) emerged in the gene expression profile of San Miniato fruiting bodies, with 2568 statistically significant DEGs (FDR, false discovery rate = 5%), against 879 from the comparison IS vs AL. Consistently, sample separation based on Euclidean distances clearly isolated SM from AL and IS (see Additional file 6: Figure S1) and associated it to a markedly distinct gene expression profile (Fig. 5a). Moreover, the Venn diagram (Fig. 5b) showed little overlap of DEGs between the two pairwise comparisons, highlighting the occurrence of specific transcriptional responses determined by the geographical location of the samples. Data related to the 100 most statistically significant transcripts for each of the two comparisons made are shown in Supporting Information (see Additional file 8: Table S6 and Additional file 9: Table S7).

Further information on the results of sample comparisons could be obtained by a Shannon entropy distribution plot (see Additional file 10: Figure S3 and Additional file 11: Data file S1), which provides an estimate of the sample specificity of gene expression across samples. The analysis returned 252 genes with high specificity (SH > 0.6) among the three analyzed samples, which constitute a set of genes among which candidate markers might be selected in the future.

### Transcriptional regulation of the sulfur compound pathway in white truffle fruiting bodies

**Fig. 6:**
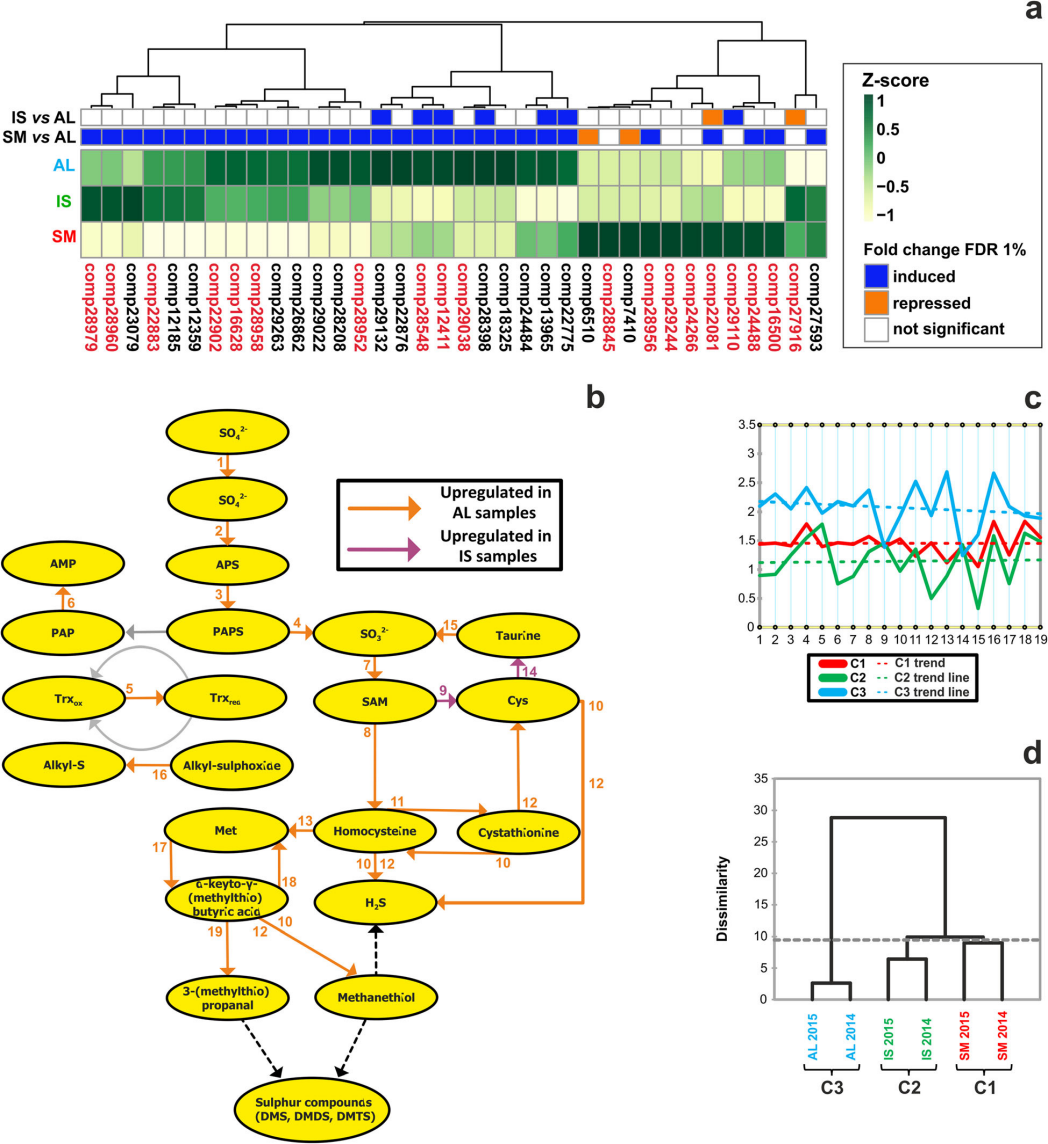
Transcriptional regulation of sulfur VOC pathway genes in white truffle fruiting bodies. **a** Hierarchical clustering (Euclidean method) of genes related to sulfur metabolism. The heat map displays the Z-score of the identified transcripts, as measured in the RNA-seq analysis. On the top, significantly regulated genes across the pairwise comparisons (FDR < 1%) are shown in color, while non significant values (FDR > 1%) are shown as white cells. Induction or repression refer to the AL sample (internal standard in all pairwise comparisons). Red marked genes were further analyzed through qPCR. Additional information on the selected transcripts is reported in Supporting Information (see Additional file 12: Table S8). **b** Schematics of the sulfur VOC metabolic pathway derived from Martin et al. [22]. Numbers, indicating the enzyme catalyzing the specific reactions associated to each step, correspond to those listed in Supporting Information (see Additional file 13: Table S9). Coloured arrows mark those steps whose coding genes were analyzed by qPCR (see Additional file 14: Figure S4); conversely, grey arrows indicate not analyzed genes and the outcome of the measurements is visulized through different arrow colors, where orange represents genes up-regulated in AL samples and green those up-regulated in IS samples. **c** Relative profile plot of expression of the selected 19 genes across SM (red line), IS (green line) and AL samples (blue line). Median expression values are plotted and respective trend lines are shown. **d** Dendrogram representation of aggregative hierarchical clustering performed on the qPCR dataset

Our samples were shown to be distinguished by proteins involved in the metabolism of sulfur-containing organic molecules (Fig. 4a). Therefore, we decided to assess whether the associated pathways might undergo differential transcriptional regulation, looking for sample-specific gene expression patterns in the biosynthesis or utilization of those compounds. In first place, we decided to filter our global transcript profiling data for those transcripts associated to sulfur metabolism, according to Gene Ontology (see Additional file 12: Table S8). DEGs belonging to this selection appeared to group into three well-defined clusters (Fig. 6a). In the first and second ones, the AL sample was upregulated, while in the third cluster, where nonetheless most of the values were non-significant (FDR > 1%), higher expression was recorded in SM. Overall, the analysis indicated that sulfur pathway genes could indeed respond to differences in truffle growth environments.

To confirm the observed regulation, we measured transcripts corresponding to genes involved in sulfur pathway [23] by quantitative PCR (see Additional file 13: Table S9). In detail, we have tested 19 genes involved in the metabolism of sulfurated amino acids (methionine and cysteine) (Fig. 6b), as components of a route leading to the production of many sulfur organic compounds [22, 23].

Statistically significant differences were found when the overall dataset of qPCR gene expression was evaluated through two-way ANOVA, considering sample type and individual genes as variables (see Additional file 14: Figure S4). Only *cysteine ​​synthase* (gene 9) and *cysteine ​​dioxygenase* (gene 14) were found to be almost unaffected by sample origin. Instead, the majority of the genes were found as up-regulated in AL samples in at least 1 year of collection; in particular, with the exception of *thioredoxin reductase* (gene 5), *taurine dioxygenase* (gene 15), *BCAT1* (gene 17) and the *aromatic amino acid aminotransferase* (gene 18), the up-regulation in AL was observed during both years. The most pronounced differences between AL and the other samples were observed in the case of *cystathionine beta synthase* (gene 11) and *cobalamin-independent methionine synthase* (gene 13). Overall, the average expression values of the 19 selected genes was higher in Alba when compared with other samples (Fig. 6c); thus, this picture was consistent with the differential expression analysis of the RNA-seq data, where genes linked to sulfur metabolism turned out to be mostly upregulated in Alba samples (Fig. 6a). Aggregative hierarchical clustering of the qPCR dataset showed that, finally, sulfur pathway expression profiles successfully enabled sample discrimination according to their source, with Alba samples being the most differentiated and the other two accessions showing a higher degree of similarity (Fig. 6d).

### Identification of discriminative VOCs with two different analytical techniques

#### GC-MS results of VOC analysis

**Fig. 7:**
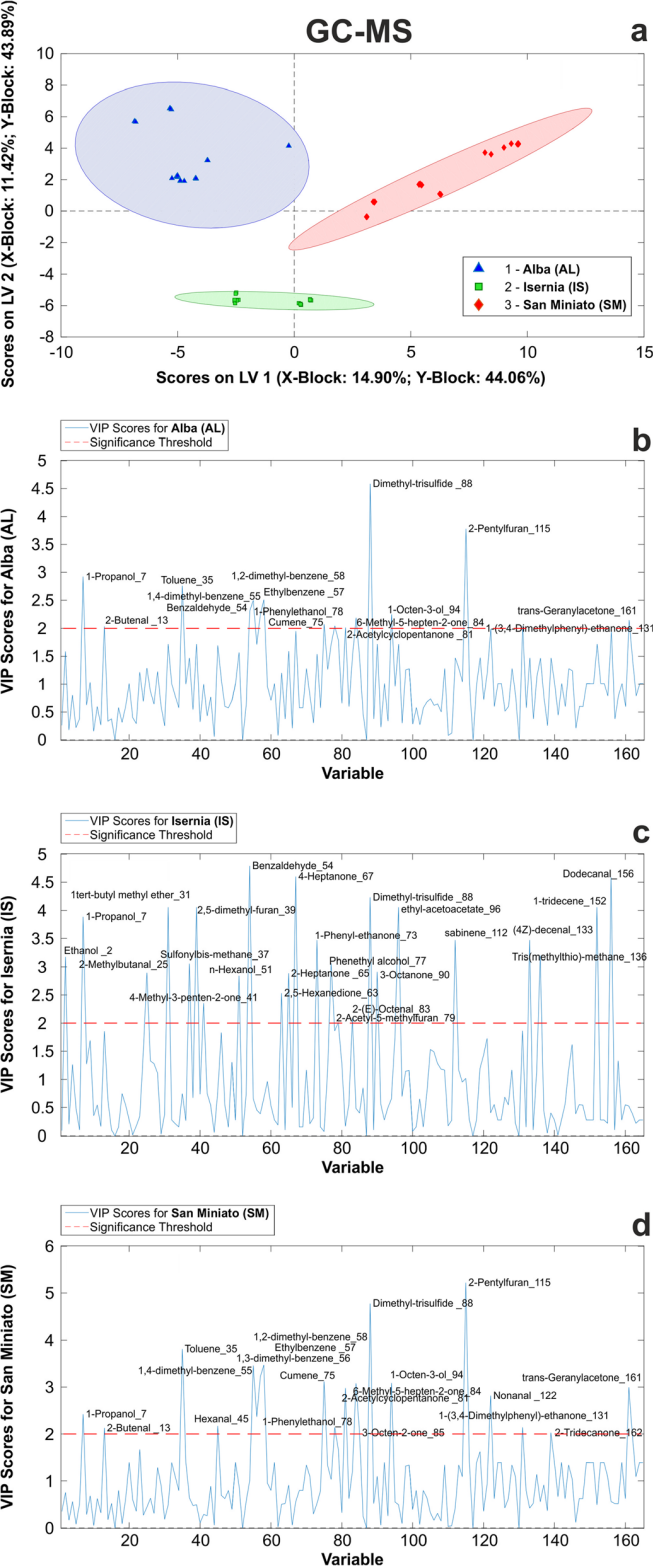
PLS-DA of VOCs anlyzed by GC-MS. **a** Score plots on the latent variables (LV1 and LV2) calculated from the PLS-DA model that was applied to AL (red squares), IS (green squares) and SM truffles (blue triangles). Each item represents a biological replicate, collected over the 4 years of sampling. VIP scores calculated for each VOC (x-axis, “variable”; progressive numbers correspond to those in Additional file 15: Table S10) in **b** Alba, **c** Isernia and **d** San Miniato samples. A significance threshold set at VIP = 2 is indicated

One main goal of our assessment was to build up a comprehensive picture of the changes in volatile molecule composition of fruiting bodies from different geographical accessions, with the aim to provide a quantitative basis that might be useful to understand how truffle aroma is influenced by the environment. We characterized a set of 165 volatile organic compounds (VOCs) accumulated by truffles of the three accessions by means of GC-MS chromatography (see Additional file 15: Table S10). We then used the data to build up a predictive classification model able to differentiate samples collected in different years. The partial least squares discriminant analysis (PLS-DA) supervised classification method applied led to the successful identification of the taxonomic category of the three samples (i.e. Alba, Isernia, San Miniato origin) (Fig. 7a). Results showed a perfect match of each sample to the right category (see Additional file 16: Table S11a). No wrong assignation was reported for these samples, as highlighted by results of confusion matrices. The number of latent variables (LVs) associated to the minimum error rate and concurrently to the minimum number of not assigned samples resulted in 2 LVs (see Additional file 16: Table S11a). The global quality of the model, evaluated by its performances indicators (see Additional file 16: Tables S11 a-c), resulted robust enough to discriminate the three *T. magnatum* samples in the model/validation data set, and in the independent test set. In fact, the two-component PLS-DA model successfully classified 100% of truffle samples into their taxonomic category in fitting, cross-validation (internal validation) and prediction (external validation) (see Additional file 16: Table S11b). Upon permutation test, applied to validate the model, we found it to be significant at 95% confidence level (see Additional file 16: Table S11c). Moreover, a variable importance in the projection (VIP) score was calculated from the PLS-DA model for every identified compound (variable) to summarize its contribution to the overall model. For each accession, we found compounds with significant (> 2.0) VIP scores (Fig. 7b-d). More importantly, we observed statistically significant variations in individual VIP scores across samples (see Additional file 17: Table S12), which highlighted the role of specific molecules in differentiating the three accessions under consideration. Dimethyl-trisulfide (*88*) and 1-propanol (*7*) represented the most significant compounds able to distinguish the three sample classes, although in distinct ways; the sulfur compound discriminated AL samples, where it was absent, whereas 1-propanol was not detected in the IS dataset.

All compounds selected as significant in at least one sample (VIP > 2) after PLS-DA were then grouped according to their chemical properties before the statistical analysis, to understand whether particular VOC classes could be considered prominent in sample diversification. VOCs selected by PLS-DA analysis never displayed the highest VIP values in AL samples, when compared to the other two accessions. Considering the IS and SM samples, on the other hand, we noticed the existence of trends linked to each chemical class. Concerning the sulfur-containing compounds, hydrocarbons, esters and terpenes (only one compound considered for each of the three), IS showed the most statistically significant values. The same applied to alcohols, where three of the four compounds had the highest significance in IS. Instead, 1-octen-3-ol (*94*) had a higher VIP score in SM. An intermediate situation was observed for aldehydes, ketones and those compounds classified as “others”, where IS and SM contributed with a comparable number of compounds with the highest significance. As to the aromatic compounds, the greatest number of statistically significant compounds belonged to the SM accession, as compared to IS; in particular, compounds such as 1–2 and 1–4 dimethylbenzene had a much higher level of significance in the samples coming from San Miniato.

#### PTR-ToF results of VOC analysis

We therefore subjected our samples to PTR-ToF-MS analysis, with the aim to improve the coverage of truffle molecular profiling with this state-of-the-art technique, which identified 65 compounds in the *m/z* range 0–130 (see Additional file 18: Table S13). Following the strategy described above, the data were then used to build up a PLS-DA model, and the global quality of the model was evaluated by its performances indicators (see Additional file 19: Tables S14 a-c). We found that the model successfully classified 97.4% of the samples into their correct class (geographical accession), both in fitting and cross-validation (internal validation), and 93.7% of them in prediction (external validation).

**Fig. 8:**
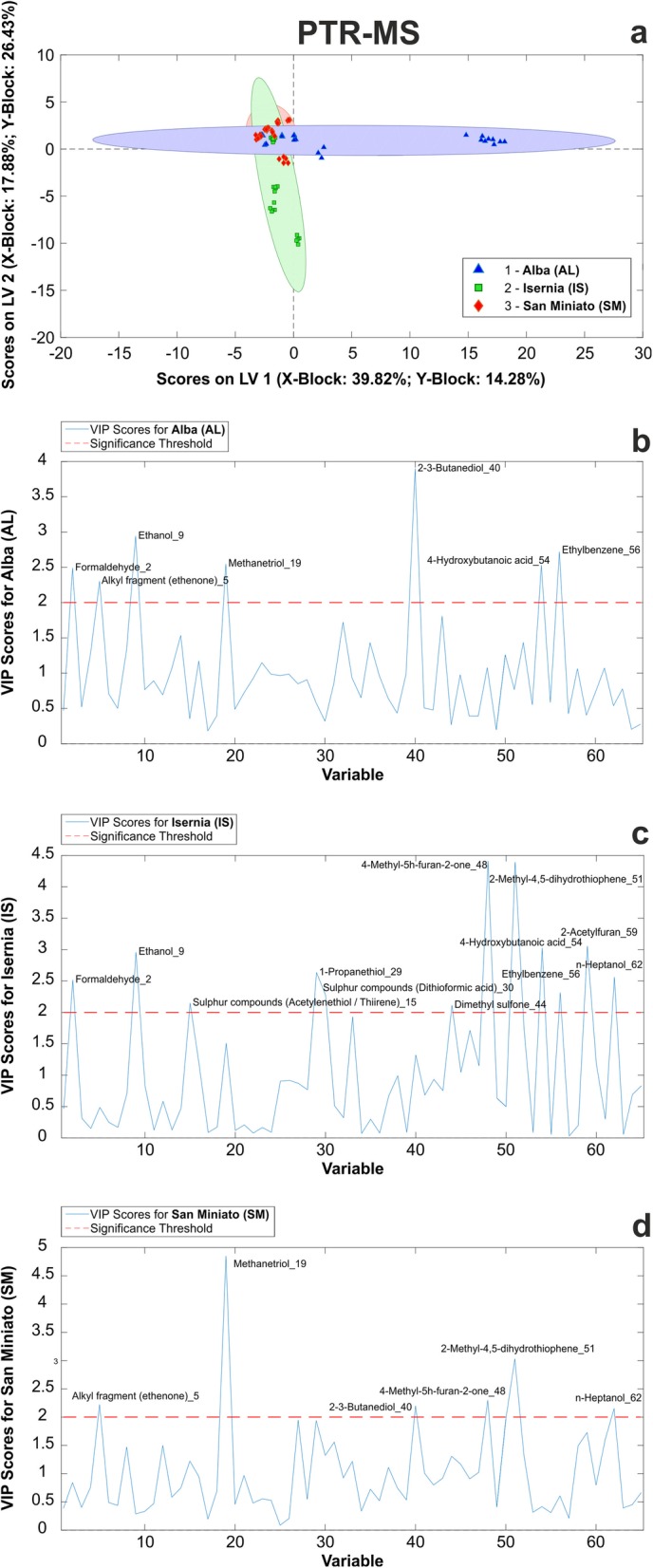
PLS-DA of VOCs anlyzed by PTR-ToF-MS. **a** Score plots (LV1, LV2) from statistical model for Alba (red points), Isernia (green points) and San Miniato (blue points) truffles. **b**, **c**, **d**, VIP scores for each class (Alba, Isernia, San Miniato)

In the prediction results, 15/16 samples were correctly assigned to the right class (see Additional file 19: Table S14b). Two optimal latent variables number were found to be associated with the minimum error rate and concurrently to the minimum number of not assigned samples (see Additional file 19: Table S14b). The permutation test indicated that the model was significant at 95% confidence level (see Additional file 19: Table S14b). The associated scores plot of the two-component PLS-DA model is shown in Fig. 8a. Also in this set of VOCs, we isolated compounds with significant VIP scores (VIP > 2.0) that may be able to differentiate the three considered classes (see Additional file 20: Table S15). However, samples resulted to be less differentiated in this model than in the one obtained from the GC-MS profiling (Fig. 8a).

Closer examination of the statistically significant VOCs found in each sample revealed 2–3-butanediol (*40*) as the most distinctive compound for AL samples (Fig. 8b), whereas 4-methyl-5 h-furan-2-one (*48*) and 2-methyl-4,5-dihydrothiophene (*51*) were the most significant ones for IS (Fig. 8c). Finally, methanetriol (*19*) and 2-methyl-4,5-dihydrothiophene (*51*) showed maximum VIP scores in SM samples (Fig. 8d). In particular, the latter compound was uniquely detected in SM samples, therefore representing the most discriminating VOC from this set.

#### PLS-DA model analysis

**Fig. 9:**
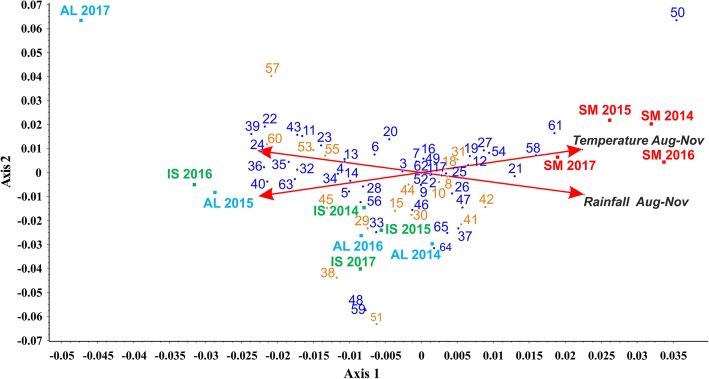
Triplot from CCoA (canonical correspondence analysis) of the VOCs data from PTR-ToF-MS. Sample biological replicates (red squares for SM, green for IS and light blue for AL; samples are plotted by their LC score) are showed along with the compounds identified through PTR-ToF-MS (numbered according to Additional file 18: Table S13; scored by their protonated *m/z*) and two environmental variables (red arrows). Yellow tags mark the volatile sulfur compounds (VCSs) from the other volatiles (blue tags)

Prompted to investigate the source of variability that the PLS-DA model could not capture (Fig. 8a), we hypothesized the occurrence of environmental effects on the VOCs emission of our samples. To uncover them, we decided to apply a canonical correspondence analysis (CCoA, a widely used approach for the exploration of ecological data [34]) to the whole data set of VOCs compound from PTR-ToF-MS, and to two environmental variables involved in the development of mature ascocarps: average temperatures and precipitations (August to November, see Additional file 21: Table S16) collected from three different stations (Alba, Isernia, and San Miniato) during a four-year period (2014–2017). By this analysis, we observed that SM samples were positively correlated with variable “Temperature” (Aug-Nov), whereas AL and IS did not show any unambiguous trend (Fig. 9). As to the individual compounds, VOCs widely distributed among those positively and negatively influenced by the environmental variables. However, most sulfur compounds (yellow tags), i.e. 3-methylthio-propionaldehyde (*53*), 2-methylthioacetic acid (*55*) and 2-methyl-3-furanthiol (*60*), negatively correlated with the variable “Rainfall”, with only methanethial (*8*) being slightly positively correlated with it (Fig. 9). As to the other compounds, (2E)-butenal (*24*), 2-propenal (*13*) and alkyl fragment (*4*) were negatively affected by the variable “Rainfall”, while 1-Butene (*14*) negatively correlated with the “Temperature”. Finally, anisole (*58*) and alkyl fragment (*12*) were the most influenced compounds by the variable “Temperature”. Results of CCoA ordination showed how this analysis is able to extract axes that explain as much as possible of the total variance due to the constraining effect of the environmental variables, as highlighted by the role of sulfur compounds.

Remarkably, sulfur-containing volatile compounds (VSCs) represented nearly 30% of the selected VIPs and were statistically significant in IS samples (see Additional file 20: Table S15). These data are in partial agreement with those obtained by GC-MS, indicating that, along with other chemical compounds, specific VCSs could represent useful discriminative markers between the three accessions. Some of the VCSs were shown to be influenced by temperature and precipitations (Fig. 9), suggesting a possible correlation between the emission of VOCs and the environmental conditions during fruiting body development.

## Discussion

In recent years, studies on environmental adaptations of non-model species have increased, with molecular insights made possible by novel comprehensive techniques available for data analysis. Molecular markers that have been most widely used to distinguish the prized *Tuber* species include PCR-RFLP, species-specific primers, barcoding and phylogeny of the internal transcribed spacer (ITS), and the β-tubulin gene [17, 35–37]. These methods were very useful to detect genetic differences inside a species [38], offering also information about the geographical origin [16]. These previous papers, however, did not provide information on the differences related to changes in truffle metabolism, e.g. in relation to different environments. Recently, mass spectrometry (MS)-based profiling has been proposed as an alternative method to detect different species of truffles [35]. In detail, the species identification inner the *Tuber* genus was performed and results were compared to that from other experimental approaches, mainly those based on molecular methods such as the ITS-based analysis. As the authors stated [35], the analysis seems less laborious and time-consuming with respect to those based on classical molecular approaches e.g. ITS-based analysis, highlighting better performances in terms of the easiness of sample manipulation and the rapidity in getting final results. However, MS-based approaches generally require expensive instruments and well-trained personnel [39].

On the other hand, molecular biomarkers related to the organism phenotype are generally measurable indicators of a biologic status and they can vary as a result of environmental changes. In the last years, the development and the spread of -*omics* techniques have represented a valid approach to identify biomarkers associated with a specific steady-state. Proteomic techniques, for example, have been frequently used for the discovery of differentially expressed proteins, including biomarkers [40], as in wine to identify either the presence of fining agents or wine-specific proteins which are mainly present in the range of 20–30 kDa [41].

In the case of fungi belonging to *Tuber* genus, researches are improved by the recent publication of the genome sequence for several of them [23, 24]. Particularly, the recent paper on *T. magnatum* genome sequencing [23], in which the sequencing data of other species such as *T. aestivum* have also been reported, has contributed to increase the level of information for the species belonging to the *Tuber* genus, which were previously based on the genome of *T. melanosporum* [22] and T. *borchii* [24]. Transcriptome comparisons among the three species are expected to yield valuable insights on the quality determinants of fruiting bodies with high economic value as well as on ecological aspects, through the identification of genes involved in truffle development through its phenological phases and in its establishment of mutualistic symbioses. In this work, we generated a new integrated “omic” platform that coupled proteomics and transcriptomics to the analysis of VOCs, with the aim to evaluate the effects of environmental adaptation in *T. magnatum* fruiting bodies. This allowed us to draw a comprehensive picture of mature fruiting body metabolism.

However, truffle fruiting bodies also contain bacteria [42] that seem to be selected from the soil communities during the early stage of truffle formation [43]. In *T. borchii*, Splivallo et al. [42] have already demonstrated that thiophene volatiles characteristic of *T. borchii*-fruiting bodies were produced by the microbiome inhabiting truffle-fruiting bodies. The core microbiome of truffle-fruiting bodies, which is dominated by α-Proteobacteria, might be supplemented with additional species depending on the fungal species, the maturation stage or the environment [42]. Benucci and Bonito [32] reported the presence of a single Bradyrhizobium OTU as dominant within truffle species belonging to the genus *Tuber*, irrespective of geographic origin, but not in other truffle genera sampled. However, we cannot exclude that the differences observed between samples collected in different environments could be also related to a difference in these fruiting-bodies associated bacteria, which can at least in part reflecting the soil microbial community, that may influence transcriptomic, proteomic and volatilome profiles.

Indeed, each of the three approaches adopted brought to highlight reproducible differences among the samples under investigation. Proteomic tecniques were widely used for determining environmentally-induced changes in protein composition [44, 45]. Our proteomic pipeline retrieved spots that were conserved in every set of samples (i.e. specimens of identical source), thereby identifying those that were consistently regulated across subsequent seasons in dependence of sample geographical origin (Fig. 3 and Additional file 1: Table S1). The RNA-seq profiling revealed the extent of the transcriptional regulation among accessions, highlighting a higher differentiation between Alba and San Miniato samples than between Alba and Isernia (Fig. 5); these results well explain the advantages of using high-throughput techniques like RNA-seq, with the aim to detect changes as related to environmental adaption, according to their high resolution and sensitivity [46]. Finally, VOC profiling proved to be a valid approach for the discrimination of local differences, since a PLS-DA predictive model based on GC-MS classified the samples according to their origin with high statistical confidence, without being affected by the year of sampling.

Considering PTR-ToF data, CCoA was performed to evaluate the impact of environmental variables on VOC emission. Results showed a clear correlation trend certain degree among variables and one of the samples (SM), leading to highlight as temperature and rainfall influence the emission of individual VOCs, some of them containing sulfur.

For one class of putative discriminative markers, i.e. those related to sulfur compound metabolism, the outcome of the three diagnostic approaches showed a remarkable convergence. Several among the differentially expressed spots found through the 2-DE approach contained proteins involved in sulfur amino acid metabolic processes (see Additional file S4: Table S4) and the same metabolic pathway resulted to be regulated across transcriptome profiles in our accessions (Fig. 6 and Additional file 12: Table S8). Beyond constituting an essential nutrient for routine cellular functions in filamentous fungi [47], sulfur is incorporated in a number of volatile molecules (namely, VSCs) that, in combination with other classes of VOCs, determine truffle aroma [25, 27, 48–51]. Our study reinforces the evidence that VSCs constitute robust markers for the traceability of white truffles [26, 52–54]; this was mainly true in the case of the GC-MS analysis, where major determinants (e.g., dimethyl-trisulfide) of sample differentiation were found (Fig. 7 and Additional file 17: Table S12). Additionally, our study suggests proteins involved in VSC metabolism as “biomarkers” of white truffle origin.

The VSC-related proteins identified in our study (see Additional file 4: Table S4) are involved in different steps of methionine/cysteine metabolism and carry out different metabolic roles. CTH converts l-cystathionine to l-homocysteine, but is also able to produce methanethiol from methionine and H_2_S from cysteine in *Saccharomyces cerevisiae* [55, 56], where it might be responsible of the observed VSC production in the presence of methionine as a precursor [57]. Methionine sulfoxide reductases (MSRs) can promote the reduction of methionine sulfoxide in proteins back to methionine, a function that restores protein inactivated by Met oxidation, and seem to take part to cellular protection against oxidative damage [58]. The expression of MXR1, a particular MSR performing dimethyl trisulfide (DMSO) reduction to dimethyl sulfide (DMS) in brewing yeast is a major determinant for DMS concentration DMS in beer [59]. SAM catalyzes the synthesis of S-adenosylmethionine (AdoMet), a methyl donor for transmethylations and a propylamino donor in polyamine biosynthesis, from methionine and ATP [60], which takes part in cysteine/methionine biosynthesis and interconversion and plays a key role in the production of H_2_S [22]. The latter, in turn, is a precursor of several volatile compounds, including DMSO and DMS [61] that are main determinants of *T. magnatum* flavor. Furthermore, AHCY is involved in L-homocysteine synthesis from S-adenosyl-L-homocysteine [62], which acts as a competitive inhibitor of SAM-dependent transmethylation reactions [63].

Homocysteine might represent a limiting factor and a possible index of environmental adaptation in white truffle. It is a key intermediate of the conserved transsulfuration pathway, by which sulfur is organicated in filamentous fungi [47] and which comes into play in sulfur limiting conditions [47, 64, 65]. Two enzymes that make use of homocysteine as a substrate, cystathionine beta-synthase and MetE (cobalamin-independent Met synthase, [66]), were found regulated at the transcriptional level (see Additional file 14: Figure S4) and, in the case of MetE, also at the protein level (see Additional file 4: Table S4). Finally, although not supported by significant changes in the protein amount, the transcriptional regulation of *cysteine synthase* and *cysteine dioxygenase* in Isernia samples (see Additional file 14: Figure S4) might be indicative of specific adaptations to changes in nutrient conditions [67] and differential sulfur assimilation through the modulation of cysteine levels [68].

Data from Murat et al. [23] reported new genetic resources about eight different Pezizomycetes, including a complete picture of the enzymes involved in VOC production that were clustered into 4 pathway classes, i.e. sulfur metabolism, Ehrlich pathway, synthesis from fatty acids and synthesis of isoprenoids. These classes cover most of the reactions involved in VOCs production currently known, showing strong differences among the three analyzed *Tuber* species. In detail, most of the most up-regulated genes in all the three species belonged to the sulfur related classes and were involved in methionine uptake and biosynthesis as well as the homocysteine/methionine cycle. The third sub-class related to sulfur metabolism named S-VOC synthesis from methionine showed a different behavior reported of each *Tuber* species.

Going deeper in data analysis, we may observe as some of the highest upregulated genes in *T. aestivum* like Cystathionine gamma-lyase (CYS3), S-adenosyl-L-homocysteine hydrolase (SAH1), Cobalamin-independent methionine synthase (MET6), S-adenosylmethionine synthetase (SAM1) and ATP sulfurylase (MET3) were also reported as strongly upregulated in *T. melanosporum* as well as in *T. magnatum* [23]. These data are in agreement with our results, where proteomic analysis lead to the identification of 4 of these 5 differentially expressed proteins and being able to distinguish the three analyzed accessions. Furthermore, these genes resulted as also upregulated in the fruiting body when compared to free-living mycelium [23].

Despite the differences previously described, we cannot exclude that some of the differences found at the level of sulfur metabolism might be linked to the availability of sulfur in the soil itself. However, although soil analyses in productive truffle areas have already highlighted some factors that are usually associated with *T. magnatum* grounds, knowledge on the relationship on sulfur content in soil and sulfur VOCs released as well as the on requirements for *T. magnatum* life cycle, including fruiting bodies production, is still lacking. By contrast, parameters already suggested as related to truffle grounds are represented by the calcium carbonates (CaCO3), exchangeable calcium, and magnesium [12].

Calcium has been reported as an essential nutrient for the development of fruiting bodies of various *Tuber* spp., and it is provided in high quantity in the truffière to prevent limiting effects [69, 70]. It has been already suggested that in *T. magnatum*, the availability of calcium is essential for host colonization and fructification [12]. We found the calcium homeostasis protein regucalcin [71] in two differentially expressed 2-DE spots (no. 3 and 13, see Additional file 3: Table S3). Its regulation might reflect variations in calcium accessibility in the harvesting environment or suggests specific adaptations of local truffle accessions in the modulation of the Ca^2+^-dependent intracellular signaling [72].

Additional ecological interactions can be hypothesized on the basis of other differential proteins and transcripts recovered from our analysis. At the symbiotic interface with the host plant, ectomycorrhizal fungi establish a competition with root cortical cells for the monosaccharides generated from plant-derived sucrose, which are converted into storage polyols (e.g. mannitol, in *T. borchii*) [73]. Differential expression of an NADP-dependent mannitol dehydrogenase (spots 2, 4 and 5), which catalyzes fructose conversion into mannitol, is therefore suggestive of accession-specific adaptations in the symbiotic interaction. From another point of view, relevant environmental adaptations might underlie the differential regulation of peroxiredoxin (spot 8), glyoxal oxidase (spot 11) and gamma-actin (spot 3) (see Additional file 1: Table S1). A homolog of the first from *S. cerevisiae* confers resistance to H_2_O_2_ by minimizing ROS-mediated damage [74]; therefore, this observation might indicate differences in intracellular ROS management among fruiting bodies adapted to different environments. Glyoxal oxidase, instead, is a copper-containing enzyme that generates H_2_O_2_, which can be used by several ligninolytic peroxidases for lignin degradation [75–77]. Its relevance as protein biomarker was proposed in a previous work [20], where it was found as differentially expressed in *T. magnatum* fruiting bodies of different geographical origin. Finally, differential expression of gamma-actin, which is involved in fungal cell wall organization [78], might indicate a different capacity to endure environmental stresses by reinforcement of the cell wall.

## Conclusions

In conclusion, our work focused on the search for adaptive differences being able to discriminate *T. magnatum* samples from different environments, leading to new information on putative markers that could be validated in the next future on a higher number of samples. The integration of different high-throughput techniques, allowed us to identify specific molecules linked to environmental responses, with a specific attention on sulfur metabolism gene products and sulfur-containing volatile compounds, providing new information on the overall ascoma metabolism in *T. magnatum*. Although several efforts have been done in the last years to highlight the mechanisms involved in the formation of these precious fruiting bodies, the environmental factors affecting this process should be still fully elucidated. The generated datasets, which include a huge quantity of data obtained by using different -*omics* approaches, will be useful as source for researches aimed to distinguish ascocarps according to their origin, also in combination with genetic molecular marker methods. In fact, this is an important point also to avoid frauds but to also highlight new actors in the formation of this precious truffles.

## Methods

### Proteins and molecular analysis

#### Collection of truffle fruiting bodies

Fruiting bodies belonging to the various *T. magnatum* natural accessions were harvested from the natural ground in specific locations selected from Northern, Central and Southern Italian regions (respectively, San Miniato in Tuscany, Alba in Piedmont and Isernia in Molise) over different years during the same seasonal period (November), as reported in Table 1. Six samples were selected from each area at each time; samples were selected from a larger pool, according to observations at the microscope to verify the degree of maturation of the fruiting bodies. This was assessed using categorized stages, based on the percentage of asci containing mature spores, as described by Zeppa et al. [79]. The maturation stage of the spores was defined morphologically: mature spores were yellow-reddish brown, with reticulate ornamentation. Truffles were selected for the subsequent analyses when reaching stage 5 of maturation, as described by Zeppa et al. [79].

Selected fruiting bodies were thoroughly washed several times with distilled water and subsequently dipped in absolute ethyl alcohol to remove external contaminations. Finally, the thin external layer of the peridium was removed. Samples were frozen in liquid nitrogen, ground into a fine powder and, stored at − 80 °C before being used for protein and molecular analysis.

### Protein extraction

Fruiting bodies powder (100 mg) was homogenized with 1.6 mL extraction buffer (Urea 8 M, Tris-HCl 40 mM, CHAPS 4%, DTT 60 mM) according to Vita, et al. [20]. The homogenates were centrifuged at 13000 rcf at 4 °C for 15 min to eliminate debris. The extracted proteins were precipitated using 13% TCA and 0.007% ß-mercaptoethanol in acetone, transferred to − 20 °C for 2 h, and then kept at 4 °C for 2 h. Extracts were then centrifuged at 14000 rcf at 4 °C, for 15 min, and the pellet was washed twice with cold acetone (100%), re-centrifuged at the same speed, mixed with 50–500 μL extraction buffer, resuspended and centrifuged at 3000 rcf at 4 °C for 25 min.

Protein were quantified with the Bradford method [80], using bovine serum albumin (BSA) as the standard. Spectrophotometric measurements were carried out using a Cintral 101 spectrophotometer (GBC Scientific Equipment) at 595 nm in double beam mode.

### Two-dimensional electrophoresis (2-DE) analysis

Twelve or sixteen replicate gels (four for each year of collection) were performed for each biological sample, depending on the overall duration of the study for each of the sampling locations evaluated. Samples (1 mg) of protein were directly loaded by *in-gel* rehydration onto an IPG (Immobilized pH Gradient) gel strip for preparative analysis. IPG strips (18 cm, GE-Healthcare), with pH range 4–7, were rehydrated with 350 μL of IEF sample buffer (8 M urea, 2% w/v CHAPS, 40 mM DTT and 0.5% v/v IPG Buffer) containing the samples.

Strips were covered with mineral oil and focusing was carried out in an IPGphor apparatus (GE-Healthcare) applying the following conditions: 12 h of rehydration at 30 V, 1 h at 300 V (in gradient), 1 h at 300 V (step and hold), 3 h at 3500 V (in gradient), 3 h at 3500 V (step and hold), 3 h at 8000 V (in gradient) and a final step at 8000 V (step and hold until reached a total of 50,000 Vhs). After focusing, the IPG strips were equilibrated, in two steps of 15 min (first step-equilibration buffer: 50 mM Tris-HCl, pH 8.8, 8 M urea, 30% v/v glycerol, 2% w/v SDS, 40 mM DTT; second step-equilibration buffer: in the same buffer in which DTT was replaced by 40 mM iodoacetamide). The second dimension, SDS-PAGE electrophoresis, was performed using BioRad Protean II XL (20 × 20 cm) vertical gel electrophoresis chambers, on 12% (% T; total monomer concentration) acrylamide gels (Sigma Aldrich Acrylamide/Bis-acrylamide, 30% solution: ratio 29:1) applying a current of 40 mA per gel. Molecular mass standards were used, with a range from 10 to 250 kDa (Precision Plus Protein™ Unstained Protein Standards, Bio-Rad). Proteins were resolved by 2-DE and were stained with Coomassie brilliant blue (CBB, Sigma-Aldrich) according to the manufacturer’s instructions.

### Image analysis and statistical analysis

High resolution (300 dpi) images of 2-DE gels were prepared using the Densitometer GS-800 (BioRad). Computer-assisted 2D image analysis was done using the Progenesis SameSpots vs 3.2.3 gel analysis software (NonLinear Dynamics) for three technical replicates for each biological condition (different years) from three independent extraction experiment procedures (Table 1). Protein apparent relative molecular mass (Mr) was estimated by comparison with molecular weight (MW) reference markers (Precision, Bio-Rad, Hercules, CA) and pI values were assigned to the detected spots by calibration, as described in the GE-Healthcare guidelines. The protein amount was expressed as spot volume. Gel sets corresponding to samples of identical origin were subjected to pairwise comparison. Spots were considered to represent differentially expressed proteins on the basis of their ANOVA values (*p*-value) and fold change, as evaluated and automatically sorted by the software. A post-hoc analysis (Tukey’s test) was performed on the ANOVA results, to identify specific correlations among samples. The relevance of each spot in discriminating among samples of different origin was evaluated by principal component analysis (PCA), exploiting the tool available in the Progenesis SameSpot software, for differentially expressed spots.

### Protein identification by nano-liquid chromatography-electrospray-linear ion trap-tandem mass spectrometry (nanoLC-ESI-LIT-MS/MS)

Nineteen spots were manually excised from gels, triturated and washed with water. Proteins were *in-gel* reduced, S-alkylated with iodoacetamide and digested with trypsin, overnight. Digest aliquots were removed and subjected to a desalting/concentration step on C18 ZipTip microcolumn using 5% formic acid/50% acetonitrile as eluent before further analysis. Digests were then analyzed by nanoLC-ESI-LIT-MS/MS using a LTQ XL mass spectrometer (Thermo Finnigan, San Jose, CA, USA) equipped with Proxeon nanospray source connected to an Easy-nanoLC (Proxeon, Odense, Denmark). Peptide mixtures were separated on an Easy C18 column (100 × 0.075 mm, 3 μm) using a linear gradient from 5 to 50% of acetonitrile in 0.1% formic acid, over 24 min, at a flow rate of 300 nL/min. Spectra were acquired in the range *m/z* 400–2000. The acquisition was controlled by a data-dependent product ion scanning procedure over the three most abundant ions, enabling dynamic exclusion (repeat count 1 and exclusion duration 1 min). The mass isolation window and collision energy were set to *m/z* 3 and 35%, respectively.

MASCOT software package (Matrix Science, UK) was used to identify protein spots unambiguously from an updated tuber non-redundant sequence database from NCBI by using a mass tolerance value of 2.2 Da for precursor ion and 0.8 Da for fragment ions, trypsin as proteolytic enzyme, a missed cleavages maximum value of 2 and Cys carbamidomethylation and Met oxidation as fixed and variable modification, respectively. Candidates with more than 2 assigned peptides with MASCOT score > 25 (*p* < 0.01 for a significant identification) were further evaluated by the comparison of their calculated mass value with that obtained from 2-DE. Where appropriate, protein identification was checked manually to provide for a false positive rate of less than 1%. Identified proteins were then sorted basing on their exponentially modified protein abundance index (emPAI) for each candidate spots [81]; this index allows to estimate relative quantification based on protein coverage.

Proteins obtained without functional identification were then used for Protein Blast Analysis (UniProtKB blast p, whole database) performed with default settings.

### GO enrichment

Gene ontology (GO) term enrichment analysis to find statistically over- or down-represented categories was performed with BiNGO 3.03 [82] as a plugin for Cytoscape 3.6.0 [83]; the latest available ontology (obo 1.2 format) and *Tuber* spp. annotations files were downloaded respectively from the Gene Ontology and the Gene Ontology Annotation (GOA) websites (https://www.ebi.ac.uk/GOA/proteomes). Hypergeometric test, Benjamini & Hochberg false discovery rate [84] correction and a significance level of 0.05 were chosen as parameters to visualize in Cytoscape the over-represented categories after correction.

### Total RNA extraction

RNA extraction was performed using the selected samples (AL, IS, SM) collected during two different years: 2014 and 2015. With the aim of reducing the variability among biological replicates, for each location and year of collection, six different fruiting bodies were homogenized and mixed together to obtain a single data point. Samples collected in the same location at different time were considered biological replicates, resulting in experimental design comprising 3 locations and 2 time points, for a total of six samples. Total RNA extraction was achieved using the Plant/Fungi Total RNA Purification Kit (Norgen Biotek Corp) according to the manufacturer’s method. Total RNA integrity and purity was further checked on agarose gel and Agilent 2100 Bioanalyzer High Sensitivity and DNA 1000 assay (Agilent Technologies, Santa Clara, CA).

### Illumina sequencing

Illumina stranded poly(A)^+^ RNA-seq libraries were generated from the six samples according to the TruSeq mRNA Sample Prep kit (Illumina, San Diego, CA) and subjected to single-end 100-bp reads (1X100 bp) sequencing at IGATech (Udine, Italy) using a HiSeq2000 platform (Illumina, San Diego, CA). The CASAVA v1.8.2 of the Illumina pipeline was used to process raw data for format conversion and de-multiplexing. On average, ~ 41 million reads per sample were produced with a total of ~ 245 million reads.

### RNA-seq analysis

Raw reads were quality evaluated before the data analysis using the program FastQC v0.11.5 [85]. A quality score above Q30 was kept to maintain high accuracy in the downstream analysis. Undefined bases (Ns) within the reads and the presence of sequencing adapters were excluded with the program Cutadapt (version 1.8.3) [86]. Read mapping and transcript abundance were estimated using salmon (v0.9.1) [87] in quasi-mapping mode with the option --numBootstraps 30. The transcriptome index was built from the 12,367 de novo reconstructed *T. magnatum* high-confidence protein-coding transcripts published by Vita et al. [33] with the options –keepDuplicates and --type quasi. Parameters not specified were run as default. Salmon outputs were imported into R with the Bioconductor package tximport (v1.0.3) [88] and the transcript TPM abundance was summarized to gene level abundance using the gene models (*n* = 6723) published by *Vita* et al. [33]. The differential expression (DE) analysis was conducted using the Bioconductor package DESeq2 [89] setting the variable location as condition in the design formula. Briefly, raw counts were imported using the functions DESeqDataSetFromTximport. Data were filtered for row sum counts > 1, resulting in *n* = 6665 genes. DE pairwise comparisons between the three locations, using Alba as control, were carried out according to the Wald Test with a FDR = 5%. Sample specificity was assessed calculating the Shannon entropy for each gene expression profile using the Bioconductor package BioQC with the function entropySpecificity [90].

RNA-seq data from this study have been submitted to the NCBI Sequence Read Archive (SRA; http://www.ncbi.nlm. nih.gov/sra/) under BioProject PRJNA501857.

### Real-time PCR analysis

Total RNA was extracted as reported above for RNA-seq analysis. Electrophoresis using 1% agarose gel was performed for all RNA samples to check for RNA integrity, followed by spectrophotometric quantification and quality control. RNA samples were then subjected to DNase treatment using a Turbo DNA-free kit (Ambion, USA) to remove possible DNA contamination. RNA was then reverse-transcribed using SuperScript® III Reverse Transcriptase kit (Life Technologies, UK) with random primers. Gene expression analysis was carried out using an ABI Prism 7300 sequence detection system (Applied Biosystems, USA) as described by Bui et al. [91]. Quantitative PCR was performed using 15 ng cDNA and iQ™ Sybr Green Supermix (BioRad Laboratories), according to the manufacturer’s instructions. Three technical replicates were performed for each biological replicate (n = 6).

Primers were designed using the sequence information derived from the de novo transcriptome assembly published by Vita et al. [33]. Comparison results between reference *T. melanosporum* and *T. magnatum* sequences were reported in Supporting Information (see Additional file 13: Table S9). Expression of *T. magnatum* (AF054901) 18S rRNA was used as the housekeeping gene. Relative gene expression levels were calculated with the 2^-ΔΔCt^ method [92]. Primers are listed in Supporting Information (see Additional file 13: Table S9). We first reconstructed the metabolic pathway of sulfur compounds in *T. magnatum*, starting from the one described in *T. melanosporum* by [22]. The choice of using *T. melanosporum* genetic information was due by the lacking of genetic information about the genome of *T. magnatum* when this work was originally designed. The publication of the work by Murat et al. [23] made available new information about *T. magnatum* including the sulfur pathway genes. On this basis, we browsed the sequences from the previous transcriptome profiling by sequence similarity, using genes belonging to the annotated *T. melanosporum* genome [22] as queries, in order to define orthologous genes in *T. magnatum* (see Additional file 13: Table S9).

### Mass spectrometry analysis of VOC compounds

#### GC-MS analysis

##### SPME method

Briefly, 1 g of fresh sample was placed in a 20 mL crimped vial. Solid-phase microextraction (SPME) was carried out in the headspace mode using an autosampler AOC-5000 (Shimadzu) equipped with a fused silica fiber coated with a 50/30 μm layer of divinylbenzene/carboxen/polydimethylsiloxane, 1 cm long (MilliporeSigma, Bellefonte, Pennsylvania, USA). The fiber was conditioned according to manufacturer’s instructions. Samples were conditioned for 5 min at 50 °C, under agitation (clockwise, rotation at 500 rpm), before exposing the fiber for 20 min at 50 °C, under continuous agitation. Analytes were then desorbed for 1 min at 260 °C in the GC injector in splitless mode (1 min). Each sample was analyzed in triplicate. Raw data related to the first 2 years of analysis (2014–2015) are already reported and processed with different statistical methods [52].

#### GC–MS and GC-FID analysis

GC–MS and GC-FID runs were carried out on a two parallel GC-QP2010 and GC2010 instruments (Shimadzu, Kyoto, Japan). The GC column used was a 30 m × 0.25 mm i.d. × 0.25 μm df Supelcowax-10 column (Millipore-Sigma). Helium was exploited as carrier gas, at a constant linear velocity of 30.0 cm/s, which corresponded to an inlet pressure of 26.4 kPa for GC-MS and 97.4 kPa for GC-FID. The temperature program was the same in both analysis-type: 40 °C at 3 °C/min to 250 °C, at 10 °C/min to 280 °C, held 10 min.

GC-MS ion source temperature was set at 200 °C; the interface temperature at 250 °C. Scan range was set to m/z 40–360, with a scanning rate of 2000 amu/s. FFSNC 3.0 (Shimadzu) and NIST11 (Wiley) commercial libraries were used for identification, applying two filters, namely a spectrum similarity match over 85% and Linear Retention Index (LRI) (related to a C4-C24 FAMEs mixture) agreement in the ±15 range.

The FID temperature was set at 280 °C (sampling rate 40 ms) and gas flows were 40 mL/min for hydrogen and 400 mL/min for air, respectively.

The data handling was supported by GCMSsolution ver.4.30 and GCsolution software (Shimadzu) for GC-MS and GC-FID analysis, respectively.

#### Quantitative PTR-ToF-MS 8000 analysis

Samples were subjected to analysis of VOCs. Accurate analysis of VOCs took advantage by the use of an innovative instrument, such as PTR-MS [53, 93–95], and its upgraded version PTR-ToF-MS 8000, having increased resolution coupling with the time of flight (ToF) mass analyzer. In particular, the use of a PTR-ToF instrument expanded the mass range (*m/z*) of identifiable compounds to small molecules (e.g. < 70 amu), for which other spectrometers do not show the required sensitivity. VOCs emitted from samples were collected from each area (AL, IS, SM) during 4 different years (2014–2017); details are reported in Table 1. Raw data related to the first 2 years of analysis (2014–2015) are already reported and processed with different statistical methods [52]. The analysis was mainly focused in the range from 30 to 120 *m/z* as previously reportedin Vita et al. [52]. Volatiles were analyzed with a PTR-ToF-MS 8000 (IoniconAnalytik GmbH, Innsbruck, Austria) using H_3_O^+^ as reagent ion for the proton transfer reaction. The reaction takes place between H_3_O^+^ ions and all the biogenic or anthropogenic VOCs having a proton affinity higher than that of water (165.2 kcal mol^− 1^). Separation of single ions happens accordingly to their mass to charge (*m/z*) ratio. Drift applied voltage was set at 600 V, temperature at 110 °C, and pressure at 2.25 mbar. For each sample, about 6 g of material was transferred in a glass jar provided with a special lid that allowed Teflon connection to a zero-air generator (inlet) and the PTR-ToF system (outlet). The head space was then measured by direct injection into the PTR-ToF drift tube inlet for 150 s, after respectively 1 and 20 min of exposure.

Preliminary measurements on an empty jar were run before every experiment and used for background subtraction. All mass spectra up to *m/z* = 315 were simultaneously detected and recorded with 1 s as integration time. Internal calibration was based on *m*/*z* = 18.0338 (NH_4_^+^), *m/z* = 21.0202 (H_3_^18^O^+^) and *m/z* = 29.9974 (NO^+^). For more detailed explanation see Lindinger et al. [96] and Brilli et al. [97]. Spectra raw data (count rate of the analytes recorded expressed in number of counts per second, *cps*) were acquired with TofDaq software (Tofwerk AG, Switzerland). For each sample, the average data resulting from consecutive 20 s of measurement were extracted after 1 and 20 min from the beginning of the experiment. At least six technical replicates were performed for each sample as well as each year of analysis.

Quantitative differences (ppb values) data were then primarily normalized on the base of the surface/weight rapport of the analyzed fruiting bodies (sup/vol).

#### Statistical analysis of VOCs results

Multivariate partial least squares-discriminant analyses (PLS-DA) (supervised method) were applied on the 165 VOCs spectra from 64 *T. magnatum* samples obtained by GC-MS (4 years) and 65 VOCs spectra from 92 samples obtained by PTR-ToF-MS (4 years), respectively. Two distinct models were built up to compare the ability of the different chemometric approach to correctly classify the three different genotypes of truffles, independently from the sampling time. As a pre-processing step, data were submitted to a logarithmic (log_10_ + 1) transformation and auto-scaling. The whole data set was each time split into training and validation subsets, optimally chosen with the Euclidean distances based on the algorithm of Kennard and Stone [98]. The training data set (about 80% of the samples) was used for selecting the optimal number of latent variables (LVs) throughout the calibration and cross-validation phases; the test set (about 20% of samples previously removed from the data set) was used to predict the class membership (external validation). The training set was used to fit a model based on Venetian blinds cross-validation procedures, evaluated by the number of correct predictions and the root-mean-squared error of cross-validation (RMSECV), and validated with the removed samples (external validation set). External validation of the model was quantified by the root-mean-squared error of prediction (RMSEP). The optimal number of LVs was selected as those associated with the minimum error and misclassification rate of the calibration dataset. The reliability of the model was tested by confusion matrices. The threshold to assign a sample to a class was chosen to minimize the number of false positives and false negatives (Bayes theorem). Variable Importance in Projection (VIP) scores (*p* = 0.01) were also calculated. A random permutation of the class labels (permutation test) was also performed (500 iterations), with the aim to generate nonsense datasets for comparison with the true model and to evaluate the probability that the model was significantly different from one casually built up under the same conditions. PLS-DA was performed by PLS-Toolbox v. 8.0.2 (Eigenvector Research Inc., West Eaglerock Drive, Wenatchee, WA) for MATLAB® R2015b (Mathworks Inc., Natick, MA, USA). Data from PTR-ToF analysis were then submitted to Canonical correspondence analysis (CCoA), with the aim to detect possible correlations among VOC and climatic conditions. This method allows comparing two sets of variables, extracting ordination axes that are linear combinations of VOCs (criterion variables) explaining at the same time as much as possible of the variance in the environmental data (explanatory variables, see Additional file 21: Table S16). The samples were ordered with the components maximally interpreting the environmental data as well. CCoA analysis was performed using SYN-TAX 2000, Ordination package [99].

## Supplementary information

**Additional file 1: Table S1.** Summary results of post-hoc tests performed on quantitative protein spot data obtained from 2-DE gel analysis of Alba (AL), San Miniato (SM) and Isernia (IS) fruiting bodies.

**Additional file 2: Table S2.** Protein identified by nanoLC-ESI-LIT-MS/MS analysis. Proteins were sorted based on their exponentially modified Protein Abundance Index (emPAI). Shadings highlight uncharacterized proteins.

**Additional file 3: Table S3.** Additional information related to the uncharacterized and predicted proteins identified through mass spectrometry.

**Additional file 4: Table S4.** Summary of sulfur-related proteins identified by nanoLC-ESI-LIT-MS/MS analysis.

**Additional file 5: Table S5.** RNA-seq statistics from Vita et al. [33].

**Additional file 6: Figure S1.** Euclidean distance among samples. The heat map shows sample to samples distances indicating the strong correlation between biological replicates. The distance matrix was calculated from the normalized expression dataset using the variance-stabilizing transformations function from the Bioconductor package DESeq2. Data were hierarchically clustered based on sample distances. Biological replicates are indicated as r1 (year 2014) and r2 (year 2015). Shades of grey represent different extents of correlation among samples; black represents perfect positive correlation.

**Additional file 7: Figure S2.** Volcano plots representing the differentially expressed genes based on RNA-seq data. Pairwise comparisons are shown for San Miniato vs Alba (**a**) and Isernia vs Alba (**b**). Yellow dots highlight DEGs selected for |log2 fold change| > 1.5 and FDR < 0.05.

**Additional file 8: Table S6.** Top 100 transcripts related to San Miniato – Alba comparison (differential expression analysis results).

**Additional file 9: Table S7.** Top 100 transcripts related to Isernia – Alba comparison (differential expression analysis results).

**Additional file 10: Figure S3.** Sample gene specificity. Shannon entropy (SH) distribution of *T. magnatum* genes (*n* = 6665) based on the expression data (in transcripts per million, TPM). A SH coefficient > 0.6 represents the gene-specific expression associated to each *T. magnatum* ecotype (i.e. geographical accession, see Additional file 11: Data file S1).

**Additional file 11: Data file S1.** Differentially expressed genes (DEGs) data related to sample comparisons. Data sheets A) DEGs related to comparison San Miniato (SM) vs Alba (AL); B) DEGs related to comparison Isernia (IS) vs Alba (AL); C) DEGs identified in both the comparisons (SMvsAL, ISvsAL); D) DEGs identified only in San Miniato (SM) vs Alba (AL); E) DEGs identified only in Isernia (IS) vs Alba (AL); F) Gene specificty reported in TPM (transcripts per million) for each sample.

**Additional file 12: Table S8.** Sample-specific expression of *T. magnatum* genes involved in sulfur metabolism.

**Additional file 13: Table S9.** *T. magnatum* genes involved in the sulfur metabolism and annotated in *T. melanosporum*.

**Additional file 14: Figure S4.** Relative expression level of the genes selected for the qPCR. Relative levels were expressed, for each gene, as fold change (FC) from the reference sample SM 2014. Data are mean values of transformed data (log_10_(FC + 1) related to gene expression (*n* = 4), calculated with the 2^-ΔΔCt^ method [92]. Letters indicate results of Tukey post-hoc test analysis. Sample names correspond to those reported in Table 1. For a reference to RNA-seq transcript IDs, see Additional file 13: Table S9.

**Additional file 15: Table S10.** Compounds identified through GC-MS analysis.

**Additional file 16: Table S11.** PLS-DA (GC MS model) statistics for each Y-Block (class 1 = Alba; class 2 = Isernia; class 3 = San Miniato) related to 64 truffle samples.

**Additional file 17: Table S12.** VIPs compounds (GC-MS data) according to PLS-DA analysis. Compounds that displayed VIP scores ≥2 (bold marked) in at least in one sample are shown.

**Additional file 18: Table S13.** Compounds identified through PTR-ToF analysis conducted during 4 years of experimental work.

**Additional file 19: Table S14.** PLS-DA (PTR-ToF-MS model) statistics for each Y-Block (class 1 = Alba; class 2 = Isernia; class 3 = San Miniato) related to 92 truffle samples.

**Additional file 20: Table S15.** VIPs compounds (PTR-ToF data) according to PLS-DA analysis.

**Additional file 21: Table S16.** Climatic parameters on the fruiting bodies sampling area for the four-year period 2014–2017 used for statistical analysis of PTR-ToF data (CCoA).

## Data Availability

Original raw RNA-seq data generated by Vita et al. [33] can be found at NCBI Bioproject PRJNA501857 and are also accessible through the link https://figshare.com/s/01ec86ee5e0f657ab955. 2-DE gel images are available in figshare: https://figshare.com/s/97e9c0089aaf4a9b0def. Metabolomics data related to GC-MS (https://figshare.com/s/07ec3000f9e6132afb41) and PTR-ToF-MS (https://figshare.com/s/e1c5acb89141e809a8a6) raw spectra were deposited in figshare. Proteomics MS raw data has been deposited in figshare (https://figshare.com/s/7a81e9ea46ad71d5598b).

## Abbreviations

2-DE: Two-dimensional electrophoresis
AHC: Aggregative hierarchical clustering
AHCY: Adenosylhomocysteinase
ANOVA: Analysis of variance
CCOA: Canonical correspondence analysis
CTH: Cystathionine gamma-lyase
DEGs: Differentially expressed genes
DMS: Dimethyl sulfide
DMSO: Dimethyl trisulfide
emPAI: Exponentially modified protein abundance index
FDR: False discovery rate
GC-MS: Gas-chromatography-mass spectrometry
GO: Gene ontology
GOA: Gene ontology annotation
IPG: Immobilized pH Gradient
MetE: Cobalamin-independent Met synthase
Msra: Peptide methionine sulfoxide reductase
PAGE: Polyacrylamide gel electrophoresis
PCA: Principal component analysis
PLS-DA: Partial least squares-discriminant analysis
PTR-ToF: Proton transfer reaction-mass spectrometry
SAM: S-adenosylmethionine synthase
SPME: Solid-phase microextraction
TPM: Transcripts per million
VIP: Variable Importance in the Projection
VOCs: Volatile organic compounds
