# Supplementary material for: Tuberomics: a molecular profiling for the adaption of edible fungi (Tuber magnatum Pico) to different natural environments
Source: BMC Genomics. 2020 Jan 29;21:90. doi: 10.1186/s12864-020-6522-3 (PMC6988325; doi:10.1186/s12864-020-6522-3)
Supplement: Supplementary file 1 — Additional file 1: Table S1. Summary results of post-hoc tests performed on quantitative protein spot data obtained from 2-DE gel analysis of Alba (AL), San Miniato (SM) and Isernia (IS) fruiting bodies. [file 12864_2020_6522_MOESM1_ESM.docx]

**Table S1:** **Summary results of post-hoc tests performed on quantitative protein spot data obtained from 2-DE gel analysis of Alba (AL), San Miniato (SM) and Isernia (IS) fruiting bodies.** A Tukey HSD test was applied to the pairwise comparison of the analyzed samples. Data are reported as p-values (*, 0.01 < P ≤ 0.05; **, 0.001 < P ≤ 0.01; ***, 0.0001 <P ≤ 0.001; ****, P ≤ 0.0001).

| **Spot rank** | **AL vs IS** | **AL vs SM** | **IS vs SM** |
| --- | --- | --- | --- |
| **1** | ** | **** | ** |
| **2** | ** | **** | ** |
| **3** | * | **** | * |
| **4** | * | **** | * |
| **5** |  | **** | * |
| **6** | **** |  | *** |
| **7** |  | **** |  |
| **8** |  | **** | ** |
| **9** | ** | * | **** |
| **10** | * |  | ** |
| **11** |  | * | ** |
| **12** | * | * |  |
| **13** | * |  |  |
| **14** | * |  | * |
| **15** |  |  | * |
| **16** |  | * |  |
| **17** |  |  |  |
| **18** |  | * |  |
| **19** |  |  |  |
