# Supplementary material for: Tuberomics: a molecular profiling for the adaption of edible fungi (Tuber magnatum Pico) to different natural environments
Source: BMC Genomics. 2020 Jan 29;21:90. doi: 10.1186/s12864-020-6522-3 (PMC6988325; doi:10.1186/s12864-020-6522-3)
Supplement: Supplementary file 2 — Additional file 2: Table S2. Protein identified by nanoLC-ESI-LIT-MS/MS analysis. Proteins were sorted based on their exponentially modified Protein Abundance Index (emPAI). Shadings highlight uncharacterized proteins. [file 12864_2020_6522_MOESM2_ESM.docx]

**Table S2: Protein identified by ESI-Quad TOF analysis.** Proteins were sorted based on their exponentially modified Protein Abundance Index (emPAI). Shadings highlight uncharacterized proteins.

| **Spot no. (a)** | **Protein list** | **Acc. No. (b)** | **Organism** | **Protein name** | **Score (c)** | **Seq. Cov. % (d)** | **Mw Kda Obs/ Theo (e)** | | **pI Obs/ Theo (f)** | | **Mat./ Unique Peptides (g)** | | **emPAI (h)** |
| --- | --- | --- | --- | --- | --- | --- | --- | --- | --- | --- | --- | --- | --- |
| **1** | **1** | **D5GNQ7** | *Tuber melanosporum* | **Uncharacterized protein** | 326 | 23.6 | 60.4 | 40.9 | 5.71 | 5.64 | 8 | 6 | 0.69 |
|  | **2** | **D5G9M7** | *Tuber melanosporum* | **Uncharacterized protein** | 246 | 11.2 | 60.4 | 57.8 | 5.71 | 9.93 | 4 | 4 | 0.28 |
|  | **3** | **D5GJ78** | *Tuber melanosporum* | **S-adenosylmethionine synthase** | 104 | 5.2 | 60.4 | 41.8 | 5.71 | 5.70 | 2 | 2 | 0.19 |
| **2** | **4** | **D5G736** | *Tuber melanosporum* | **Uncharacterized protein** | 796 | 50.3 | 60.4 | 38.3 | 5.62 | 5.62 | 19 | 13 | 3.47 |
|  | **5** | **Q86ZU6** | *Tuber borchii* | **Glutamine synthetase (GS) (EC 6.3.1.2)** | 109 | 9.2 | 60.4 | 40.2 | 5.62 | 5.79 | 2 | 2 | 0.2 |
| **3** | **6** | **D5GNQ7** | *Tuber melanosporum* | **Uncharacterized protein** | 417 | 24.7 | 62.5 | 40.9 | 5.52 | 5.64 | 10 | 8 | 1.2 |
|  | **7** | **D5GI98** | *Tuber melanosporum* | **Uncharacterized protein** | 255 | 15.5 | 62.5 | 41.0 | 5.52 | 5.54 | 5 | 5 | 0.55 |
|  | **8** | **D5GJ78** | *Tuber melanosporum* | **S-adenosylmethionine synthase** | 205 | 14.9 | 62.5 | 41.8 | 5.52 | 5.70 | 4 | 4 | 0.41 |
|  | **9** | **D5G471** | *Tuber melanosporum* | **Uncharacterized protein** | 92 | 10.1 | 62.5 | 22.1 | 5.52 | 8.41 | 2 | 2 | 0.38 |
|  | **10** | **D5GDD6** | *Tuber melanosporum* | **Uncharacterized protein** | 199 | 19.3 | 62.5 | 38.0 | 5.52 | 6.73 | 3 | 3 | 0.33 |
|  | **11** | **D5GM51** | *Tuber melanosporum* | **Uncharacterized protein** | 243 | 13.1 | 62.5 | 44.2 | 5.52 | 5.87 | 4 | 4 | 0.28 |
|  | **12** | **D5G5R4** | *Tuber melanosporum* | **Uncharacterized protein** | 156 | 7.1 | 62.5 | 46.9 | 5.52 | 5.97 | 3 | 2 | 0.26 |
|  | **13** | **D5G7I0** | *Tuber melanosporum* | **ATP synthase subunit beta** | 147 | 7.9 | 62.5 | 51.8 | 5.52 | 5.08 | 3 | 3 | 0.23 |
| **4** | **14** | **D5G918** | *Tuber melanosporum* | **Eukaryotic translation initiation factor 3 subunit F (eIF3f)** | 136 | 11.4 | 60.4 | 35.8 | 5.82 | 5.27 | 3 | 3 | 0.35 |
|  | **15** | **D5GDG9** | *Tuber melanosporum* | **Glutamine synthetase (EC 6.3.1.2)** | 139 | 9.3 | 60.4 | 49.7 | 5.82 | 7.95 | 5 | 3 | 0.34 |
|  | **16** | **Q1ACW3** | *Tuber borchii* | **NADP-dependent mannitol dehydrogenase** | 116 | 8.1 | 60.4 | 38.0 | 5.82 | 5.69 | 2 | 2 | 0.21 |
| **5** | **17** | **D5G736** | *Tuber melanosporum* | **Uncharacterized protein** | 558 | 38.2 | 60.4 | 38.3 | 5.56 | 5.62 | 12 | 8 | 1.55 |
|  | **18** | **D5GA95** | *Tuber melanosporum* | **Uncharacterized protein** | 110 | 9.5 | 60.4 | 28.6 | 5.56 | 5.78 | 4 | 2 | 0.64 |
|  | **19** | **D5G5R4** | *Tuber melanosporum* | **Uncharacterized protein** | 232 | 11.3 | 60.4 | 46.9 | 5.56 | 5.97 | 3 | 3 | 0.26 |
| **6** | **20** | **D5GLV9** | *Tuber melanosporum* | **Uncharacterized protein** | 438 | 44.1 | 43.2 | 32.9 | 5.43 | 5.27 | 19 | 10 | 3.58 |
|  | **21** | **D5GAF9** | *Tuber melanosporum* | **Uncharacterized protein** | 572 | 35.2 | 43.2 | 33.0 | 5.43 | 6.02 | 14 | 10 | 2.67 |
|  | **22** | **D5GA85** | *Tuber melanosporum* | **Malate dehydrogenase** | 137 | 7.5 | 43.2 | 37.8 | 5.43 | 6.45 | 3 | 2 | 0.33 |
| **7** | **23** | **D5GM11** | *Tuber melanosporum* | **Uncharacterized protein** | 1070 | 30.6 | 99.3 | 71.8 | 4.73 | 4.88 | 24 | 20 | 2.19 |
|  | **24** | **D5G7L2** | *Tuber melanosporum* | **Uncharacterized protein** | 268 | 18.4 | 99.3 | 49.7 | 4.73 | 4.85 | 7 | 5 | 0.66 |
|  | **25** | **D5G7D0** | *Tuber melanosporum* | **1,3-beta-glucanosyltransferase (EC 2.4.1.-)** | 177 | 8.5 | 99.3 | 48.4 | 4.73 | 4.83 | 4 | 4 | 0.35 |
| **8** | **26** | **D5GI33** | *Tuber melanosporum* | **Uncharacterized protein** | 78 | 9.2 | 24.6 | 21.7 | 5.2 | 5.08 | 2 | 2 | 0.38 |
|  | **27** | **D5GET6** | *Tuber melanosporum* | **Uncharacterized protein** | 106 | 9.5 | 24.6 | 22.4 | 5.2 | 10.6 | 2 | 2 | 0.37 |
|  | **28** | **D5GNP5** | *Tuber melanosporum* | **Adenosylhomocysteinase** | 142 | 9.4 | 24.6 | 46.1 | 5.2 | 6.02 | 3 | 3 | 0.26 |
| **9** | **29** | **D5GAV4** | *Tuber melanosporum* | **Uncharacterized protein** | 142 | 3.9 | 22.4 | 85.6 | 5.36 | 6.35 | 3 | 3 | 0.14 |
| **10** | **30** | **D5G7S8** | *Tuber melanosporum* | **Uncharacterized protein** | 156 | 22.9 | 21.2 | 17.5 | 5.48 | 9.99 | 2 | 2 | 0.49 |
|  | **31** | **D5GM67** | *Tuber melanosporum* | **Protein phosphatase PP2A regulatory subunit B** | 92 | 5.6 | 21.2 | 49.2 | 5.48 | 5.38 | 2 | 2 | 0.16 |
|  | **32** | **D5GF65** | *Tuber melanosporum* | **NAD-specific glutamate dehydrogenase (EC 1.4.1.2)** | 111 | 1.4 | 21.2 | 127 | 5.48 | 6.36 | 3 | 3 | 0.06 |
| **11** | **33** | **D5GJY5** | *Tuber melanosporum* | **Uncharacterized protein** | 331 | 12.6 | 83.5 | 86.3 | 5.2 | 5.16 | 11 | 6 | 0.59 |
|  | **34** | **D5GK33** | *Tuber melanosporum* | **Uncharacterized protein** | 206 | 9.5 | 83.5 | 62.3 | 5.2 | 5.49 | 4 | 4 | 0.26 |
|  | **35** | **D5G7I0** | *Tuber melanosporum* | **ATP synthase subunit beta** | 95 | 4.4 | 83.5 | 51.8 | 5.2 | 5.08 | 2 | 2 | 0.15 |
| **12** | **36** | **D5G966** | *Tuber melanosporum* | **Superoxide dismutase [Cu-Zn]** | 293 | 23.6 | 19.9 | 25.5 | 5.4 | 6.29 | 11 | 4 | 1 |
| **13** | **37** | **D5GJ78** | *Tuber melanosporum* | **S-adenosylmethionine synthase (EC 2.5.1.6)** | 872 | 35.9 | 60.4 | 41.8 | 5.93 | 5.70 | 15 | 12 | 2.63 |
|  | **38** | **D5GNQ7** | *Tuber melanosporum* | **Uncharacterized protein** | 357 | 20.7 | 60.4 | 40.9 | 5.93 | 5.64 | 8 | 6 | 0.85 |
|  | **39** | **D5G9M7** | *Tuber melanosporum* | **Uncharacterized protein** | 108 | 6.3 | 60.4 | 57.8 | 5.93 | 9.93 | 3 | 2 | 0.21 |
| **14** | **40** | **D5G3Z0** | *Tuber melanosporum* | **Nucleoside diphosphate kinase (EC 2.7.4.6)** | 133 | 29.2 | 14.6 | 15.1 | 6.2 | 7.00 | 3 | 3 | 0.99 |
|  | **41** | **D5GPG5** | *Tuber melanosporum* | **Ketol-acid reductoisomerase, mitochondrial (EC 1.1.1.86)** | 201 | 10.2 | 14.6 | 45.0 | 6.2 | 9.04 | 4 | 4 | 0.27 |
| **15** | **42** | **D5G4J8** | *Tuber melanosporum* | **Uncharacterized protein** | 207 | 3.0 | 87.7 | 66.2 | 6.05 | 5.48 | 3 | 2 | 0.18 |
| **16** | **43** | **D5G8F0** | *Tuber melanosporum* | **Uncharacterized protein** | 159 | 24.0 | 22.4 | 20.0 | 5.84 | 5.41 | 4 | 3 | 0.7 |
| **17** | **44** | **D5GK33** | *Tuber melanosporum* | **Uncharacterized protein** | 1587 | 40.4 | 90.3 | 62.3 | 5.30 | 5.49 | 57 | 28 | 11.78 |
|  | **45** | **D5G7Z9** | *Tuber melanosporum* | **Sulfate adenylyltransferase** | 293 | 12.0 | 90.3 | 63.3 | 5.30 | 6.87 | 6 | 6 | 0.33 |
|  | **46** | **D5GGZ0** | *Tuber melanosporum* | **Uncharacterized protein** | 166 | 9.9 | 90.3 | 60.7 | 5.30 | 5.46 | 3 | 3 | 0.2 |
|  | **47** | **D5G9B7** | *Tuber melanosporum* | **Uncharacterized protein** | 159 | 8.8 | 90.3 | 65.7 | 5.30 | 5.43 | 3 | 3 | 0.18 |
| **18** | **48** | **D5G830** | *Tuber melanosporum* | **Uncharacterized protein** | 96 | 10.6 | 15.6 | 17.9 | 5.2 | 4.97 | 2 | 2 | 0.48 |
| **19** | **49** | **D5G7I0** | *Tuber melanosporum* | **ATP synthase subunit beta** | 1804 | 73.8 | 75.0 | 51.8 | 5.26 | 5.08 | 69 | 28 | 31.3 |
|  | **50** | **D5G4P4** | *Tuber melanosporum* | **Uncharacterized protein** | 332 | 26.1 | 75.0 | 40.1 | 5.26 | 4.95 | 8 | 7 | 1.05 |
|  | **51** | **D5GFC4** | *Tuber melanosporum* | **Uncharacterized protein** | 266 | 15.1 | 75.0 | 48.4 | 5.26 | 5.22 | 5 | 5 | 0.45 |
|  | **52** | **D5G5F0** | *Tuber melanosporum* | **Carboxypeptidase** | 94 | 3.9 | 75.0 | 60.1 | 5.26 | 6.23 | 2 | 2 | 0.13 |

(**a**) Spot numbers correspond to those reported in Fig. 1; (**b**) UniProtKB accession number; (**c**) Mascot score; (**d**) percent sequence coverage; (**e**) gel-observed vs. theoretical molecular weights; (**f**) observed vs. theoretical isoelectric points; * Observed and theoretical weight/pi may differ on the base of the real molecular weight of the protein in *T. melanosporum*; (**g**) number of matched and unique peptides; (**h**) emPAI score, referred to relative quantitation of protein.
