# Supplementary material for: Tuberomics: a molecular profiling for the adaption of edible fungi (Tuber magnatum Pico) to different natural environments
Source: BMC Genomics. 2020 Jan 29;21:90. doi: 10.1186/s12864-020-6522-3 (PMC6988325; doi:10.1186/s12864-020-6522-3)
Supplement: Supplementary file 3 — Additional file 3: Table S3. Additional information related to the uncharacterized and predicted proteins identified through mass spectrometry. [file 12864_2020_6522_MOESM3_ESM.docx]

**Table S3: Additional information related to the uncharacterized and predicted proteins isolated through mass spectrometry.** For the non-annotated peptides reported in Additional file 2: Table S2, the most closely related accessions present in the RefSeq protein database were retrieved by BLAST analysis.

| **Spot No. (a)** | **Relationship list (b)** | **Acc. No. (c)** | **Protein Name** | **Acc. No. (d)** | **Organism** | **Protein Length (e)** | **E-Value (f)** |
| --- | --- | --- | --- | --- | --- | --- | --- |
| **1** | **1** | **D5GNQ7** | **Putative calcium homeostasis protein Regucalcin** | **OOQ84823.1** | *Penicillium brasilianum* | 308 | 1e-54 |
| **1** | **2** | **D5G9M7** | **RNP domain-containing protein** | **EEH09099.1** | Histoplasma capsulatum G186AR | 471 | 5e-96 |
| **2** | **4** | **D5G736** | **NADP-dependent mannitol dehydrogenase** | **ABB55877.1** | *Tuber borchii* | 345 | 0.0 |
| **3** | **6** | **D5GNQ7** | **Putative calcium homeostasis protein Regucalcin** | **OOQ84823.1** | *Penicillium brasilianum* | 308 | 1e-54 |
| **3** | **7** | **D5GI98** | **KH domain RNA-binding protein** | **XP_002850758.1** | *Arthroderma otae CBS 113480* | 367 | 6e-161 |
| **3** | **9** | **D5G471** | **Woronin body major protein** | **OTA32567.1** | *Hortaea werneckii EXF-2000* | 222 | 2e-76 |
| **3** | **10** | **D5GDD6** | **Gamma actin** | **XP_008717207.1** | *Cyphellophora europaea CBS 101466* | 332 | 4e-177 |
| **3** | **11** | **D5GM51** | **Cystathionine gamma-lyase** | **KKK16227.1** | *Aspergillus rambellii* | 411 | 0.0 |
| **3** | **12** | **D5G5R4** | **Fructose-bisphosphate aldolase 1** | **EWC47337.1** | *Drechslerella stenobrocha 248* | 360 | 0.0 |
| **5** | **17** | **D5G736** | **NADP-dependent mannitol dehydrogenase** | **ABB55877.1** | *Tuber borchii* | 345 | 0.0 |
| **5** | **18** | **D5GA95** | **Putative adenosine kinase** | **XP_002487124.1** | *Talaromyces stipitatus ATCC 10500* | 350 | 5e-60 |
| **5** | **19** | **D5G5R4** | **Fructose-bisphosphate aldolase 1** | **EWC47337.1** | *Drechslerella stenobrocha 248* | 360 | 0.0 |
| **6** | **20** | **D5GLV9** | **Inorganic pyrophosphatase** | **XP_008083318.1** | *Glarea lozoyensis ATCC 20868* | 290 | 0.0 |
| **6** | **21** | **D5GAF9** | **Pyridoxine biosynthesis protein pdx1** | **XP_020134599.1** | *Diplodia corticola* | 309 | 0.0 |
| **7** | **23** | **D5GM11** | **Hsp70 chaperone BiP/Kar2, putative** | **XP_002340735.1** | *Talaromyces stipitatus ATCC 10500* | 675 | 0.0 |
| **7** | **24** | **D5G7L2** | **putative oxalate decarboxylase** | **KKY23362.1** | *Diplodia seriata* | 455 | 0.0 |
| **8** | **26** | **D5GI33** | **Peroxiredoxin tsa1** | **XP_020133535.1** | *Diplodia corticola* | 207 | 1e-106 |
| **8** | **27** | **D5GET6** | **60S ribosomal protein L17** | **EWC44437.1** | *Drechslerella stenobrocha 248* | 187 | 3e-97 |
| **9** | **29** | **D5GAV4** | **5-methyltetrahydropteroyltriglutamate-homocysteine S-methyltransferase** | **XP_009157782.1** | *Exophiala dermatitidis NIH/UT8656* | 774 | 0.0 |
| **10** | **30** | **D5G7S8** | **40S ribosomal protein S15** | **XP_002541979.1** | *Uncinocarpus reesii 1704* | 153 | 4e-100 |
| **11** | **33** | **D5GJY5** | **Glyoxal oxidase** | **XP_001937507.1** | *Pyrenophora tritici-repentis Pt-1C-BFP* | 825 | 0.0 |
| **11** | **34** | **D5GK33** | **heat shock protein 60** | **OCL14187.1** | *Glonium stellatum* | 586 | 0.0 |
| **13** | **38** | **D5GNQ7** | **Putative calcium homeostasis protein Regucalcin** | **OOQ84823.1** | *Penicillium brasilianum* | 308 | 1e-54 |
| **13** | **39** | **D5G9M7** | **RNP domain-containing protein** | **EEH09099.1** | *Histoplasma capsulatum G186AR* | 471 | 5e-96 |
| **15** | **42** | **D5G4J8** | **Alcohol oxidase** | **XP_007367253.1** | *Dichomitus squalens LYAD-421 SS1* | 652 | 0.0 |
| **16** | **43** | **D5G8F0** | **Peptide methionine sulfoxide reductase** | **KXH29526.1** | *Colletotrichum simmondsii* | 202 | 8e-96 |
| **17** | **44** | **D5GK33** | **Heat shock protein 60** | **OCL14187.1** | *Glonium stellatum* | 586 | 0.0 |
| **17** | **46** | **D5GGZ0** | **Dihydroxyacetone kinase** | **XP_018076346.1** | *Phialocephala scopiformis* | 591 | 0.0 |
| **17** | **47** | **D5G9B7** | **NADP-dependent malic enzyme** | **OMP81479.1** | *Diplodia seriata* | 581 | 0.0 |
| **18** | **48** | **D5G830** | **Cell wall mannoprotein PIR3** | **ONH70802.1** | *Saccharomyces cerevisiae* | 224 | 1e-32 |
| **19** | **50** | **D5G4P4** | **Cell division control protein 12** | **XP_003174273.1** | *Nannizzia gypsea CBS 118893* | 381 | 0.0 |
| **19** | **51** | **D5GFC4** | **Actin-like protein-like protein 6A** | **XP_018189929.1** | *Xylona heveae TC161* | 470 | 3e-164 |

(**a**) Spot numbers correspond to those reported in Fig. 1; (**b**) numbers correspond to those reported in Table S2; (**c**) UniProtKB accession number, as reported in Table S2; (**d**) RefSeq protein accession number; (**e**) protein size (aa), as reported in the blast output; (**f**) Expect value, from the blast.
