# Supplementary material for: Tuberomics: a molecular profiling for the adaption of edible fungi (Tuber magnatum Pico) to different natural environments
Source: BMC Genomics. 2020 Jan 29;21:90. doi: 10.1186/s12864-020-6522-3 (PMC6988325; doi:10.1186/s12864-020-6522-3)
Supplement: Supplementary file 4 — Additional file 4: Table S4. Summary of sulfur-related proteins identified by nanoLC-ESI-LIT-MS/MS analysis. [file 12864_2020_6522_MOESM4_ESM.docx]

**Table S4: Summary of sulfur-related proteins identified through LC-MS analysis. ^a^ Spot numbers correspond to those reported in Fig. 1**. ^b^ UniProtKB accession number. Red marked proteins were functionally identified after blast analysis (see Additional file 3: Table S3).

| **Spot no. (a)** | **Acc. No. (b)** | **Organism** | **Protein name** | **Common name** |
| --- | --- | --- | --- | --- |
| **1, 3, 13** | **D5GJ78** | *Tuber melanosporum* | **S-adenosylmethionine synthase** | **SAM** |
| **3** | **D5GM51** | *Tuber melanosporum* | **Cystathionine gamma-lyase** | **CTH** |
| **8** | **D5GNP5** | *Tuber melanosporum* | **Adenosylhomocysteinase** | **AHCY** |
| **9** | **D5GAV4** | *Tuber melanosporum* | **5-methyltetrahydropteroyltriglutamate-homocysteine S-methyltransferase** | **MetE** |
| **16** | **D5G8F0** | *Tuber melanosporum* | **Peptide methionine sulfoxide reductase** | **MsrA** |
