# Supplementary material for: Tuberomics: a molecular profiling for the adaption of edible fungi (Tuber magnatum Pico) to different natural environments
Source: BMC Genomics. 2020 Jan 29;21:90. doi: 10.1186/s12864-020-6522-3 (PMC6988325; doi:10.1186/s12864-020-6522-3)
Supplement: Supplementary file 5 — Additional file 5: Table S5. RNA-seq statistics from Vita et al. [33]. [file 12864_2020_6522_MOESM5_ESM.docx]

**Table S5: RNA-seq statistics from Vita *et al.* [33].** Number of quality reads used in the RNA-seq experiment and the resulting mapping rate derived from the alignment process (quasi-mapping mode) against the *de novo T. magnatum* transcriptome composed of 12,367 high confidence protein-coding genes.

| **Samples** | **Number of reads (bp)** | **Mapping rate** |
| --- | --- | --- |
| AL-r1 | 47,501,700 | 58.53 % |
| AL-r2 | 43,989,180 | 59.93 % |
| IS-r1 | 38,457,655 | 51.90 % |
| IS-r2 | 35,403,492 | 57.77 % |
| SM-r1 | 36,192,505 | 57.94 % |
| SM-r2 | 43,341,902 | 53.82 % |
| **Average** | 40,814,406 | 56.64 % |
