# Supplementary figures and images for: Tuberomics: a molecular profiling for the adaption of edible fungi (Tuber magnatum Pico) to different natural environments
Source: BMC Genomics. 2020 Jan 29;21:90. doi: 10.1186/s12864-020-6522-3 (PMC6988325; doi:10.1186/s12864-020-6522-3)

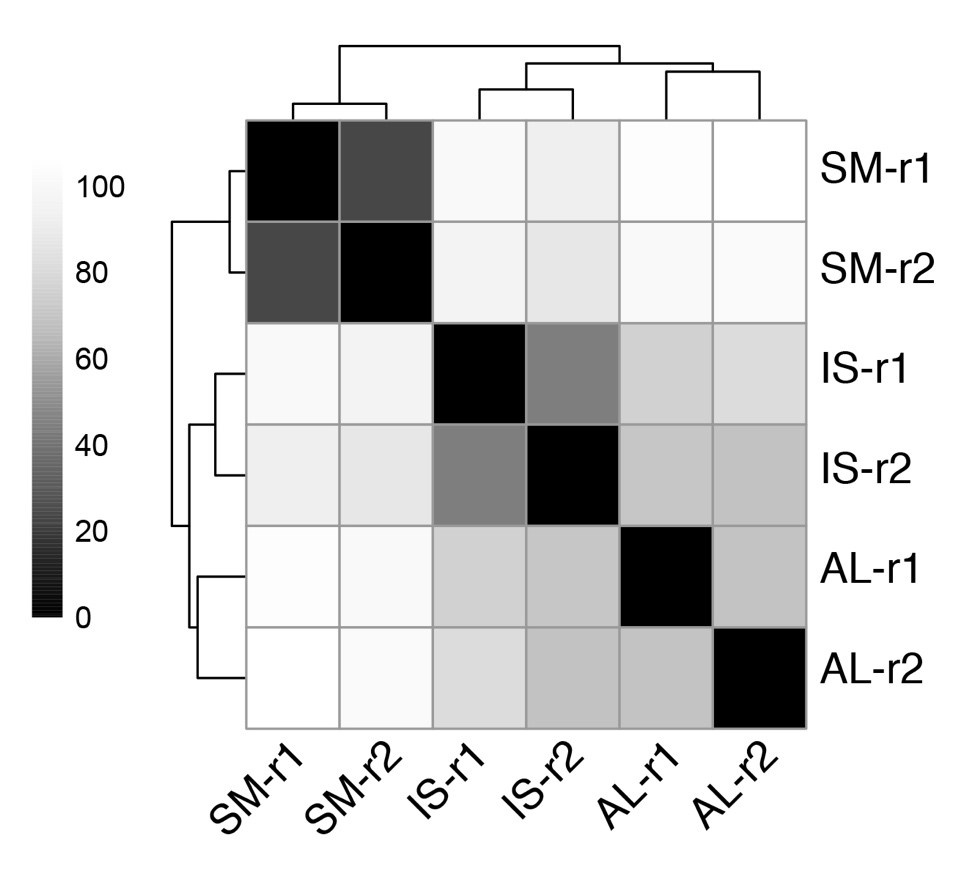

Supplement: Supplementary file 6 — Additional file 6: Figure S1. Euclidean distance among samples. The heat map shows sample to samples distances indicating the strong correlation between biological replicates. The distance matrix was calculated from the normalized expression dataset using the variance-stabilizing transformations function from the Bioconductor package DESeq2. Data were hierarchically clustered based on sample distances. Biological replicates are indicated as r1 (year 2014) and r2 (year 2015). Shades of grey represent different extents of correlation among samples; black represents perfect positive correlation. [file 12864_2020_6522_MOESM6_ESM.jpg]

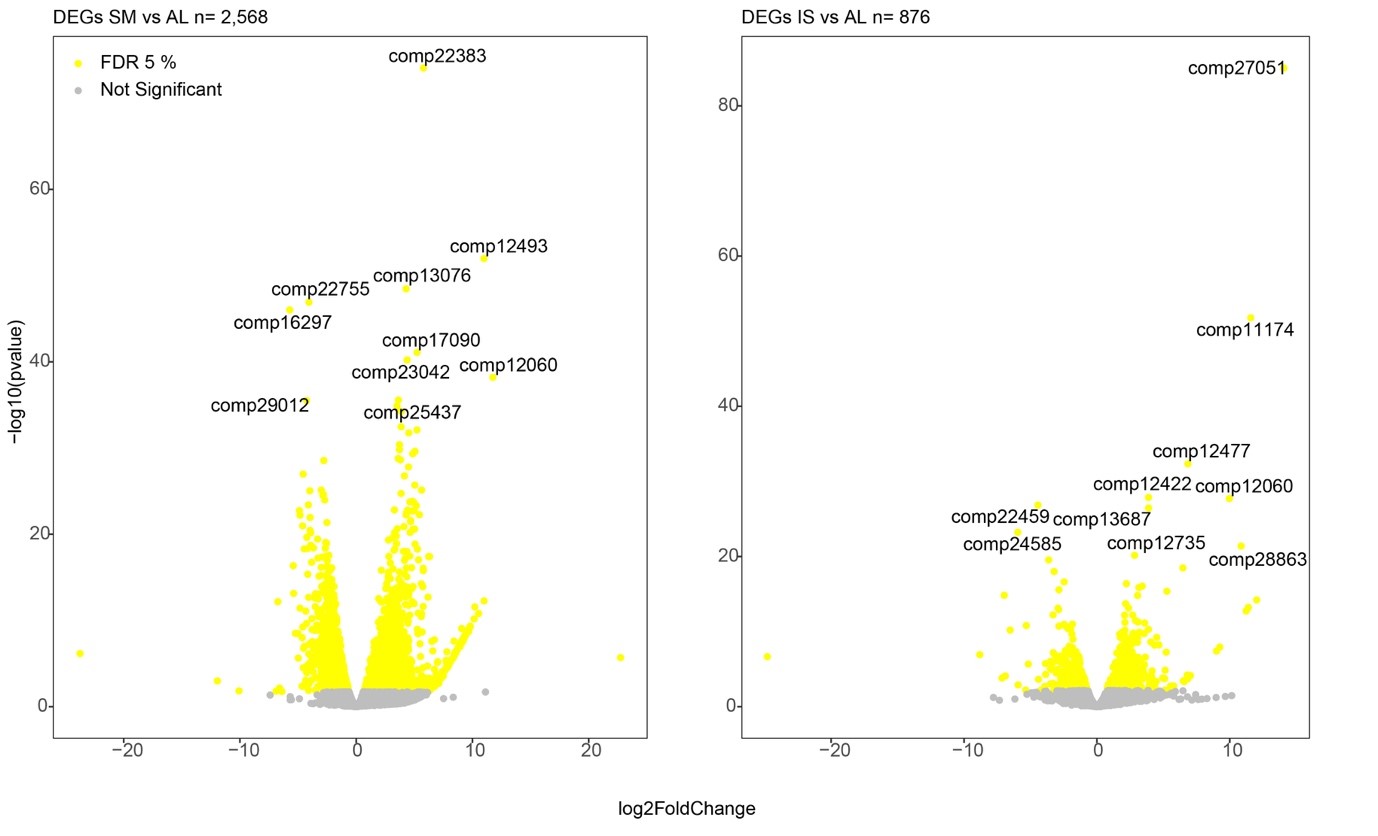

Supplement: Supplementary file 7 — Additional file 7: Figure S2. Volcano plots representing the differentially expressed genes based on RNA-seq data. Pairwise comparisons are shown for San Miniato vs Alba (a) and Isernia vs Alba (b). Yellow dots highlight DEGs selected for |log2 fold change| > 1.5 and FDR < 0.05. [file 12864_2020_6522_MOESM7_ESM.jpg]

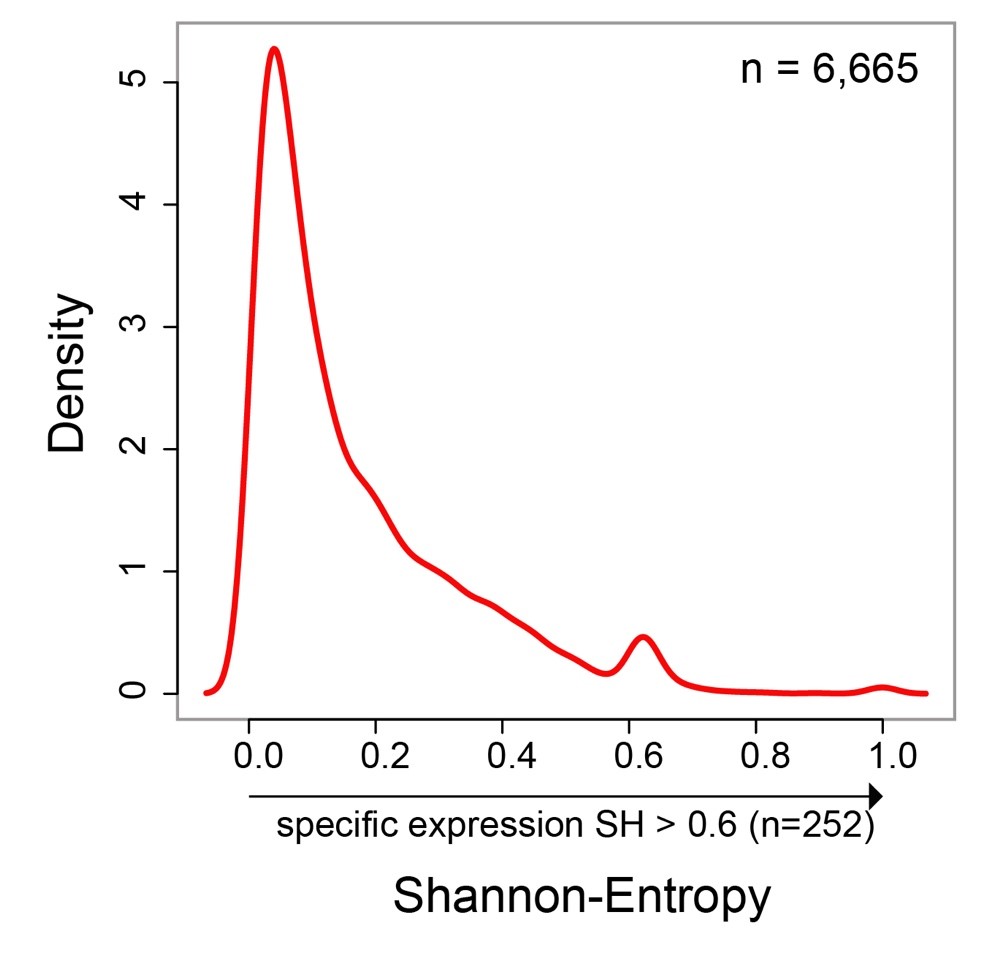

Supplement: Supplementary file 10 — Additional file 10: Figure S3. Sample gene specificity. Shannon entropy (SH) distribution of T. magnatum genes (n = 6665) based on the expression data (in transcripts per million, TPM). A SH coefficient > 0.6 represents the gene-specific expression associated to each T. magnatum ecotype (i.e. geographical accession, see Additional file 11: Data file S1). [file 12864_2020_6522_MOESM10_ESM.jpg]

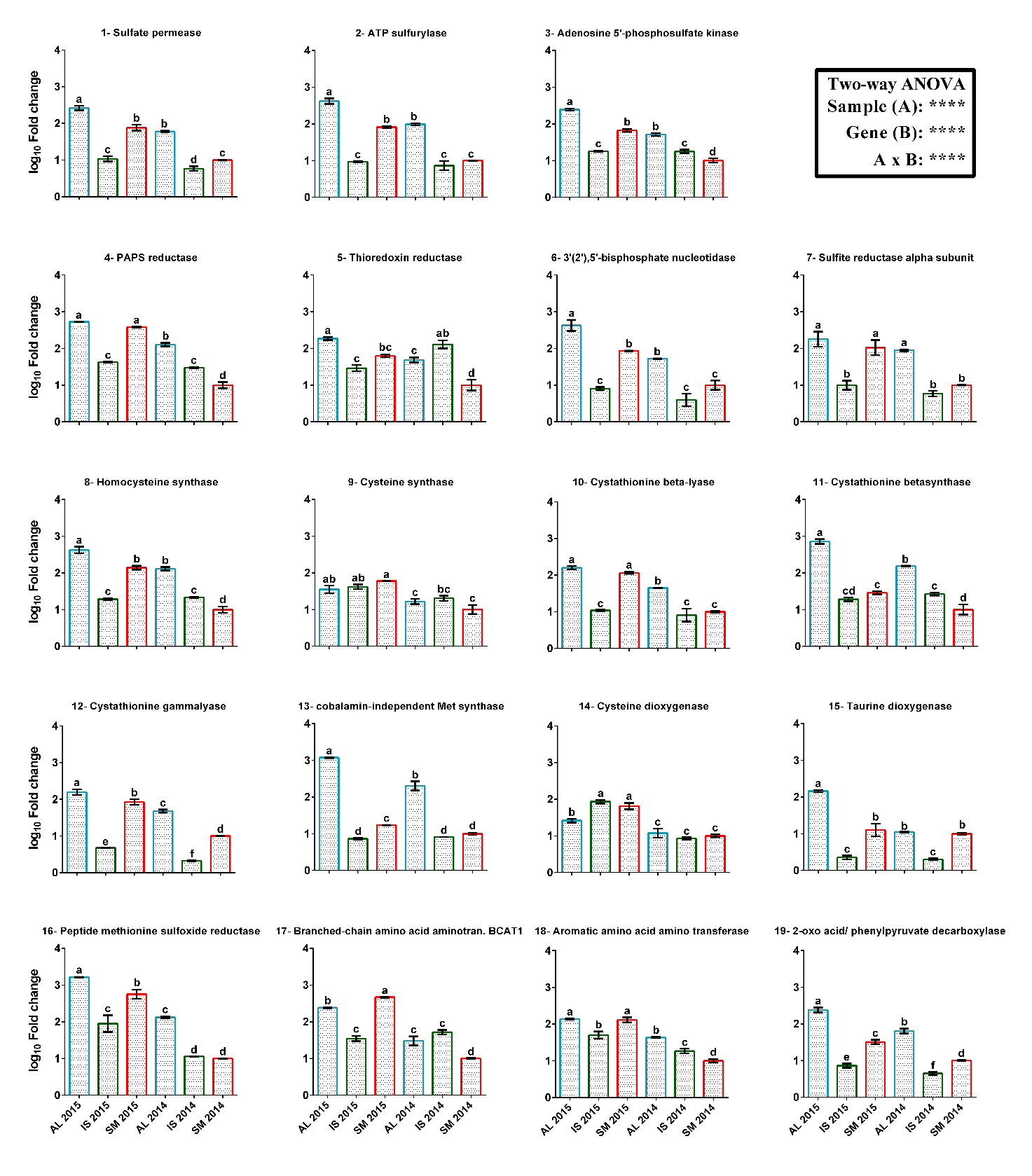

Supplement: Supplementary file 14 — Additional file 14: Figure S4. Relative expression level of the genes selected for the qPCR. Relative levels were expressed, for each gene, as fold change (FC) from the reference sample SM 2014. Data are mean values of transformed data (log10(FC + 1) related to gene expression (n = 4), calculated with the 2-ΔΔCt method [92]. Letters indicate results of Tukey post-hoc test analysis. Sample names correspond to those reported in Table 1. For a reference to RNA-seq transcript IDs, see Additional file 13: Table S9. [file 12864_2020_6522_MOESM14_ESM.tif]
