# Supplementary material for: Tuberomics: a molecular profiling for the adaption of edible fungi (Tuber magnatum Pico) to different natural environments
Source: BMC Genomics. 2020 Jan 29;21:90. doi: 10.1186/s12864-020-6522-3 (PMC6988325; doi:10.1186/s12864-020-6522-3)
Supplement: Supplementary file 8 — Additional file 8: Table S6. Top 100 transcripts related to San Miniato – Alba comparison (differential expression analysis results). [file 12864_2020_6522_MOESM8_ESM.docx]

**Table S6:** **Top 100 transcripts related to San Miniato – Alba comparison (differential expression analysis results).** Upon differential expression analysis, RNA-seq transcripts were ranked according to their adjusted p-value (column “Padj”). For gene annotations refer to F Vita *et al.* [33]. ^a^ “Base Mean” reports the average normalized count values, taken over all samples and divided by size factors; ^b^ “Log2 Fold Change” estimates the size of the effect (i.e. gene expression changes) ^c^ “IfcSE” is the standard error estimate for log_2_ Fold Change. ^d^ “Padj”, p-values adjusted (or FDR, false discovery rate) for multiple testing using Benjamini-Hochberg method.

| **Gene name** | **base Mean^a^** | **log_2_ Fold Change^b^** | **lfcSE^c^** | **stat** | **p-value** | **Padj (FDR)^d^** |
| --- | --- | --- | --- | --- | --- | --- |
| comp22383 | 2225.11 | 5.79 | 0.32 | 18.30 | 8.22E-75 | 5.48E-71 |
| comp12493 | 2910.37 | 10.99 | 0.72 | 15.28 | 1.05E-52 | 3.50E-49 |
| comp13076 | 1995.93 | 4.30 | 0.29 | 14.74 | 3.39E-49 | 7.54E-46 |
| comp22755 | 11911.41 | -4.06 | 0.28 | -14.50 | 1.22E-47 | 2.03E-44 |
| comp16297 | 1374.55 | -5.72 | 0.40 | -14.36 | 9.46E-47 | 1.26E-43 |
| comp17090 | 632.80 | 5.24 | 0.39 | 13.55 | 8.18E-42 | 9.09E-39 |
| comp23042 | 1232.50 | 4.38 | 0.33 | 13.40 | 6.09E-41 | 5.80E-38 |
| comp12060 | 3269.67 | 11.76 | 0.90 | 13.05 | 6.24E-39 | 5.20E-36 |
| comp25437 | 1760.44 | 3.63 | 0.29 | 12.58 | 2.68E-36 | 1.98E-33 |
| comp29012 | 18258.05 | -4.27 | 0.34 | -12.57 | 3.08E-36 | 2.05E-33 |
| comp29578 | 1161.61 | 3.51 | 0.28 | 12.45 | 1.36E-35 | 8.24E-33 |
| comp29595 | 677.54 | 3.88 | 0.31 | 12.34 | 5.85E-35 | 3.25E-32 |
| comp29072 | 14067.00 | 3.86 | 0.32 | 12.00 | 3.41E-33 | 1.75E-30 |
| comp15296 | 424.15 | 5.23 | 0.44 | 11.93 | 8.00E-33 | 3.81E-30 |
| comp16201 | 2545.42 | 4.53 | 0.38 | 11.87 | 1.73E-32 | 7.70E-30 |
| comp26573 | 1225.20 | 3.73 | 0.32 | 11.60 | 4.11E-31 | 1.71E-28 |
| comp30530 | 2297.70 | 3.73 | 0.32 | 11.48 | 1.58E-30 | 6.21E-28 |
| comp29860 | 1097.48 | 5.06 | 0.44 | 11.45 | 2.44E-30 | 9.02E-28 |
| comp25422 | 379.30 | 4.86 | 0.43 | 11.39 | 4.66E-30 | 1.64E-27 |
| comp28280 | 515.86 | 3.61 | 0.32 | 11.28 | 1.64E-29 | 5.46E-27 |
| comp30051 | 3208.37 | 3.81 | 0.34 | 11.25 | 2.36E-29 | 7.50E-27 |
| comp12390 | 78184.81 | -2.79 | 0.25 | -11.23 | 2.83E-29 | 8.58E-27 |
| comp30732 | 408.39 | 4.50 | 0.41 | 11.08 | 1.57E-28 | 4.56E-26 |
| comp22912 | 25074.76 | -4.56 | 0.42 | -10.91 | 1.05E-27 | 2.92E-25 |
| comp22026 | 541.95 | 4.15 | 0.38 | 10.87 | 1.69E-27 | 4.51E-25 |
| comp30674 | 915.53 | 5.04 | 0.47 | 10.64 | 1.98E-26 | 5.07E-24 |
| comp23224 | 5999.34 | -3.00 | 0.29 | -10.51 | 7.51E-26 | 1.79E-23 |
| comp25771 | 716.76 | 5.63 | 0.54 | 10.51 | 7.48E-26 | 1.79E-23 |
| comp12535 | 6945.40 | -3.99 | 0.38 | -10.49 | 9.16E-26 | 2.11E-23 |
| comp12499 | 2662.55 | 3.86 | 0.37 | 10.43 | 1.85E-25 | 4.11E-23 |
| comp29091 | 13572.42 | -2.82 | 0.27 | -10.40 | 2.46E-25 | 5.30E-23 |
| comp17497 | 3823.72 | -2.87 | 0.28 | -10.38 | 3.00E-25 | 6.24E-23 |
| comp12445 | 73515.67 | -2.71 | 0.26 | -10.26 | 1.06E-24 | 2.14E-22 |
| comp37140 | 255.23 | 4.84 | 0.47 | 10.23 | 1.52E-24 | 2.98E-22 |
| comp26697 | 241.82 | 4.62 | 0.45 | 10.21 | 1.79E-24 | 3.40E-22 |
| comp27645 | 686.30 | 4.95 | 0.48 | 10.20 | 1.96E-24 | 3.63E-22 |
| comp17507 | 2201.54 | -4.12 | 0.41 | -10.13 | 3.95E-24 | 7.12E-22 |
| comp28946 | 221.92 | 5.17 | 0.51 | 10.11 | 4.86E-24 | 8.53E-22 |
| comp28757 | 692.25 | 3.29 | 0.33 | 10.00 | 1.51E-23 | 2.58E-21 |
| comp31328 | 428.99 | 4.41 | 0.44 | 9.99 | 1.62E-23 | 2.70E-21 |
| comp23192 | 11279.45 | -4.89 | 0.49 | -9.98 | 1.82E-23 | 2.95E-21 |
| comp27904 | 163.74 | 5.00 | 0.50 | 9.98 | 1.94E-23 | 3.08E-21 |
| comp27715 | 261.69 | 4.79 | 0.48 | 9.97 | 2.04E-23 | 3.16E-21 |
| comp10407 | 342.52 | 4.34 | 0.44 | 9.94 | 2.82E-23 | 4.26E-21 |
| comp26392 | 176.11 | 5.44 | 0.55 | 9.87 | 5.46E-23 | 8.08E-21 |
| comp27115 | 237.28 | 4.29 | 0.44 | 9.87 | 5.80E-23 | 8.40E-21 |
| comp23710 | 1324.69 | -4.83 | 0.49 | -9.86 | 6.30E-23 | 8.94E-21 |
| comp22459 | 3360.47 | -3.96 | 0.40 | -9.80 | 1.14E-22 | 1.58E-20 |
| comp21620 | 543.84 | 4.69 | 0.48 | 9.72 | 2.55E-22 | 3.47E-20 |
| comp24274 | 475.82 | 4.68 | 0.48 | 9.69 | 3.45E-22 | 4.59E-20 |
| comp24246 | 3127.22 | -2.52 | 0.26 | -9.66 | 4.42E-22 | 5.78E-20 |
| comp23154 | 13957.14 | -4.61 | 0.48 | -9.57 | 1.07E-21 | 1.37E-19 |
| comp16092 | 382.09 | 4.11 | 0.43 | 9.55 | 1.30E-21 | 1.63E-19 |
| comp18332 | 646.47 | 3.54 | 0.37 | 9.49 | 2.34E-21 | 2.89E-19 |
| comp26948 | 165.66 | 5.03 | 0.53 | 9.48 | 2.51E-21 | 3.04E-19 |
| comp14668 | 171.12 | 4.89 | 0.52 | 9.48 | 2.63E-21 | 3.13E-19 |
| comp24453 | 10450.29 | -3.96 | 0.42 | -9.45 | 3.55E-21 | 4.15E-19 |
| comp16422 | 1198.14 | 4.64 | 0.49 | 9.39 | 6.18E-21 | 7.11E-19 |
| comp17601 | 506.51 | 3.31 | 0.35 | 9.38 | 6.29E-21 | 7.11E-19 |
| comp16167 | 718.43 | -3.92 | 0.42 | -9.37 | 6.93E-21 | 7.69E-19 |
| comp13253 | 1163.26 | 3.45 | 0.37 | 9.28 | 1.63E-20 | 1.78E-18 |
| comp13118 | 29400.90 | -4.25 | 0.46 | -9.25 | 2.33E-20 | 2.50E-18 |
| comp13742 | 672.04 | 3.14 | 0.34 | 9.24 | 2.56E-20 | 2.71E-18 |
| comp29201 | 3536.84 | -3.33 | 0.36 | -9.20 | 3.62E-20 | 3.77E-18 |
| comp28780 | 476.36 | 2.77 | 0.30 | 9.17 | 4.64E-20 | 4.76E-18 |
| comp22771 | 24595.43 | -2.63 | 0.29 | -9.10 | 9.05E-20 | 9.14E-18 |
| comp32765 | 1577.27 | -2.55 | 0.28 | -9.08 | 1.11E-19 | 1.11E-17 |
| comp24559 | 2044.00 | 3.47 | 0.38 | 9.07 | 1.21E-19 | 1.18E-17 |
| comp27514 | 140.79 | 5.06 | 0.56 | 9.05 | 1.39E-19 | 1.34E-17 |
| comp28272 | 78428.27 | -3.90 | 0.43 | -9.02 | 1.93E-19 | 1.84E-17 |
| comp16391 | 5660.62 | -3.67 | 0.41 | -8.94 | 3.79E-19 | 3.56E-17 |
| comp29271 | 8379.13 | -2.66 | 0.30 | -8.93 | 4.19E-19 | 3.88E-17 |
| comp27391 | 3865.53 | -4.15 | 0.46 | -8.93 | 4.30E-19 | 3.93E-17 |
| comp16407 | 29237.61 | -4.47 | 0.50 | -8.91 | 5.01E-19 | 4.51E-17 |
| comp12091 | 1686.51 | 3.32 | 0.37 | 8.91 | 5.27E-19 | 4.66E-17 |
| comp7120 | 149.62 | 5.27 | 0.59 | 8.91 | 5.32E-19 | 4.66E-17 |
| comp16171 | 238.70 | 3.26 | 0.37 | 8.87 | 7.10E-19 | 6.15E-17 |
| comp23251 | 2021.40 | 4.07 | 0.46 | 8.84 | 9.53E-19 | 8.14E-17 |
| comp28109 | 170.39 | 4.36 | 0.49 | 8.84 | 9.87E-19 | 8.33E-17 |
| comp25731 | 4174.85 | -2.35 | 0.27 | -8.72 | 2.80E-18 | 2.33E-16 |
| comp17138 | 142.81 | 4.62 | 0.53 | 8.70 | 3.37E-18 | 2.77E-16 |
| comp16260 | 6174.85 | -2.56 | 0.29 | -8.70 | 3.42E-18 | 2.78E-16 |
| comp25700 | 862.95 | 2.81 | 0.32 | 8.69 | 3.66E-18 | 2.94E-16 |
| comp26046 | 195.73 | 6.23 | 0.72 | 8.68 | 3.87E-18 | 3.07E-16 |
| comp44116 | 157.68 | 6.31 | 0.73 | 8.68 | 3.91E-18 | 3.07E-16 |
| comp23174 | 9938.99 | -2.91 | 0.34 | -8.67 | 4.37E-18 | 3.39E-16 |
| comp26128 | 11037.05 | -3.29 | 0.38 | -8.64 | 5.79E-18 | 4.44E-16 |
| comp17252 | 1829.27 | 4.22 | 0.49 | 8.63 | 6.40E-18 | 4.85E-16 |
| comp28785 | 154.36 | 5.36 | 0.62 | 8.58 | 9.20E-18 | 6.89E-16 |
| comp29717 | 1629.57 | 4.15 | 0.48 | 8.57 | 1.04E-17 | 7.73E-16 |
| comp12209 | 5263.07 | -2.49 | 0.29 | -8.56 | 1.13E-17 | 8.29E-16 |
| comp22883 | 471794.28 | -3.83 | 0.45 | -8.51 | 1.77E-17 | 1.28E-15 |
| comp38083 | 339.20 | 2.94 | 0.35 | 8.48 | 2.19E-17 | 1.57E-15 |
| comp27457 | 535.67 | -5.42 | 0.64 | -8.40 | 4.43E-17 | 3.14E-15 |
| comp28184 | 4041.57 | -2.48 | 0.30 | -8.38 | 5.32E-17 | 3.73E-15 |
| comp12500 | 12748.47 | -3.01 | 0.36 | -8.35 | 6.89E-17 | 4.77E-15 |
| comp29849 | 2461.21 | -2.48 | 0.30 | -8.35 | 6.94E-17 | 4.77E-15 |
| comp27247 | 99.88 | 4.76 | 0.57 | 8.34 | 7.65E-17 | 5.21E-15 |
| comp16728 | 6367.15 | -2.10 | 0.25 | -8.33 | 7.81E-17 | 5.26E-15 |
| comp23604 | 253.25 | 4.31 | 0.52 | 8.33 | 8.32E-17 | 5.54E-15 |
