# Supplementary material for: Tuberomics: a molecular profiling for the adaption of edible fungi (Tuber magnatum Pico) to different natural environments
Source: BMC Genomics. 2020 Jan 29;21:90. doi: 10.1186/s12864-020-6522-3 (PMC6988325; doi:10.1186/s12864-020-6522-3)
Supplement: Supplementary file 9 — Additional file 9: Table S7. Top 100 transcripts related to Isernia – Alba comparison (differential expression analysis results). [file 12864_2020_6522_MOESM9_ESM.docx]

**Table S7: Top 100 transcripts related to Isernia – Alba comparison comparison (differential expression analysis results).** Upon differential expression analysis, RNA-seq transcripts were ranked according to their adjusted p-value (column “Padj”). For gene annotations refer to Vita *et al.* [33]. Gene names and column contents are the same described in Additional file 8: Table S6.

| **Gene name** | **base Mean^a^** | **log_2_ Fold Change^b^** | **lfcSE^c^** | **stat** | **p-value** | **Padj (FDR)^d^** |
| --- | --- | --- | --- | --- | --- | --- |
| comp27051 | 22916.43 | 14.08 | 0.72 | 19.63 | 8.97E-86 | 5.63E-82 |
| comp11174 | 2852.17 | 11.58 | 0.76 | 15.25 | 1.63E-52 | 5.13E-49 |
| comp12477 | 10157.83 | 6.85 | 0.57 | 11.98 | 4.61E-33 | 9.64E-30 |
| comp12422 | 133147.22 | 3.88 | 0.35 | 11.09 | 1.38E-28 | 2.17E-25 |
| comp12060 | 3269.67 | 9.97 | 0.90 | 11.06 | 1.93E-28 | 2.42E-25 |
| comp22459 | 3360.47 | -4.43 | 0.41 | -10.88 | 1.44E-27 | 1.51E-24 |
| comp13687 | 5699.24 | 3.89 | 0.36 | 10.80 | 3.53E-27 | 3.17E-24 |
| comp24585 | 9906.25 | -5.95 | 0.59 | -10.09 | 6.08E-24 | 4.77E-21 |
| comp28863 | 469.33 | 10.85 | 1.12 | 9.67 | 4.07E-22 | 2.84E-19 |
| comp12735 | 3185.23 | 2.83 | 0.30 | 9.37 | 7.09E-21 | 4.45E-18 |
| comp16297 | 1374.55 | -3.63 | 0.39 | -9.23 | 2.83E-20 | 1.61E-17 |
| comp12493 | 2910.37 | 6.47 | 0.72 | 8.96 | 3.35E-19 | 1.75E-16 |
| comp26176 | 5229.30 | -3.22 | 0.36 | -8.83 | 1.01E-18 | 4.88E-16 |
| comp29839 | 4222.26 | -2.47 | 0.29 | -8.47 | 2.41E-17 | 1.08E-14 |
| comp16440 | 5440.37 | 2.22 | 0.26 | 8.41 | 4.19E-17 | 1.75E-14 |
| comp30732 | 408.39 | 3.41 | 0.41 | 8.32 | 8.97E-17 | 3.52E-14 |
| comp16201 | 2545.42 | 3.16 | 0.38 | 8.27 | 1.39E-16 | 5.12E-14 |
| comp16666 | 2224.39 | -2.86 | 0.35 | -8.19 | 2.72E-16 | 9.49E-14 |
| comp28157 | 2257.96 | 5.27 | 0.65 | 8.13 | 4.29E-16 | 1.42E-13 |
| comp28568 | 4390.54 | -6.97 | 0.87 | -7.98 | 1.48E-15 | 4.65E-13 |
| comp16409 | 15589.45 | 3.06 | 0.38 | 7.97 | 1.57E-15 | 4.70E-13 |
| comp28893 | 294.92 | 12.02 | 1.54 | 7.80 | 6.09E-15 | 1.74E-12 |
| comp29694 | 2165.25 | 2.16 | 0.28 | 7.65 | 2.00E-14 | 5.45E-12 |
| comp28921 | 190.80 | 11.40 | 1.52 | 7.51 | 6.10E-14 | 1.59E-11 |
| comp28920 | 190.19 | 11.39 | 1.52 | 7.49 | 6.82E-14 | 1.71E-11 |
| comp29595 | 677.54 | 2.39 | 0.32 | 7.48 | 7.27E-14 | 1.75E-11 |
| comp28167 | 2521.24 | -2.92 | 0.39 | -7.47 | 7.78E-14 | 1.81E-11 |
| comp16679 | 5250.81 | -2.87 | 0.39 | -7.42 | 1.20E-13 | 2.70E-11 |
| comp28740 | 169.84 | 11.23 | 1.53 | 7.36 | 1.81E-13 | 3.92E-11 |
| comp27588 | 284.88 | -3.30 | 0.46 | -7.20 | 6.07E-13 | 1.27E-10 |
| comp25437 | 1760.44 | 2.09 | 0.29 | 7.19 | 6.54E-13 | 1.28E-10 |
| comp28707 | 5233.24 | 2.69 | 0.37 | 7.19 | 6.52E-13 | 1.28E-10 |
| comp23458 | 662.80 | 2.90 | 0.42 | 6.96 | 3.36E-12 | 6.38E-10 |
| comp15296 | 424.15 | 3.07 | 0.44 | 6.90 | 5.08E-12 | 9.37E-10 |
| comp16858 | 202.92 | 3.10 | 0.45 | 6.89 | 5.49E-12 | 9.85E-10 |
| comp22924 | 14531.41 | 2.11 | 0.31 | 6.88 | 6.07E-12 | 1.06E-09 |
| comp14668 | 171.12 | 3.59 | 0.52 | 6.85 | 7.30E-12 | 1.24E-09 |
| comp25731 | 4174.85 | -1.84 | 0.27 | -6.80 | 1.03E-11 | 1.70E-09 |
| comp27971 | 10696.95 | -2.47 | 0.36 | -6.79 | 1.11E-11 | 1.79E-09 |
| comp27105 | 50966.55 | -5.31 | 0.79 | -6.74 | 1.58E-11 | 2.48E-09 |
| comp28992 | 12695.06 | -2.85 | 0.42 | -6.71 | 1.89E-11 | 2.89E-09 |
| comp25666 | 3064.71 | -2.33 | 0.35 | -6.71 | 2.01E-11 | 3.00E-09 |
| comp22775 | 6781.95 | -2.05 | 0.31 | -6.64 | 3.07E-11 | 4.48E-09 |
| comp27404 | 921.28 | -2.18 | 0.33 | -6.61 | 3.91E-11 | 5.57E-09 |
| comp16552 | 646.75 | -1.98 | 0.30 | -6.57 | 4.98E-11 | 6.95E-09 |
| comp54175 | 86.99 | 3.88 | 0.59 | 6.57 | 5.11E-11 | 6.97E-09 |
| comp22103 | 319.13 | -6.53 | 1.00 | -6.54 | 6.30E-11 | 8.41E-09 |
| comp12986 | 26888.62 | 2.11 | 0.32 | 6.53 | 6.48E-11 | 8.47E-09 |
| comp13076 | 1995.93 | 1.89 | 0.29 | 6.43 | 1.24E-10 | 1.57E-08 |
| comp26038 | 462.73 | 3.17 | 0.49 | 6.43 | 1.25E-10 | 1.57E-08 |
| comp27904 | 163.74 | 3.28 | 0.51 | 6.41 | 1.49E-10 | 1.84E-08 |
| comp29113 | 8877.48 | 2.74 | 0.43 | 6.40 | 1.59E-10 | 1.92E-08 |
| comp14554 | 104.31 | 3.56 | 0.56 | 6.37 | 1.83E-10 | 2.17E-08 |
| comp15259 | 10861.64 | -1.88 | 0.30 | -6.35 | 2.15E-10 | 2.50E-08 |
| comp12703 | 2570.11 | 1.72 | 0.27 | 6.35 | 2.19E-10 | 2.50E-08 |
| comp25611 | 5010.18 | 2.70 | 0.43 | 6.33 | 2.43E-10 | 2.73E-08 |
| comp30530 | 2297.70 | 2.06 | 0.33 | 6.33 | 2.49E-10 | 2.74E-08 |
| comp28072 | 115.56 | 4.50 | 0.73 | 6.19 | 5.93E-10 | 6.39E-08 |
| comp29243 | 3957.43 | 1.76 | 0.28 | 6.19 | 6.01E-10 | 6.39E-08 |
| comp25329 | 159.19 | 3.05 | 0.50 | 6.16 | 7.25E-10 | 7.59E-08 |
| comp29711 | 2714.58 | 1.89 | 0.31 | 6.15 | 7.78E-10 | 8.01E-08 |
| comp12325 | 479.50 | 2.43 | 0.40 | 6.11 | 9.74E-10 | 9.86E-08 |
| comp28184 | 4041.57 | -1.81 | 0.30 | -6.10 | 1.05E-09 | 1.05E-07 |
| comp26854 | 346.85 | 2.63 | 0.43 | 6.08 | 1.19E-09 | 1.17E-07 |
| comp28691 | 79.86 | 4.06 | 0.68 | 5.97 | 2.43E-09 | 2.35E-07 |
| comp31258 | 495.55 | 2.15 | 0.36 | 5.96 | 2.57E-09 | 2.45E-07 |
| comp28239 | 394.12 | 3.06 | 0.51 | 5.95 | 2.73E-09 | 2.56E-07 |
| comp17601 | 506.51 | 2.12 | 0.36 | 5.94 | 2.79E-09 | 2.57E-07 |
| comp13600 | 499.96 | 2.27 | 0.38 | 5.94 | 2.84E-09 | 2.58E-07 |
| comp17161 | 217.12 | 2.69 | 0.46 | 5.89 | 3.90E-09 | 3.50E-07 |
| comp27136 | 4720.91 | 2.53 | 0.43 | 5.87 | 4.36E-09 | 3.86E-07 |
| comp15973 | 1016.97 | 2.32 | 0.40 | 5.85 | 4.87E-09 | 4.25E-07 |
| comp23519 | 2424.72 | 4.67 | 0.80 | 5.82 | 5.96E-09 | 5.12E-07 |
| comp14562 | 6288.78 | 4.34 | 0.75 | 5.81 | 6.22E-09 | 5.28E-07 |
| comp22681 | 2880.97 | 1.86 | 0.32 | 5.76 | 8.44E-09 | 7.03E-07 |
| comp27994 | 287.13 | 2.44 | 0.42 | 5.76 | 8.51E-09 | 7.03E-07 |
| comp21792 | 978.96 | -2.08 | 0.36 | -5.75 | 8.75E-09 | 7.13E-07 |
| comp28729 | 43.16 | 9.25 | 1.62 | 5.70 | 1.18E-08 | 9.46E-07 |
| comp11599 | 887.10 | -1.85 | 0.32 | -5.69 | 1.29E-08 | 1.03E-06 |
| comp12307 | 17374.14 | 1.73 | 0.30 | 5.67 | 1.43E-08 | 1.12E-06 |
| comp11366 | 515.56 | 2.01 | 0.35 | 5.66 | 1.51E-08 | 1.17E-06 |
| comp27948 | 296.73 | -1.86 | 0.33 | -5.61 | 2.04E-08 | 1.56E-06 |
| comp10459 | 1623.15 | 2.47 | 0.44 | 5.57 | 2.50E-08 | 1.89E-06 |
| comp28223 | 540.31 | 2.37 | 0.43 | 5.55 | 2.82E-08 | 2.11E-06 |
| comp8184 | 148.20 | 2.53 | 0.46 | 5.54 | 3.05E-08 | 2.25E-06 |
| comp31585 | 1799.88 | 2.51 | 0.45 | 5.53 | 3.25E-08 | 2.37E-06 |
| comp28345 | 908.64 | -2.37 | 0.43 | -5.52 | 3.36E-08 | 2.43E-06 |
| comp29095 | 11648.84 | -2.14 | 0.39 | -5.52 | 3.47E-08 | 2.48E-06 |
| comp27322 | 37.97 | 9.00 | 1.64 | 5.50 | 3.83E-08 | 2.70E-06 |
| comp21920 | 1002.06 | -1.59 | 0.29 | -5.49 | 4.08E-08 | 2.85E-06 |
| comp29950 | 3878.12 | -1.71 | 0.31 | -5.47 | 4.42E-08 | 3.05E-06 |
| comp22696 | 3838.96 | -1.63 | 0.30 | -5.44 | 5.20E-08 | 3.55E-06 |
| comp23045 | 2774.27 | -1.85 | 0.34 | -5.44 | 5.34E-08 | 3.57E-06 |
| comp24836 | 134.85 | 2.65 | 0.49 | 5.44 | 5.29E-08 | 3.57E-06 |
| comp27902 | 365.50 | 1.84 | 0.34 | 5.43 | 5.48E-08 | 3.62E-06 |
| comp25295 | 34.11 | 5.21 | 0.96 | 5.43 | 5.68E-08 | 3.71E-06 |
| comp26093 | 1886.11 | 2.15 | 0.40 | 5.43 | 5.75E-08 | 3.72E-06 |
| comp21849 | 1343.72 | -3.28 | 0.61 | -5.40 | 6.50E-08 | 4.16E-06 |
| comp30051 | 3208.37 | 1.83 | 0.34 | 5.40 | 6.72E-08 | 4.26E-06 |
| comp27949 | 2533.59 | 1.50 | 0.28 | 5.39 | 7.11E-08 | 4.46E-06 |
