# Supplementary material for: Tuberomics: a molecular profiling for the adaption of edible fungi (Tuber magnatum Pico) to different natural environments
Source: BMC Genomics. 2020 Jan 29;21:90. doi: 10.1186/s12864-020-6522-3 (PMC6988325; doi:10.1186/s12864-020-6522-3)
Supplement: Supplementary file 12 — Additional file 12: Table S8. Sample-specific expression of T. magnatum genes involved in sulfur metabolism. [file 12864_2020_6522_MOESM12_ESM.docx]

**Table S8: Sample-specific expression of *T. magnatum* genes involved in sulfur metabolism. Transcripts belonging to sulfur-related Gene Ontology “biological process” categories (http://amigo.geneontology.org/) are shown.** The following GO-IDs and associated terms were considered for the analysis: **GO:0000096**, sulfur amino acid metabolic process; **GO:0000097**, sulfur amino acid biosynthetic process; **GO:0000098**, sulfur amino acid catabolic process; **GO:0006790**, sulfur compound metabolic process; **GO:0009086**, methionine biosynthetic process; **GO:0010134**, sulfate assimilation via adenylyl sulfate reduction; **GO:0019343**, cysteine biosynthetic process via cystathionine; **GO:0019344**, cysteine biosynthetic process; **GO:0019346**, transsulfuration; **GO:0019379**, sulfate assimilation; **GO:0070814**, hydrogen sulfide biosynthetic process. The table shows the statistics of the differential expression analysis in the two comparisons SM vs AL and IS vs AL). Red color marks genes that were further analyzed through qPCR analysis. Gene names and column contents are the same described in Additional files 8 and 9: Tables S6, S7.

|  | **SM vs AL** | | | | | | **IS vs AL** | | | | | |
| --- | --- | --- | --- | --- | --- | --- | --- | --- | --- | --- | --- | --- |
| **Gene** | **base Mean^a^** | **log_2_ Fold Change^b^** | **lfcSE^c^** | **stat** | **p-value** | **Padj^d^** | **base Mean^a^** | **log_2_ Fold Change^b^** | **lfcSE^c^** | **stat** | **p-value** | **Padj^d^** |
| **comp12185** | 3762.66 | **-1.93** | 0.33 | -5.88 | 4.05E-09 | 6.98E-08 | 3762.66 | **0.13** | 0.33 | 0.39 | 6.94E-01 | 8.58E-01 |
| **comp12359** | 19298.02 | **-2.36** | 0.42 | -5.62 | 1.89E-08 | 2.76E-07 | 19298.02 | **0.07** | 0.42 | 0.16 | 8.74E-01 | 9.60E-01 |
| **comp12411** | 101922.63 | **-2.30** | 0.47 | -4.90 | 9.60E-07 | 8.85E-06 | 101922.63 | **-2.32** | 0.47 | -4.95 | 7.56E-07 | 3.44E-05 |
| **comp13965** | 3442.83 | **-1.52** | 0.32 | -4.72 | 2.41E-06 | 2.02E-05 | 3442.83 | **-0.76** | 0.32 | -2.37 | 1.79E-02 | 9.55E-02 |
| **comp16500** | 44529.61 | **-0.72** | 0.35 | -2.04 | 4.15E-02 | 9.37E-02 | 44529.61 | **-0.56** | 0.35 | -1.59 | 1.11E-01 | 3.04E-01 |
| **comp16628** | 54667.23 | **-2.53** | 0.40 | -6.26 | 3.74E-10 | 8.09E-09 | 54667.23 | **-0.26** | 0.40 | -0.64 | 5.22E-01 | 7.40E-01 |
| **comp18325** | 7391.85 | **-1.70** | 0.29 | -5.82 | 6.03E-09 | 1.00E-07 | 7391.85 | **-0.38** | 0.29 | -1.29 | 1.96E-01 | 4.26E-01 |
| **comp22081** | 227.63 | **2.77** | 0.59 | 4.65 | 3.33E-06 | 2.70E-05 | 227.63 | **2.55** | 0.60 | 4.27 | 1.96E-05 | 5.23E-04 |
| **comp22775** | 6781.95 | **-1.58** | 0.31 | -5.15 | 2.66E-07 | 2.85E-06 | 6781.95 | **-2.05** | 0.31 | -6.64 | 3.07E-11 | 4.48E-09 |
| **comp22876** | 362568.17 | **-1.91** | 0.61 | -3.12 | 1.79E-03 | 6.97E-03 | 362568.17 | **-0.91** | 0.61 | -1.49 | 1.37E-01 | 3.45E-01 |
| **comp22883** | 471794.28 | **-3.83** | 0.45 | -8.51 | 1.77E-17 | 1.28E-15 | 471794.28 | **0.24** | 0.45 | 0.53 | 5.94E-01 | 7.93E-01 |
| **comp22902** | 205855.27 | **-3.01** | 0.44 | -6.76 | 1.37E-11 | 3.87E-10 | 205855.27 | **-0.37** | 0.44 | -0.83 | 4.06E-01 | 6.52E-01 |
| **comp23079** | 118977.60 | **-2.12** | 0.66 | -3.23 | 1.25E-03 | 5.09E-03 | 118977.60 | **1.41** | 0.66 | 2.15 | 3.17E-02 | 1.37E-01 |
| **comp24266** | 228006.89 | **0.22** | 0.85 | 0.26 | 7.98E-01 | 8.60E-01 | 228006.89 | **0.37** | 0.85 | 0.44 | 6.58E-01 | 8.36E-01 |
| **comp24484** | 29092.78 | **-1.34** | 0.27 | -5.00 | 5.61E-07 | 5.44E-06 | 29092.78 | **-0.31** | 0.27 | -1.15 | 2.49E-01 | 4.96E-01 |
| **comp24488** | 1563.76 | **-0.62** | 0.27 | -2.28 | 2.29E-02 | 5.74E-02 | 1563.76 | **-0.44** | 0.27 | -1.61 | 1.08E-01 | 2.98E-01 |
| **comp26862** | 3131.74 | **-1.70** | 0.66 | -2.58 | 9.96E-03 | 2.92E-02 | 3131.74 | **-0.17** | 0.66 | -0.26 | 7.91E-01 | 9.11E-01 |
| **comp27593** | 236943.78 | **-1.07** | 0.29 | -3.69 | 2.27E-04 | 1.14E-03 | 236943.78 | **0.17** | 0.29 | 0.59 | 5.55E-01 | 7.64E-01 |
| **comp27916** | 6971.62 | **-0.53** | 0.32 | -1.67 | 9.40E-02 | 1.77E-01 | 6971.62 | **0.95** | 0.32 | 3.00 | 2.72E-03 | 2.54E-02 |
| **comp28208** | 539324.87 | **-2.45** | 0.73 | -3.36 | 7.93E-04 | 3.45E-03 | 539324.87 | **-0.68** | 0.73 | -0.93 | 3.54E-01 | 6.04E-01 |
| **comp28398** | 17199.85 | **-2.79** | 0.49 | -5.64 | 1.71E-08 | 2.54E-07 | 17199.85 | **-1.33** | 0.49 | -2.69 | 7.07E-03 | 5.03E-02 |
| **comp28548** | 2299.08 | **-2.05** | 0.37 | -5.58 | 2.46E-08 | 3.45E-07 | 2299.08 | **-1.76** | 0.37 | -4.78 | 1.75E-06 | 6.85E-05 |
| **comp28845** | 1637.86 | **-0.42** | 0.43 | -0.98 | 3.26E-01 | 4.59E-01 | 1637.86 | **0.03** | 0.43 | 0.08 | 9.36E-01 | 9.69E-01 |
| **comp28952** | 415310.81 | **-3.81** | 0.58 | -6.54 | 6.22E-11 | 1.52E-09 | 415310.81 | **-0.60** | 0.58 | -1.03 | 3.05E-01 | 5.57E-01 |
| **comp28956** | 6104.00 | **-0.90** | 0.35 | -2.54 | 1.11E-02 | 3.18E-02 | 6104.00 | **-0.04** | 0.35 | -0.12 | 9.08E-01 | 9.61E-01 |
| **comp28958** | 248312.40 | **-2.47** | 0.51 | -4.82 | 1.40E-06 | 1.25E-05 | 248312.40 | **-0.14** | 0.51 | -0.28 | 7.82E-01 | 9.07E-01 |
| **comp28960** | 262693.89 | **-2.18** | 0.43 | -5.09 | 3.62E-07 | 3.71E-06 | 262693.89 | **0.53** | 0.43 | 1.24 | 2.17E-01 | 4.54E-01 |
| **comp28979** | 141658.22 | **-2.43** | 0.44 | -5.54 | 3.02E-08 | 4.09E-07 | 141658.22 | **0.88** | 0.44 | 2.01 | 4.43E-02 | 1.70E-01 |
| **comp29022** | 26186.18 | **-2.51** | 0.38 | -6.57 | 5.09E-11 | 1.27E-09 | 26186.18 | **-0.57** | 0.38 | -1.50 | 1.34E-01 | 3.41E-01 |
| **comp29038** | 18806.74 | **-1.93** | 0.39 | -4.90 | 9.57E-07 | 8.83E-06 | 18806.74 | **-0.64** | 0.39 | -1.62 | 1.05E-01 | 2.93E-01 |
| **comp29110** | 10924.42 | **0.02** | 0.34 | 0.06 | 9.54E-01 | 9.70E-01 | 10924.42 | **-1.78** | 0.34 | -5.22 | 1.75E-07 | 9.53E-06 |
| **comp29132** | 21287.53 | **-2.72** | 0.45 | -6.04 | 1.59E-09 | 3.00E-08 | 21287.53 | **-2.09** | 0.45 | -4.63 | 3.73E-06 | 1.28E-04 |
| **comp29244** | 840.48 | **0.58** | 0.58 | 1.00 | 3.17E-01 | 4.49E-01 | 840.48 | **-0.92** | 0.58 | -1.58 | 1.13E-01 | 3.07E-01 |
| **comp29263** | 6175.49 | **-2.62** | 0.47 | -5.58 | 2.36E-08 | 3.34E-07 | 6175.49 | **-0.21** | 0.47 | -0.45 | 6.54E-01 | 8.35E-01 |
| **comp6510** | 132.61 | **1.29** | 0.57 | 2.29 | 2.22E-02 | 5.61E-02 | 132.61 | **0.08** | 0.58 | 0.14 | 8.89E-01 | 9.60E-01 |
| **comp7410** | 21.43 | **8.24** | 1.77 | 4.67 | 3.07E-06 | 2.51E-05 | 21.43 | **0.31** | 2.28 | 0.13 | 8.93E-01 | 9.60E-01 |
