# Supplementary material for: Tuberomics: a molecular profiling for the adaption of edible fungi (Tuber magnatum Pico) to different natural environments
Source: BMC Genomics. 2020 Jan 29;21:90. doi: 10.1186/s12864-020-6522-3 (PMC6988325; doi:10.1186/s12864-020-6522-3)
Supplement: Supplementary file 13 — Additional file 13: Table S9. T. magnatum genes involved in the sulfur metabolism and annotated in T. melanosporum. [file 12864_2020_6522_MOESM13_ESM.docx]

**Table S9: *T. magnatum* genes involved in the sulfur metabolism and annotated in *T. melanosporum*.** *T.magnatum* orthologous genes were selected after comparison with RNA-seq data. The table shows the list of primer pairs used in qPCR analysis of gene expression. Protein names correspond to the closest *T.melanosporum* homologous genes identified with sequence similarity search against the *T. magnatum* transcriptome published by Vita *et al.* [33]. 18S primer pairs were designed based on the deposited *T. magnatum* sequence. HK, housekeeping gene.

| **Code** | **Protein name** | ***T. melanosporum* Gene name** | ***T.magnatum* Gene name** | **Forward primer** | **Reverse Primer** |
| --- | --- | --- | --- | --- | --- |
| **1** | Sulfate permease | **GSTUMT00000861001** | **comp28958** | GAAGGGATCGGAAGGATTGG | CGCCCCGTCAATATCAACAT |
| **2** | ATP sulfurylase | **GSTUMT00002747001** | **comp24266** | GGCGTGGATGATCCCTATGA | CGCTCTCAAGGAGGAGGACA |
| **3** | Adenosine 5'-phosphosulfatekinase | **GSTUMT00003745001** | **comp16500** | TGACCGCCTCCTCAACAGAT | GGATTTCTGCCCCCTACGAG |
| **4** | PAPS reductase | **GSTUMT00002663001** | **comp28956** | TGCCAGTCCCCAATGTCTCT | GGTTCGGGAATACGTCACCA |
| **5** | Thioredoxin reductase | **GSTUMT00008708001** | **comp27916** | GGTTGGCCCTCCCTGACTAC | AAACGGGAGGAAAACGTGGT |
| **6** | 3'(2'),5'-bisphosphate nucleotidase | **GSTUMT00006994001** | **comp16628** | ACCCTGATGCTTCCCCTTGT | ACCAAGGACCTGACGAACGA |
| **7** | Sulfite reductase alpha subunit | **GSTUMT00000610001** | **comp22902** | CCTGGTAGGCTTCCCATTCC | ATTGGCTCCGTCCTGCTGTA |
| **8** | Homocysteine synthase | **GSTUMT00009485001** | **comp29038** | CGAGACCCTGGATCAACTGG | CTTTGGTCCGATCAGCTTCG |
| **9** | Cysteine synthase | **GSTUMT00003830001** | **comp28845** | ATAGGGGCTAGCGGCTTCAG | CCCCTCCTTCAGCTTCCTGT |
| **10** | Cystathionine beta-lyase | **GSTUMT00006346001** | **comp28548** | TTGTGCCCCCATAAAGATCG | AGCGCTTGTCGTTTCCTCTG |
| **11** | Cystathionine beta-synthase | **GSTUMT00006909001** | **comp29244** | CCCCTCAGACGAACCATCAC | AAGGCGAACGAGTGGGGTAT |
| **12** | Cystathionine gammalyase | **GSTUMT00010528001** | **comp28952** | GCAATGGGTGGTGGAATGAT | CCCCAAGAGACTCAGCGAGA |
| **13** | Cobalamin-independent Met synthase | **GSTUMT00005323001** | **comp28960** | CGCCGATGTTGAATCTCTCC | TCAAGGCAGCCAAGGAGTTC |
| **14** | Cysteine dioxygenase | **GSTUMT00004091001** | **comp22081** | ATCCCACCGACCTCATAGCA | GCTCCTGTCCTGGGGGATAC |
| **15** | Taurine dioxygenase | **GSTUMT00010867001** | **comp12411** | ACCTGATTTGCTGCGTCCAT | GCCGAATGGAAATGAACCAA |
| **16** | Peptide methionine sulfoxide reductase | **GSTUMT00004791001** | **comp22883** | CGCCGTCACTCCTCTCTGTT | CCGGTTTGTGAGGGGGTTAT |
| **17** | Branched-chain amino acid aminotransferase (mitochondrial) | **GSTUMT00000969001** | **comp29110** | CGCACCACCAGTACCAATCA | TTCCGCTTTGGAGGAATTGA |
| **18** | Aromatic amino acid amino transferase | **GSTUMT00009292001** | **comp24488** | CGGTTGGTTAGAGGGGCTTC | CTCTCGAGGACAACGGTGCT |
| **19** | 2-Oxo acid/ phenylpyruvate decarboxylase | **GSTUMT00006321001** | **comp28979** | CTCCCCACCGACATTGTCTC | GGGATCATTGTCGGGGATTT |
| **HK^a^** | *Tuber magnatum* 18S |  | **AF054901.1** | ACTAGGGATCGGGCGATGTT | CAGCCTTGCGACCATACTCC |
