# Supplementary material for: Tuberomics: a molecular profiling for the adaption of edible fungi (Tuber magnatum Pico) to different natural environments
Source: BMC Genomics. 2020 Jan 29;21:90. doi: 10.1186/s12864-020-6522-3 (PMC6988325; doi:10.1186/s12864-020-6522-3)
Supplement: Supplementary file 15 — Additional file 15: Table S10. Compounds identified through GC-MS analysis. [file 12864_2020_6522_MOESM15_ESM.docx]

**Table S10: Compounds identified through GC-MS analysis.** ^a^ = Progressive code associated to MS analyses. LRI = Linear Retention Index.

| **Progressive code^a^** | **Compounds** | **Experimental LRI** | **Reference**  **LRI** | **Chemical formula** | **Molecular weight** |
| --- | --- | --- | --- | --- | --- |
|  | **Sulfur-containing volatiles** |  |  |  |  |
| **3** | *methanethiol* | **307** |  | **CH_4_S** | **48,11** |
| **9** | *dimethyl sulfide* | **314** | **296** | **C_2_H_6_S** | **62,13** |
| **34** | *s-methyl thioacetate* | **448** | **449** | **C_3_H_6_OS** | **90,14** |
| **37** | *sulfonylbis-methane* | **1295** |  | **C_2_H_6_O_2_S** | **94,13** |
| **38** | *dimethyl disulfide* | **469** | **465** | **C_2_H_6_S_2_** | **94,20** |
| **59** | *2-oxo-2,4-dithiapentane* | **1476** |  | **C_3_H_8_S_2_** | **108,22** |
| **60** | *bis-(methylthio)-methane* | **687** | **692** | **C_3_H_8_S_2_** | **108,23** |
| **71** | *1-methylthio-pentane* | **508** |  | **C_6_H_14_S** | **118,24** |
| **88** | *dimethyl-trisulfide* | **775** | **785** | **C_2_H_6_S_3_** | **126,26** |
| **120** | *methyl (methylthio)methyl-disulfide* | **1657** |  | **C_3_H_8_S_3_** | **140,29** |
| **136** | *tris(methylthio)-methane* | **1236** |  | **C_4_H_10_S_3_** | **154,30** |
|  | **Alcohols** |  |  |  |  |
| **2** | *ethanol* | **369** |  | **C_2_H_6_O** | **46,07** |
| **7** | *1-propanol* | **441** | **434** | **C_3_H_8_O** | **60,10** |
| **8** | *2-propanol* | **366** |  | **C_3_H_8_O** | **60,10** |
| **17** | *2-butanol* | **429** | **428** | **C_4_H_10_O** | **74,12** |
| **18** | *2-methyl-1-propanol* | **494** | **480** | **C_4_H_10_O** | **74,12** |
| **29** | *3-methyl-1-butanol* | **626** | **618** | **C_5_H_12_O** | **88,15** |
| **30** | *2-methylbutanol* | **617** | **611** | **C_5_H_12_O** | **88,15** |
| **32** | *sec-pentanol* | **514** | **514** | **C_5_H_12_O** | **88,15** |
| **33** | *3-methyl-2-butanol* | **498** |  | **C_5_H_12_O** | **88,15** |
| **36** | *phenyl alcohol* | **1387** | **1394** | **C_6_H_6_O** | **94,11** |
| **51** | *n-hexanol* | **764** | **758** | **C_6_H_14_O** | **102,18** |
| **52** | *2,3-dimethyl-1-butanol* | **701** |  | **C_6_H_14_O** | **102,18** |
| **70** | *n-heptanol* | **863** | **854** | **C_7_H_16_O** | **116,20** |
| **92** | *3z-octenol* | **980** | **983** | **C_8_H_16_O** | **128,21** |
| **94** | *1-octen-3-ol* | **858** | **858** | **C_8_H_16_O** | **128,22** |
| **100** | *2-ethylhexanol* | **897** |  | **C_8_H_18_O** | **130,23** |
| **106** | *2-phenyl-2-propanol* | **1154** | **1151** | **C_9_H_12_O** | **136,19** |
| **128** | *2-nonanol* | **924** | **925** | **C_9_H_20_O** | **144,26** |
| **129** | *nonylol* | **913** | **915** | **C_9_H_20_O** | **144,26** |
| **147** | *undec-3-en-2-ol* | **1137** |  | **C_11_H_22_O** | **170,30** |
|  | **Aldehydes** |  |  |  |  |
| **1** | *acetaldehyde* | **309** | **294** | **C_2_H_4_O** | **44,05** |
| **5** | *propionaldehyde* | **322** | **314** | **C_3_H_6_O** | **58,08** |
| **13** | *2-butenal* | **449** | **440** | **C_4_H_6_O** | **70,09** |
| **15** | *2-methyl-propanal* | **324** | **331** | **C_4_H_8_O** | **72,11** |
| **16** | *butanal* | **354** |  | **C_4_H_8_O** | **72,11** |
| **19** | *2-methyl- 2-butenal* | **496** | **489** | **C_5_H_8_O** | **84,12** |
| **23** | *3-methylbutanal* | **362** | **368** | **C_5_H_10_O** | **86,13** |
| **25** | *2-methylbutanal* | **360** | **366** | **C_5_H_10_O** | **86,13** |
| **45** | *hexanal* | **479** | **477** | **C_6_H_12_O** | **100,16** |
| **46** | *2-methyl-pentanal* | **405** |  | **C_6_H_12_O** | **100,16** |
| **61** | *2-(e)-heptenal* | **733** | **728** | **C_7_H_12_O** | **112,17** |
| **66** | *heptanal* | **597** | **598** | **C_7_H_14_O** | **114,19** |
| **83** | *2-(e)-octenal* | **840** | **842** | **C_8_H_14_O** | **126,20** |
| **93** | *octanal* | **694** | **698** | **C_8_H_16_O** | **128,21** |
| **101** | *3-phenyl-2-propenal* | **1225** | **1218** | **C_9_H_8_O** | **132,16** |
| **102** | *isophthalaldehyde* | **1516** |  | **C_8_H_6_O_2_** | **134,13** |
| **104** | *p-ethylbenzaldehyde* | **1134** | **1139** | **C_9_H_10_O** | **134,18** |
| **116** | *ligustral* | **889** | **890** | **C_9_H_14_O** | **138,21** |
| **117** | *2,4-(e,e)-nonadienal* | **1109** |  | **C_9_H_14_O** | **138,21** |
| **118** | *2-(e)-nonenal* | **943** |  | **C_9_H_16_O** | **140,23** |
| **122** | *nonanal* | **801** | **800** | **C_9_H_18_O** | **142,24** |
| **130** | *2-phenyl-crotonaldehyde* | **1325** | **1320** | **C_10_H_10_O** | **146,19** |
| **133** | *(4z)-decenal* | **941** | **940** | **C_10_H_18_O** | **154,25** |
| **137** | *decanal* | **906** | **910** | **C_10_H_20_O** | **156,20** |
| **156** | *dodecanal* | **1112** |  | **C_12_H_24_O** | **184,32** |
|  | **Aromatic compounds** |  |  |  |  |
| **35** | *toluene* | **438** | **430** | **C_7_H_8_** | **92,14** |
| **53** | *ethenylbenzene* | **658** |  | **C_8_H_8_** | **104,15** |
| **54** | *benzaldehyde* | **925** | **925** | **C_7_H_6_O** | **106,12** |
| **55** | *1,4-dimethyl-benzene* | **523** |  | **C_8_H_10_** | **106,16** |
| **56** | *1,3-dimethyl-benzene* | **528** |  | **C_8_H_10_** | **106,16** |
| **57** | *ethylbenzene* | **515** | **525** | **C_8_H_10_** | **106,17** |
| **58** | *1,2-dimethyl-benzene* | **579** |  | **C_8_H_10_** | **106,17** |
| **72** | *phenylacetaldehyde* | **1040** | **1044** | **C_8_H_8_O** | **120,15** |
| **74** | *1,3,5-trimethyl-benzene* | **643** |  | **C_9_H_12_** | **120,19** |
| **75** | *cumene* | **562** | **584** | **C_9_H_12_** | **120,20** |
| **76** | *benzamide* | **2056** |  | **C_7_H_7_NO** | **121,14** |
| **77** | *phenethyl alcohol* | **1297** | **1306** | **C_8_H_10_O** | **122,16** |
| **78** | *1-phenylethanol* | **1207** | **1208** | **C_8_H_10_O** | **122,17** |
|  | **Esters** |  |  |  |  |
| **27** | *ethylacetate* | **349** |  | **C_4_H_8_O_2_** | **88,11** |
| **49** | *acetic acid, propyl ester* | **402** |  | **C_5_H_10_O_2_** | **102,13** |
| **50** | *methyl isobutyrate* | **361** | **364** | **C_5_H_10_O_2_** | **102,13** |
| **68** | *3-methylbutanoic acid methyl ester* | **426** | **422** | **C_6_H_12_O_2_** | **116,16** |
| **96** | *ethyl-acetoacetate* | **865** | **863** | **C_6_H_10_O_3_** | **130,14** |
| **97** | *2-methyl-ethyl-butyrate* | **452** | **452** | **C_7_H_14_O_2_** | **130,19** |
| **98** | *2-methylbutyl-acetate* | **516** | **524** | **C_7_H_14_O_2_** | **130,19** |
| **99** | *ethyl isovalerate* | **467** | **465** | **C_7_H_14_O_2_** | **130,19** |
| **127** | *ethyl-hexanoate* | **640** | **637** | **C_8_H_16_O_2_** | **144,21** |
| **142** | *tetrahydrofurfuryl propionate* | **1045** | **1044** | **C_8_H_14_O_3_** | **158,20** |
| **149** | *3-methyl pentyl-isobutyrate* | **710** | **709** | **C_10_H_20_O_2_** | **172,27** |
| **150** | *hexyl isobutyrate* | **750** | **748** | **C_10_H_20_O_2_** | **172,27** |
| **153** | *allyl caprylate* | **977** | **979** | **C_11_H_20_O_2_** | **184,28** |
| **155** | *(6z)-6-nonenyl acetate* | **1031** | **1033** | **C_11_H_20_O_2_** | **184,28** |
| **159** | *heptyl-isobutyrate* | **855** | **857** | **C_11_H_22_O_2_** | **186,29** |
| **160** | *ethyl-nonanoate* | **945** | **948** | **C_11_H_22_O_2_** | **186,30** |
| **161** | *trans-geranylacetone* | **1249** | **1250** | **C_13_H_22_O** | **194,31** |
| **163** | *(e)-2-methyl-2-butenoic acid 1-ethylhexyl ester* | **1056** | **1057** | **C_13_H_24_O_2_** | **212,33** |
| **164** | *dodecanoic acid, methyl ester* | **1199** | **1200** | **C_13_H_26_O_2_** | **214,34** |
| **165** | *α-,α-dimethyl-phenethyl butyrate* | **1301** | **1303** | **C_14_H_20_O_2_** | **220,31** |
|  | **Hydrocarbons** |  |  |  |  |
| **20** | *cyclohexane* | **309** | **301** | **C_6_H_12_** | **84,16** |
| **42** | *2-heptene* | **459** | **456** | **C_7_H_14_** | **98,19** |
| **80** | *3-ethyl-2-methyl-1,3-hexadiene* | **820** |  | **C_9_H_16_** | **124,23** |
| **95** | *n-nonane* | **357** | **364** | **C_9_H_20_** | **128,26** |
| **119** | *1-decene* | **446** | **444** | **C_10_H_20_** | **140,27** |
| **126** | *decane* | **412** | **406** | **C_10_H_22_** | **142,28** |
| **135** | *1-undecene* | **540** |  | **C_11_H_22_** | **154,30** |
| **141** | *undecane* | **488** | **492** | **C_11_H_24_** | **156,31** |
| **144** | *1,3-diisopropylbenzene* | **1719** |  | **C_12_H_18_** | **162,28** |
| **145** | *1-dodecene* | **651** | **657** | **C_12_H_24_** | **168,32** |
| **148** | *dodecane* | **600** | **604** | **C_12_H_26_** | **170,34** |
| **152** | *1-tridecene* | **756** | **759** | **C_13_H_26_** | **182,35** |
| **158** | *tridecane* | **706** | **700** | **C_13_H_28_** | **184,37** |
|  | **Ketones** |  |  |  |  |
| **4** | *acetone* | **328** | **341** | **C_3_H_6_O** | **58,08** |
| **14** | *2-butanone* | **354** | **350** | **C_4_H_8_O** | **72,11** |
| **21** | *γ-butyrolactone* | **1025** | **1018** | **C_4_H_6_O_2_** | **86,09** |
| **22** | *2-pentanone* | **398** | **397** | **C_5_H_10_O** | **86,13** |
| **24** | *3-methyl-2-butanone* | **369** |  | **C_5_H_10_O** | **86,13** |
| **26** | *3-pentanone* | **397** | **388** | **C_5_H_10_O** | **86,13** |
| **28** | *acetoin* | **702** |  | **C_4_H_8_O_2_** | **88,11** |
| **41** | *4-methyl-3-penten-2-one* | **533** | **530** | **C_6_H_10_O** | **98,14** |
| **43** | *2,4-(3h,5h)-furandione* | **677** |  | **C_4_H_4_O_3_** | **100,07** |
| **44** | *2,3-pentanedione* | **461** | **456** | **C_5_H_8_O_2_** | **100,12** |
| **47** | *2-methyl-3-pentanone* | **460** |  | **C_6_H_12_O** | **100,16** |
| **48** | *4-methyl-2-pentanone* | **417** | **410** | **C_6_H_12_O** | **100,16** |
| **62** | *2(3h)-dihydro-3,5-dimethyl-furanone* | **969** |  | **C_6_H_10_O_2_** | **114,14** |
| **63** | *2,5-hexanedione* | **647** |  | **C_6_H_10_O_2_** | **114,14** |
| **64** | *2,3-hexanedione* | **531** | **521** | **C_6_H_10_O_2_** | **114,14** |
| **65** | *2-heptanone* | **592** | **597** | **C_7_H_14_O** | **114,19** |
| **67** | *4-heptanone* | **521** | **527** | **C_7_H_14_O** | **114,19** |
| **69** | *4-hydroxy-3-hexanone* | **817** |  | **C_6_H_12_O_2_** | **116,16** |
| **73** | *1-phenyl-ethanone* | **1054** | **1054** | **C8H8O** | **120,15** |
| **79** | *2-acetyl-5-methylfuran* | **1018** | **1020** | **C_7_H_8_O_2_** | **124,14** |
| **81** | *2-acetylcyclopentanone* | **1002** | **1012** | **C_7_H_10_O_2_** | **126,15** |
| **82** | *3,4-dimethyl-cyclopentan-1,2-dione* | **1046** | **1050** | **C_7_H_10_O_2_** | **126,16** |
| **84** | *6-methyl-5-hepten-2-one* | **744** | **746** | **C_8_H_14_O** | **126,20** |
| **85** | *3-octen-2-one* | **816** | **816** | **C_8_H_14_O** | **126,20** |
| **86** | 3,4-dimethyl-3-hexen-2-one | **984** |  | **C_8_H_14_O** | **126,20** |
| **87** | *1-octen-3-one* | **709** |  | **C_8_H_14_O** | **126,20** |
| **89** | *5-methyl-2,3-hexanedione* | **555** | **552** | **C_7_H_12_O_2_** | **128,17** |
| **90** | *3-octanone* | **662** | **658** | **C_8_H_16_O** | **128,21** |
| **91** | *2-octanone* | **695** |  | **C_8_H_16_O** | **128,21** |
| **103** | *1-3-methylphenyl-ethanone* | **1149** |  | **C_9_H_10_O** | **134,18** |
| **121** | *2-nonanone* | **797** | **802** | **C_9_H_18_O** | **142,24** |
| **123** | *2,2-dimethyl-3-heptanone* | **837** |  | **C_9_H_18_O** | **142,24** |
| **124** | *diisobutyl ketone* | **584** | **587** | **C_9_H_18_O** | **142,24** |
| **125** | *4-nonanone* | **734** | **734** | **C_9_H_18_O** | **142,24** |
| **131** | *1-(3,4-dimethylphenyl)-ethanone* | **1627** |  | **C_10_H_12_O** | **148,20** |
| **132** | *4'-ethyl-acetophenone* | **1221** | **1227** | **C_10_H_12_O** | **148,21** |
| **134** | *3-undecen-2-one* | **1125** |  | **C_10_H_18_O** | **154,25** |
| **138** | *2(3h)-diihydro-5-pentyl-furanone* | **1414** | **1419** | **C_9_H_16_O_2_** | **156,22** |
| **139** | *methyl octyl ketone* | **905** | **906** | **C_10_H_20_O** | **156,27** |
| **143** | *1,4-diacetyl-benzene* | **1747** | **1747** | **C_10_H_10_O_2_** | **162,19** |
| **146** | *2-undecanone* | **1003** |  | **C_11_H_22_O** | **170,29** |
| **157** | *2-dodecanone* | **1074** | **1072** | **C_12_H_24_O** | **184,32** |
| **162** | *2-tridecanone* | **1208** | **1213** | **C_13_H_26_O** | **198,35** |
|  | **Terpenes** |  |  |  |  |
| **105** | *para-cymene* | **677** | **678** | **C_10_H_14_** | **134,22** |
| **107** | *limonene* | **601** | **608** | **C_10_H_16_** | **136,23** |
| **108** | *γ-terpinene* | **652** | **654** | **C_10_H_16_** | **136,23** |
| **109** | *2,2-dimethyl-5-methylene norbornane* | **460** | **464** | **C_10_H_16_** | **136,23** |
| **110** | *β-pinene* | **490** | **505** | **C_10_H_16_** | **136,23** |
| **111** | *α-pinene* | **424** | **427** | **C_10_H_16_** | **136,23** |
| **112** | *sabinene* | **516** | **518** | **C_10_H_16_** | **136,23** |
| **113** | *α-fenchene* | **452** | **454** | **C_10_H_16_** | **136,24** |
| **114** | *myrcene* | **568** | **567** | **C_10_H_16_** | **136,24** |
| **140** | *α-dihydro-terpineol* | **996** | **992** | **C_10_H_20_O** | **156,27** |
|  | **Others** |  |  |  |  |
| **6** | *n,n-dimethyl-methanamine* | **300** |  | **C_3_H_9_N** | **59,11** |
| **10** | *pyrrole* | **916** | **920** | **C_4_H_5_N** | **67,09** |
| **11** | *n-vinylaziridine* | **455** |  | **C_4_H_7_N** | **69,11** |
| **12** | *δ-1-pyrroline* | **417** |  | **C_4_H_7_N** | **69,11** |
| **31** | *tert-butyl methyl ether* | **290** | **284** | **C_5_H_12_O** | **88,15** |
| **39** | *2,5-dimethyl-furan* | **382** | **384** | **C_6_H_8_O** | **96,13** |
| **40** | *2,4-dimethylfuran* | **389** |  | **C_6_H_8_O** | **96,13** |
| **115** | *2-pentylfuran* | **632** | **638** | **C_9_H_14_O** | **138,21** |
| **151** | *diethyl acetal* | **361** | **360** | **C_10_H_24_O_2_** | **176,30** |
| **154** | *(z)-3-nonenyl acetate* | **1008** | **1010** | **C_11_H_20_O_2_** | **184,28** |
