# Supplementary material for: Tuberomics: a molecular profiling for the adaption of edible fungi (Tuber magnatum Pico) to different natural environments
Source: BMC Genomics. 2020 Jan 29;21:90. doi: 10.1186/s12864-020-6522-3 (PMC6988325; doi:10.1186/s12864-020-6522-3)
Supplement: Supplementary file 17 — Additional file 17: Table S12. VIPs compounds (GC-MS data) according to PLS-DA analysis. Compounds that displayed VIP scores ≥2 (bold marked) in at least in one sample are shown. [file 12864_2020_6522_MOESM17_ESM.docx]

**Table S12: VIPs compounds (GC-MS data) according to PLS-DA analysis.** Compounds that displayed VIP scores ≥ 2 (bold marked) in at least in one sample are shown. VOCs grouped based on their chemical class. The best VIP values are marked in yellow, for each selected compound. ^a^ Progressive code associated to each compound, as reported in Additional file 15: Table S10.

| **Compound list^a^** | **VIP Scores**  **for AL** | **VIP Scores**  **for IS** | **VIP Scores**  **for SM** | **Compound name** | **Chemical class** |
| --- | --- | --- | --- | --- | --- |
| ***2*** | 1.59 | **3.17** | 0.76 | ***ethanol*** | **Alcohols** |
| ***7*** | **2.92** | **3.88** | **2.42** | ***1-propanol*** | **Alcohols** |
| ***51*** | 1.57 | **2.84** | 0.90 | ***n-hexanol*** | **Alcohols** |
| ***94*** | **2.25** | 0.65 | **3.08** | ***1-octen-3-ol*** | **Alcohols** |
| **13** | **2.03** | 1.85 | **2.13** | ***2-butenal*** | **Aldehydes** |
| ***25*** | 1.29 | **2.89** | 0.45 | ***2-methylbutanal*** | **Aldehydes** |
| ***45*** | 1.69 | 0.78 | **2.17** | ***hexanal*** | **Aldehydes** |
| ***83*** | 1.51 | **2.08** | 1.21 | ***2-(e)-octenal*** | **Aldehydes** |
| **122** | 1.99 | 0.41 | **2.81** | ***nonanal*** | **Aldehydes** |
| ***133*** | 1.47 | **3.47** | 0.43 | ***(4z)-decenal*** | **Aldehydes** |
| ***156*** | 1.94 | **4.58** | 0.56 | ***dodecanal*** | **Aldehydes** |
| ***35*** | **2.76** | 0.74 | **3.81** | ***toluene*** | **Aromatic compounds** |
| ***54*** | **2.30** | **4.79** | 1.01 | ***benzaldehyde*** | **Aromatic compounds** |
| ***55*** | **2.50** | 0.66 | **3.46** | ***1,4-dimethyl-benzene*** | **Aromatic compounds** |
| ***56*** | 1.71 | 0.45 | **2.37** | ***1,3-dimethyl-benzene*** | **Aromatic compounds** |
| ***57*** | **2.25** | 0.39 | **3.22** | ***ethylbenzene*** | **Aromatic compounds** |
| ***58*** | **2.51** | 0.66 | **3.47** | ***1,2-dimethyl-benzene*** | **Aromatic compounds** |
| ***75*** | **2.07** | 0.07 | **3.12** | ***cumene*** | **Aromatic compounds** |
| ***77*** | 1.73 | **3.06** | 1.03 | ***phenethyl alcohol*** | **Aromatic compounds** |
| **78** | **2.04** | 1.86 | **2.14** | ***1-phenylethanol*** | **Aromatic compounds** |
| ***96*** | 1.72 | **4.05** | 0.50 | ***ethyl-acetoacetate*** | **Esters** |
| ***152*** | 1.72 | **4.05** | 0.50 | ***1-tridecene*** | **Hydrocarbons** |
| ***41*** | 1.00 | **2.35** | 0.29 | ***4-methyl-3-penten-2-one*** | **Ketones** |
| ***63*** | 1.20 | **2.53** | 0.51 | ***2,5-hexanedione*** | **Ketones** |
| ***65*** | 1.59 | **2.88** | 0.91 | ***2-heptanone*** | **Ketones** |
| ***67*** | 1.95 | **4.60** | 0.56 | ***4-heptanone*** | **Ketones** |
| ***73*** | 1.54 | **3.47** | 0.53 | ***1-phenyl-ethanone*** | **Ketones** |
| **79** | 1.79 | **2.01** | 1.67 | ***2-acetyl-5-methylfuran*** | **Ketones** |
| **81** | **2.01** | 0.17 | **2.98** | ***2-acetylcyclopentanone*** | **Ketones** |
| ***84*** | **2.19** | 0.48 | **3.08** | ***6-methyl-5-hepten-2-one*** | **Ketones** |
| **85** | 1.47 | 0.40 | **2.03** | ***3-octen-2-one*** | **Ketones** |
| **90** | 1.72 | **2.91** | 1.09 | ***3-octanone*** | **Ketones** |
| **131** | **2.04** | 1.86 | **2.14** | ***1-(3,4-dimethylphenyl)-ethanone*** | **Ketones** |
| ***139*** | 1.47 | 0.40 | **2.02** | ***methyl octyl ketone*** | **Ketones** |
| ***161*** | **2.15** | 0.53 | **2.99** | ***trans-geranylacetone*** | **Ketones** |
| ***31*** | 1.72 | **4.05** | 0.50 | ***tert-butyl methyl ether*** | **Others** |
| ***39*** | 1.72 | **4.05** | 0.50 | ***2,5-dimethyl-furan*** | **Others** |
| ***115*** | **3.78** | 1.02 | **5.22** | ***2-pentylfuran*** | **Others** |
| ***37*** | 1.47 | **3.05** | 0.64 | ***sulfonylbis-methane*** | **Sulfur-containing volatiles** |
| ***88*** | **4.59** | **4.23** | **4.77** | ***dimethyl-trisulfide*** | **Sulfur-containing volatiles** |
| ***136*** | 1.47 | **3.20** | 0.56 | ***tris(methylthio)-methane*** | **Sulfur-containing volatiles** |
| ***112*** | 1.47 | **3.47** | 0.43 | ***sabinene*** | **Terpenes** |
