# Supplementary material for: Tuberomics: a molecular profiling for the adaption of edible fungi (Tuber magnatum Pico) to different natural environments
Source: BMC Genomics. 2020 Jan 29;21:90. doi: 10.1186/s12864-020-6522-3 (PMC6988325; doi:10.1186/s12864-020-6522-3)
Supplement: Supplementary file 18 — Additional file 18: Table S13. Compounds identified through PTR-ToF analysis conducted during 4 years of experimental work. [file 12864_2020_6522_MOESM18_ESM.docx]

**Table S13: Compounds identified through PTR-ToF analysis conducted during 4 years of experimental work.** ^a^ Progressive code associated to MS analyses. Meas. = measured; Theor. = theoretical. For more details see the main text (Methods section).

| **Progressive code^a^** | **Putative Identifications** | **Chemical**  **Formula** | **Meas.**  ***m/z*^b^** | **Theor.**  ***m/z*^c^** |
| --- | --- | --- | --- | --- |
|  | **Sulfur-containing volatiles** |  |  |  |
| **8** | methanethial | **CH_2_S** | **46.990** | **46.995** |
| **10** | methanethiol | **CH_4_S** | **49.011** | **49.011** |
| **15** | Sulfur compounds (acetylenethiol / thiirene) | **C_2_H_2_S** | **58.993** | **58.995** |
| **18** | dimethyl sulfide | **C_2_H_6_S** | **63.026** | **63.026** |
| **29** | 1-propanethiol | **C_3_H_8_S** | **77.010** | **77.006** |
| **30** | Sulfur compounds (dithioformic acid) | **CH_2_S_2_** | **78.984** | **78.96** |
| **31** | dimethyl sulfoxide | **C_2_H_6_OS** | **79.021** | **79.021** |
| **38** | allyl methyl sulfide | **C_4_H_8_S** | **89.041** | **89.042** |
| **41** | 1,2-dithietane | **C_2_H_4_S_2_** | **92.980** | **92.983** |
| **42** | 2-methylmercaptoethanol | **C_3_H_8_OS** | **93.035** | **93.037** |
| **44** | dimethyl sulfone | **C_2_H_6_O_2_S** | **95.010** | **95.004** |
| **45** | dimethyl disulfide | **C_2_H_6_S_2_** | **95.199** | **94.998** |
| **51** | 2-methyl-4,5-dihydrothiophene | **C_5_H_8_S** | **101.040** | **101.042** |
| **53** | 3-methylthio-propionaldehyde | **C_4_H_8_OS** | **105.037** | **105.037** |
| **55** | 2-methylthioacetic acid | **C_3_H_6_O_2_S** | **106.995** | **107.016** |
| **57** | 2-ethynylthiophene | **C_6_H_4_S** | **109.010** | **109.011** |
| **60** | 2-methyl-3-furanthiol | **C_5_H_6_OS** | **115.020** | **115.021** |
|  | **Alcohols** |  |  |  |
| **3** | methanol | **CH_4_O** | **33.033** | **33.034** |
| **9** | ethanol | **C_2_H_6_O** | **47.049** | **47.049** |
| **40** | 2-3-butanediol | **C_4_H_10_O_2_** | **91.057** | **91.075** |
| **62** | *n*-heptanol | **C_7_H_16_O** | **117.078** | **117.073** |
|  | **Aldehydes** |  |  |  |
| **2** | formaldehyde | **CH_2_O** | **31.042** | **31.018** |
| **7** | acetic aldehyde | **C_2_H_4_O** | **45.033** | **45.033** |
| **13** | 2-propenal | **C_3_H_4_O** | **57.034** | **57.033** |
| **24** | (2E)-butenal | **C_4_H_6_O** | **71.049** | **71.049** |
| **46** | furfural (2-furancarboxaldehyde) | **C_5_H_4_O_2_** | **97.025** | **97.028** |
|  | **Aromatic compounds** |  |  |  |
| **22** | furan | **C_4_H_4_O** | **69.033** | **69.034** |
| **33** | 2-methylfuran | **C_5_H_6_O** | **83.049** | **83.049** |
| **43** | toluene | **C_7_H_8_** | **93.068** | **93.070** |
| **52** | ethynylbenzene | **C_8_H_6_** | **103.050** | **103.054** |
| **56** | ethylbenzene | **C_8_H_10_** | **107.086** | **107.086** |
| **58** | anisole | **C_7_H_8_O** | **109.065** | **109.065** |
|  | **Esters** |  |  |  |
| **28** | methyl acetate | **C_3_H_6_O_2_** | **75.044** | **75.044** |
| **36** | vinyl acetate | **C_4_H_6_O_2_** | **87.043** | **87.044** |
| **64** | methyl 2-Furancarboxylate | **C_6_H_6_O_3_** | **127.035** | **127.039** |
|  | **Hydrocarbons** |  |  |  |
| **1** | acetylene | **C_2_H_2_** | **27.025** | **27.023** |
| **4** | alkyl fragment | **C_3_H_4_** | **41.039** | **41.039** |
| **6** | alkyl fragment (propene) | **C_3_H_6_** | **43.054** | **43.054** |
| **11** | C4 compound | **C_4_H_4_** | **53.038** | **53.039** |
| **12** | alkyl fragment | **C_4_H_6_** | **55.054** | **55.054** |
| **14** | alkyl fragment (1-Butene) | **C_4_H_8_** | **57.068** | **57.070** |
| **20** | 3penten-1-yne | **C_5_H_6_** | **67.054** | **67.054** |
| **25** | alkyl fragment | **C_5_H_10_** | **71.086** | **71.086** |
| **32** | alkyl fragment (hexenals/hexenols/terpenoids) | **C_6_H_8_** | **81.069** | **81.070** |
| **34** | C6 compounds | **C_6_H_10_** | **83.085** | **83.086** |
|  | **Ketones** |  |  |  |
| **5** | alkyl fragment (ethenone) | **C_2_H_2_O** | **43.018** | **43.018** |
| **16** | acetone | **C_3_H_6_O** | **59.049** | **59.049** |
| **27** | butanone | **C_4_H_8_O** | **73.065** | **73.065** |
| **35** | 3-penten-2-one | **C_5_H_8_O** | **85.064** | **85.065** |
| **37** | 3-pentanone | **C_5_H_10_O** | **87.081** | **87.080** |
| **39** | 3-hydroxy-2-butanone | **C_4_H_8_O_2_** | **89.056** | **89.060** |
| **48** | 4-methyl-(5H)-furan-2-one | **C_5_H_6_O_2_** | **99.044** | **99.044j** |
| **49** | 4-methyl-3-pentene-2-one | **C_6_H_10_O** | **99.080** | **99.080** |
| **50** | acetylpropionyl | **C_5_H_8_O_2_** | **101.060** | **101.060** |
| **61** | 3,5-dimethyldiidro-2(3H)-furanone | **C_6_H_10_O_2_** | **115.075** | **115.075** |
| **63** | 2-acetyl-5-methylfuran | **C_7_H_8_O_2_** | **125.010** | **125.009** |
| **65** | 3-octen-2-one | **C_8_H_14_O** | **127.112** | **127.112** |
|  | **Terpenes** |  |  |  |
| **23** | 2-methyl-1,3- butadiene | **C_5_H_8_** | **69.070** | **69.070** |
|  | **Others** |  |  |  |
| **17** | acetic acid | **C_2_H_4_O_2_** | **61.028** | **61.028** |
| **19** | methanetriol | **CH_4_O_3_** | **65.023** | **65.023** |
| **21** | pyrrole | **C_4_H_5_N** | **68.050** | **68.049** |
| **26** | acrylic Acid | **C_3_H_4_O_2_** | **73.030** | **73.028** |
| **47** | 2,5-dimethyl-furan | **C_6_H_8_O** | **97.065** | **97.065** |
| **54** | 4-hydroxybutanoic acid | **C_4_H_8_O_3_** | **105.060** | **105.055** |
| **59** | 2-acetylfuran | **C_6_H_6_O_2_** | **111.044** | **111.043** |
