# Supplementary material for: Tuberomics: a molecular profiling for the adaption of edible fungi (Tuber magnatum Pico) to different natural environments
Source: BMC Genomics. 2020 Jan 29;21:90. doi: 10.1186/s12864-020-6522-3 (PMC6988325; doi:10.1186/s12864-020-6522-3)
Supplement: Supplementary file 19 — Additional file 19: Table S14. PLS-DA (PTR-ToF-MS model) statistics for each Y-Block (class 1 = Alba; class 2 = Isernia; class 3 = San Miniato) related to 92 truffle samples. [file 12864_2020_6522_MOESM19_ESM.docx]

**Table S14:** **PLS-DA (PTR-ToF-MS model) statistics for each Y-Block (class 1 = Alba; class 2 = Isernia; class 3 = San Miniato) related to 92 truffle samples.** (**a**) Sensitivity (SE), Specificity (SP), Class. error and root mean standard deviation (RMSE) are reported for for Calibration (Cal or C), Cross Validation (CV), and Prediction (Pred or P), respectively. LV, latent variables. (**b**) Confusion matrices for Calibration, Cross Validation, and Prediction. (**c**) Probability of PLS-DA model insignificance vs. permuted samples for a model with two components. Values less than 0.05 indicate the model is significant at the 95% confidence level.

**a**

| **Statistics** | **LVs** | **SE (Cal)** | **SP (Cal)** | **SE (CV)** | **SP (CV)** | **SE (P)** | **SP (P)** | **Class. error (Cal)** | **Class. error (CV)** | **Class. error (Pred)** | **RMSEC** | **RMSECV** | **RMSEP** |
| --- | --- | --- | --- | --- | --- | --- | --- | --- | --- | --- | --- | --- | --- |
| **Alba** | 2 | 0.905 | 1.000 | 0.905 | 1.000 | 0.833 | 1.000 | 0.048 | 0.048 | 0.083 | 0.241 | 0.257 | 0.248 |
| **Isernia** | 2 | 1.000 | 1.000 | 1.000 | 1.000 | 1.000 | 1.000 | 0.000 | 0.000 | 0.000 | 0.176 | 0.188 | 0.184 |
| **San Miniato** | 2 | 1.000 | 0.976 | 1.000 | 0.951 | 1.000 | 1.000 | 0.012 | 0.024 | 0.000 | 0.295 | 0.307 | 0.305 |

**b**

|  | | ***Classes*** | **1-Alba** | **2-Isernia** | **3- San Miniato** |
| --- | --- | --- | --- | --- | --- |
| **Calibration results** | Predicted as | **1- Alba** | 19 | 0 | 0 |
|  |  | **2- Isernia** | 0 | 20 | 0 |
|  |  | **3- San Miniato** | 2 | 0 | 35 |
| **Cross validation results** |  | **1- Alba** | 19 | 0 | 0 |
|  |  | **2- Isernia** | 0 | 20 | 0 |
|  |  | **3- San Miniato** | 2 | 0 | 35 |
| **Prediction results** |  | **1- Alba** | 5 | 0 | 0 |
|  |  | **2- Isernia** | 0 | 4 | 0 |
|  |  | **3- San Miniato** | 1 | 0 | 6 |

**c**

|  | ***Wilcoxon*** | ***Sign Test*** | ***Rand t-test*** |
| --- | --- | --- | --- |
| **Alba** |  | | |
| Self-Prediction | 0.000 | 0.008 | 0.005 |
| Cross-Validated | 0.000 | 0.002 | 0.005 |
| **Isernia** |  | | |
| Self-Prediction | 0.000 | 0.000 | 0.005 |
| Cross-Validated | 0.000 | 0.000 | 0.005 |
| **San Miniato** |  | | |
| Self-Prediction | 0.000 | 0.001 | 0.005 |
| Cross-Validated | 0.000 | 0.000 | 0.005 |
