# Supplementary material for: Tuberomics: a molecular profiling for the adaption of edible fungi (Tuber magnatum Pico) to different natural environments
Source: BMC Genomics. 2020 Jan 29;21:90. doi: 10.1186/s12864-020-6522-3 (PMC6988325; doi:10.1186/s12864-020-6522-3)
Supplement: Supplementary file 20 — Additional file 20: Table S15. VIPs compounds (PTR-ToF data) according to PLS-DA analysis. [file 12864_2020_6522_MOESM20_ESM.docx]

**Table S15:** **VIPs compounds (PTR-ToF data) according to PLS-DA analysis.** Compounds that displayed VIP scores ≥ 2 (bold marked) in at least in one sample are shown. VOCs grouped based on their chemical class. The best VIP values are marked in yellow, for each selected compound. ^a^ Progressive code associated to each compound, as reported in Additional file 18: Table S13.

| **Compound list^a^** | **VIP Scores**  **for AL** | **VIP Scores**  **for IS** | **VIP Scores**  **for SM** | **Compound name** | **Chemical class** |
| --- | --- | --- | --- | --- | --- |
| ***9*** | **2.94** | **2.96** | 0.29 | ***ethanol*** | **Alcohols** |
| ***40*** | **3.90** | 1.32 | **2.20** | ***2-3-butanediol*** | **Alcohols** |
| ***62*** | 0.54 | **2.56** | **2.15** | ***n-heptanol*** | **Alcohols** |
| ***2*** | **2.49** | **2.51** | 0.84 | ***formaldehyde*** | **Aldehydes** |
| ***56*** | **2.72** | **2.31** | 0.61 | ***ethylbenzene*** | **Aromatic compounds** |
| ***5*** | **2.30** | 0.49 | **2.22** | ***alkyl fragment (ethenone)*** | **Ketones** |
| ***48*** | 1.08 | **4.41** | **2.30** | ***4-methyl-5h-furan-2-one*** | **Ketones** |
| ***19*** | **2.55** | 1.51 | **4.85** | ***methanetriol*** | **Others** |
| ***54*** | **2.53** | **3.03** | 0.42 | ***4-hydroxybutanoic acid*** | **Others** |
| ***59*** | 0.40 | **3.06** | 1.73 | ***2-acetylfuran*** | **Others** |
| ***15*** | 0.35 | **2.15** | 1.22 | ***sulfur compounds (acetylenethiol / thiirene)*** | **Sulfur-containing volatiles** |
| ***29*** | 0.58 | **2.64** | 1.94 | ***1-propanethiol*** | **Sulfur-containing volatiles** |
| ***30*** | 0.32 | **2.28** | 1.32 | ***sulfur compounds (dithioformic acid)*** | **Sulfur-containing volatiles** |
| ***44*** | 0.27 | **2.11** | 1.31 | ***dimethyl sulfone*** | **Sulfur-containing volatiles** |
| ***51*** | 0.77 | **4.39** | **3.03** | ***2-methyl-4,5-dihydrothiophene*** | **Sulfur-containing volatiles** |
