# Supplementary material for: Tuberomics: a molecular profiling for the adaption of edible fungi (Tuber magnatum Pico) to different natural environments
Source: BMC Genomics. 2020 Jan 29;21:90. doi: 10.1186/s12864-020-6522-3 (PMC6988325; doi:10.1186/s12864-020-6522-3)
Supplement: Supplementary file 21 — Additional file 21: Table S16. Climatic parameters on the fruiting bodies sampling area for the four-year period 2014–2017 used for statistical analysis of PTR-ToF data (CCoA). [file 12864_2020_6522_MOESM21_ESM.docx]

**Table S16: Climatic parameters on the fruiting bodies sampling area for the four-year period 2014-2017 used for statistical analysis of PTR-ToF data (CCoA).** Data were downloaded from climate stations close to sampling sites. AL (Alba Tanaro; https://www.arpa.piemonte.gov.it); IS (Isernia Colle Vecchio; https://www.meteoisernia.net); SM (San Miniato; http://www.sir.toscana.it: TOS01001491).

|  |  | **AL** | **IS** | **SM** |
| --- | --- | --- | --- | --- |
| ***Altitude (m)*** |  | 172 | 600 | 102 |
| ***Temperature (°C)*** | ***Jan*** | *3.43 ± 0.7* | *5.28 ± 2.26* | *7.68 ± 1.61* |
|  | ***Feb*** | *4.9 ± 1.34* | *6.55 ± 1.48* | *9.8 ± 1.23* |
|  | ***Mar*** | *9.07 ± 0.73* | *8.63 ± 1.11* | *11.98 ± 0.87* |
|  | ***Apr*** | *13.81 ± 0.2* | *11.73 ± 1.14* | *14.85 ± 0.5* |
|  | ***May*** | *17.23 ± 1.18* | *15.1 ± 1.16* | *18.08 ± 0.85* |
|  | ***Jun*** | *22.03 ± 0.62* | *19.38 ± 1.13* | *22.93 ± 1.23* |
|  | ***Jul*** | *24.77 ± 2.67* | *22.3 ± 1.97* | *25.28 ± 1.92* |
|  | ***Aug**** | *23 ± 0.93* | *22.33 ± 1.65* | *24.98 ± 1.6* |
|  | ***Sep**** | *19.16 ± 1.23* | *17.5 ± 0.9* | *20.65 ± 1.31* |
|  | ***Oct**** | *13.3 ± 1.45* | *13.83 ± 0.72* | *16.68 ± 1.09* |
|  | ***Nov**** | *8.31 ± 1.25* | *9.88 ± 1.28* | *12.15 ± 1.59* |
|  | ***Dec*** | *3.8 ± 1.15* | *6.15 ± 0.9* | *8.55 ± 1.13* |
| ***Rainfall (mm)*** | ***Jan*** | *29.4 ± 19.8* | *94.73 ± 45.88* | *104.9 ± 85.75* |
|  | ***Feb*** | *95.13 ± 47.59* | *96.1 ± 39.4* | *138.95 ± 66.56* |
|  | ***Mar*** | *117.2 ± 48.6* | *61.3 ± 23.89* | *62.15 ± 19.85* |
|  | ***Apr*** | *37.13 ± 12.72* | *51.1 ± 28.65* | *60.1 ± 37.61* |
|  | ***May*** | *67 ± 13.71* | *50.93 ± 35.89* | *51.4 ± 41.32* |
|  | ***Jun*** | *62.2 ± 24.36* | *37.38 ± 44.51* | *46.1 ± 29.4* |
|  | ***Jul*** | *45.4 ± 49.76* | *31.95 ± 33.77* | *40.45 ± 64.93* |
|  | ***Aug**** | *35.53 ± 8.54* | *13.08 ± 18.82* | *36 ± 42.25* |
|  | ***Sep**** | *18.93 ± 12.01* | *58.23 ± 51.6* | *94.05 ± 71.88* |
|  | ***Oct**** | *57.93 ± 34.34* | *42.78 ± 39.53* | *87.8 ± 78.73* |
|  | ***Nov**** | *127.13 ± 112.77* | *119.18 ± 24.5* | *110.1 ± 67.58* |
|  | ***Dec*** | *55.47 ± 44.75* | *77.5 ± 83.28* | *53.75 ± 55.22* |

*red marked data were used for CCoA analysis.
